# Crystal structures of four new iridium complexes, each containing a highly flexible carbodi­phos­phorane PCP pincer ligand

**DOI:** 10.1107/S2056989018007569

**Published:** 2018-05-25

**Authors:** Gabriel Julian Partl, Felix Nussbaumer, Inge Schlapp-Hackl, Walter Schuh, Holger Kopacka, Klaus Wurst, Paul Peringer

**Affiliations:** a Institute of General, Inorganic, and Theoretical Chemistry, University of Innsbruck, Innrain 80-82, A-6020 Innsbruck, Austria

**Keywords:** crystal structure, iridium, PCP pincer ligand, carbodi­phospho­rane, hydride, carbon­yl, cyclo­octa­diene, oxidative addition

## Abstract

Compound [Ir(C_8_H_12_)(C_51_H_45_P_4_)]Cl_2_ or [Ir(cod)(CH(dppm)_2_-κ^3^ *P*,*C*,*P*)]Cl_2_ (**1a**), was obtained from [IrCl(cod)]_2_ and the carbodi­phospho­rane (CDP) salt [CH(dppm)_2_]Cl [where cod = cyclo­octa-1,5-diene and dppm = bis­(di­phenyl­phosphino)methane]. Treatment of **1a** with thallium(I) tri­fluoro­methane­sulfonate [Tl(OTf)] and subsequent crystallization gave complex [Ir(C_8_H_12_)(C_51_H_45_P_4_)](OTf)_2_·CH_3_CO_2_C_2_H_5_·CH_2_Cl_2_ or [Ir(cod)(CH(dppm)_2_-κ^3^ *P*,*C*,*P*)](OTf)_2_·CH_3_CO_2_C_2_H_5_·CH_2_Cl_2_ (**1b**) [systematic name: (cyclo­octa-1,5-diene)(1,1,3,3,5,5,7,7-octa­phenyl-1,7-diphospha-3,5-di­phospho­niaheptan-4-yl)iridium(I) bis­(tri­fluoro­methane­sulfonate)–ethyl acetate–di­chloro­methane (1/1/1)]. This five-coordinate iridium(I) complex cation adopts a trigonal–bipyramidal geometry with the CDP carbon and one cod double bond in axial sites. Compound **1b** represents the first example of a non-*meridional* coordination of the PCP pincer ligand [CH(dppm)_2_]^+^ with a P—Ir—P angle of 98.08 (2)°. Compound **2**, [IrCl_2_H(C_51_H_44_P_4_)]·(CH_3_)_2_CO or [IrCl_2_H(C(dppm)_2_-κ^3^ *P*,*C*,*P*)]·(CH_3_)_2_CO [systematic name: di­chlorido­hydrido(1,1,3,3,5,5,7,7-octa­phenyl-1,5λ^5^,7-triphospha-3-phospho­niahept-4-en-4-yl)iridium(III) acetone monosolvate], crystallizes as an acetone monosolvate. It is a six-coordinate Ir^III^ coordination compound. Here, the PCP pincer ligand is coordinated in a *meridional* manner; one chlorido ligand is positioned *trans* to the carbon donor, the remaining two coordination sites being occupied by the second chlorido and a hydrido ligand *trans* to each other. Complex **3**, [IrCl_2_H(C_51_H_45_P_4_)]Cl·5H_2_O or [IrCl_2_H(CH(dppm)_2_-κ^3^ *P*,*C*,*P*)]Cl·5H_2_O [systematic name: di­chlorido­hydrido(1,1,3,3,5,5,7,7-octa­phenyl-1,7-diphospha-3,5-di­phospho­niaheptan-4-yl)iridium(III) chloride penta­hydrate], represents the conjugate CH acid of **2**. The ligand [CH(dppm)_2_]^+^ is coordinated in a *meridional* manner. In the cationic six-coordinate Ir^III^ complex **4**, [IrClH(CO)(C_51_H_44_P_4_)]Cl·2CH_3_OH·H_2_O or [IrClH(CO)(C(dppm)_2_-κ^3^ *P*,*C*,*P*)]Cl·2CH_3_OH·H_2_O [systematic name: carbonyl­chlorido­hydrido(1,1,3,3,5,5,7,7-octa­phenyl-1,5λ^5^,7-triphospha-3-phos­pho­niahept-4-en-4-yl)iridium(III) chloride–methanol–water (1/2/1)], the chlorido ligand is found in the plane defined by the Ir center and the *meridional* PCP ligand; the H and CO ligands are positioned axially to this plane and *trans* to each other.

## Abstract 2

The synthesis and crystal structures of four iridium–PCP pincer complexes, each containing a highly flexible carbodi­phospho­rane PCP pincer ligand, are discussed.

## Chemical context   

The syntheses of the title compounds are summarized in the Scheme. The substitution of the bridging chlorido ligands of [IrCl(cod)]_2_ by the cationic PCP pincer ligand [CH(dppm)_2_]Cl qu­anti­tatively affords the five-coordinate Ir(I) PCP pincer complex [Ir(cod)(CH(dppm)_2_- κ^3^P,C,P)]Cl_2_ (**1a**). The central carbon of the PCP ligand is part of a protonated carbodi­phospho­rane (CDP) functionality. Metathesis with Tl(OTf) gave the corresponding OTf salt (**1b**). These products represent the first examples of a *non-meridional* coordination mode of the PCP pincer ligand [CH(dppm)_2_]^+^.
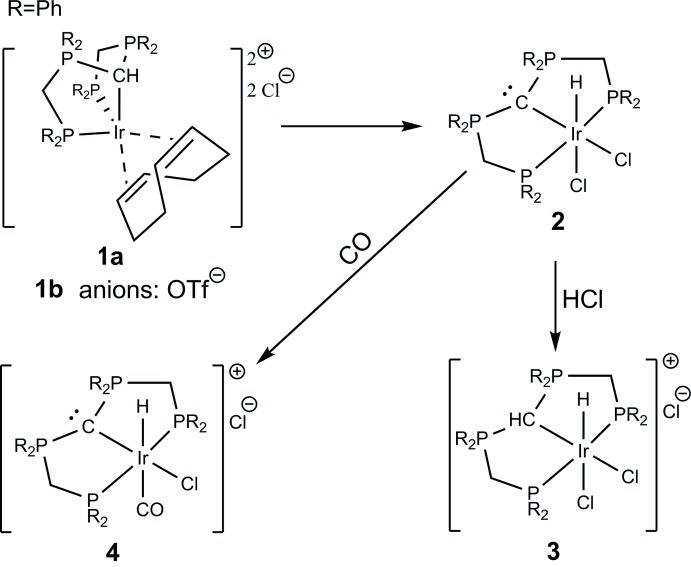


Related Ir^I^ complexes of the composition [Ir(PCP)(cod)]^*n*+^ have been reported for a neutral PCP ligand based on a NHO type framework (*n* = 1; Iglesias *et al.*, 2015 ▸), for the anionic aryl based ligand [C_6_H_3_-1,3-[CH_2_P(CF_3_)_2_]_2_]^−^ (*n* = 0; Adams *et al.*, 2011 ▸), and an anionic asymmetric PC(*sp^3^*)P ligand (*n* = 0; Cui *et al.*, 2016 ▸). They were obtained either analogously to **1a** (Iglesias *et al.*, 2015 ▸; Cui *et al.*, 2016 ▸) or *via* a combined reductive elimination/substitution reaction of [IrClH(PCP)(C_2_H_4_)] with NEt_3_ in the presence of cod (Adams *et al.*, 2011 ▸).

**Figure 1:**
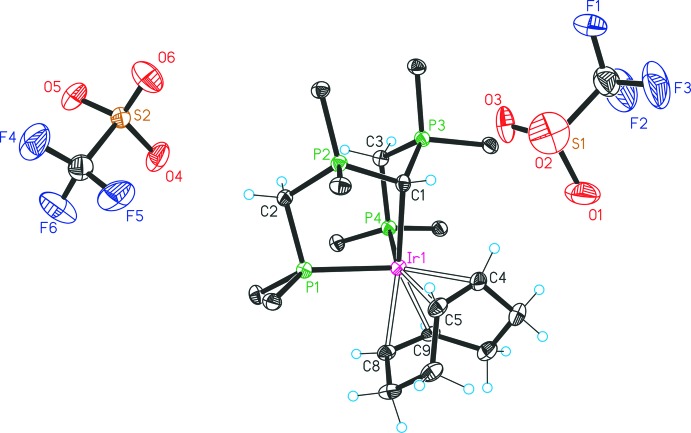
Structure of compound **1b**, with atom labelling and 30% probability displacement ellipsoids. For clarity, only the *ipso* carbon atoms of the phenyl groups are shown, and the solvent mol­ecules have been omitted.

Whilst the complex **1b** is indefinitely stable, **1a** qu­anti­tatively transforms into the Ir^III^ PCP pincer CDP complex [IrCl_2_H(C(dppm)_2_-κ^3^P,C,P)] (**2**), *via* an intra­molecular oxidative addition reaction upon prolonged standing in solution (Fig. 1 ▸). The sole reported Ir complex with a donor set related to **2** is [IrCl_2_H(PCP)]NHEt_3_, involving the above mentioned π-accepting anionic ligand [C_6_H_3_-1,3-[CH_2_P(CF_3_)_2_]_2_]^−^ (Adams *et al.*, 2011 ▸). This ligand is able to adopt both *meridional* and *non-meridional* coordination modes related to the cationic protonated PCP pincer CDP ligand [CH(dppm)_2_]^+^.

The central carbon of CDPs carries two lone electron pairs and is able to inter­act with one or two Lewis acids (Petz & Frenking, 2010 ▸). Consequently, the central carbon of the PCP pincer ligand of **2** is able to inter­act with another Lewis acid and can be converted to the conjugate CH acid [IrCl_2_H(CH(dppm)_2_-κ^3^P,C,P)]Cl (**3**), upon treatment with aqueous hydro­chloric acid.

The reaction of **2** with carbon monoxide results in the substitution of the chlorido ligand positioned *trans* to the hydrido ligand and affords [IrClH(CO)(CH(dppm)_2_-κ^3^P,C,P)]Cl (**4**). The isomer of **4** with the CO ligand positioned *trans* to the carbodi­phospho­rane carbon of the PCP pincer ligand has been synthesized *via* reaction of Vaska’s complex with [CH(dppm)_2_]Cl (Reitsamer *et al.*, 2018 ▸). Related [IrClH(CO)(PCP)] complexes with the H and CO ligands in a *trans* configuration have been obtained *via* addition of CO to the corresponding five-coordinated complexes [IrClH(PCP)] (Goldberg *et al.*, 2015 ▸; Segawa *et al.*, 2009 ▸; Jonasson *et al.*, 2015 ▸; Kuklin *et al.*, 2006 ▸), or, in one case, by bubbling CO through a solution of [IrClH(MeCN)(PCP)] in di­chloro­methane, with the H and Cl ligands being in a *trans* configuration (Silantyev *et al.*, 2014 ▸). Both isomers of [IrClH(CO)(PCP)], either with H and CO or H and Cl in a *trans* configuration have been structurally characterized for a triptycene-based PCP pincer ligand (Silantyev *et al.*, 2014 ▸; Azerraf & Gelman, 2009 ▸) and a cyclo­hexyl-based PCP pincer ligand (Jonasson *et al.*, 2015 ▸).

## Structural commentary   

The mol­ecular structures of the four complexes are illustrated in Figs. 1 ▸–4 ▸ ▸ ▸, and selected bond distances and bond angles are given in Table 1 ▸. The structure of **1b** (Fig. 1 ▸) establishes an 18-electron five-coordinate dicationic Ir^I^ complex with two OTf^−^ counter-ions. The Ir atom is coordinated by the PCP pincer ligand [CH(dppm)_2_]^+^ and a bidentate cod ligand in a distorted trigonal–bipyramidal geometry, in which the *axial* positions are occupied by the CDP carbon C1 and the double bond C8=C9 of the cod ligand; the donor atoms P1 and P4 and the double bond C4=C5 are located in the *equatorial* sites. The P1—Ir1—P4 angle amounts to 98.08 (2)°, compared to 102.789 (19)° (Cui *et al.*, 2016 ▸), 106.44 (3)° (Iglesias *et al.*, 2015 ▸) and 119.02 (4)° (Adams *et al.*, 2011 ▸) for the aforementioned Ir^I^ related compounds [Ir(PCP)(cod)]^*n*+^. The PCP pincer ligand [CH(dppm)_2_]^+^ is an impressively flexible ligand, adopting a range of P—Ir—P values from 98.08 (2)° for **1b** to 177.66 (4)° observed for [(PtCl)(CH(dppm)_2_-κ^3^P,C,P)]Cl_2_ (Reitsamer *et al.*, 2012 ▸). The Ir1—C1 distance of 2.232 (3) Å is found in the upper segment of Ir—C distance ranges, as is typical for Ir complexes involving the [CH(dppm)_2_]^+^ ligand (Reitsamer *et al.*, 2012 ▸). The P—C ‘separation sizes’ within the CDP functionality are in the range of single bonds, as expected for CDPs donating to two Lewis acids (Petz & Frenking, 2010 ▸). The geometry around C1 is distorted tetra­hedral according to the angles P3—C1—P2 = 114.86 (14)°, P3—C1—Ir1 = 109.99 (13)° and P2—C1—Ir1 = 111.40 (13)°.

**Figure 2:**
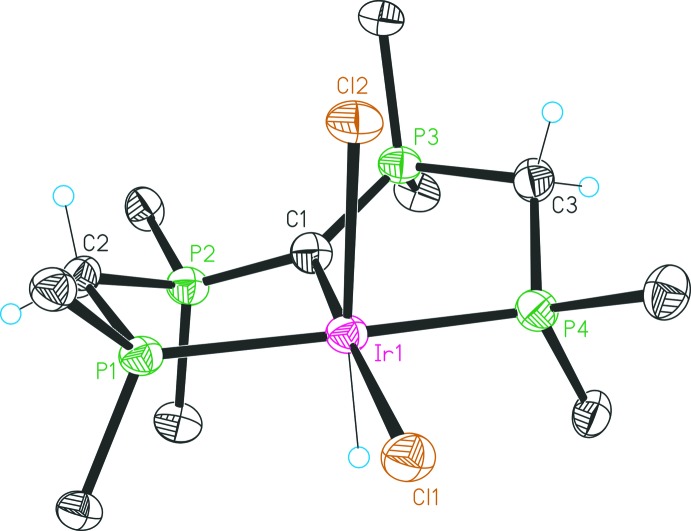
Structure of compound **2**, with atom labelling and 30% probability displacement ellipsoids. For clarity, only the *ipso* carbon atoms of the phenyl groups are shown, and the solvent mol­ecules have been omitted.

The structure of **2** (Fig. 2 ▸) consists of an octa­hedral Ir^III^ coordination compound. The Ir center is coordinated by the PCP pincer, one hydrido and two chlorido ligands. The [C(dppm)_2_] unit coordinates in a *meridional* manner; the Cl1 ligand is located *trans* to the central CDP carbon C1, ligands H1 and Cl2 are positioned normal to this plane and are *trans* to each other. The Ir1—C1 bond length amounts to 2.101 (5) Å and is comparatively short according to the weak *trans* influence of a chlorido ligand. With a P4—Ir1—P1 angle of 173.09 (5)°, [C(dppm)_2_] also showcases high structural flexibility. Both the planar environment of C1 and the P—C bond lengths within the CDP functionality are in keeping with CDPs inter­acting with one Lewis acid (Petz & Frenking, 2010 ▸). The configuration of the two five-membered rings of the PCP pincer system is somewhat dissimilar, as evidenced by a comparison of the corresponding angles which differ up to *ca* 8° (see Table 1 ▸).

**Figure 3:**
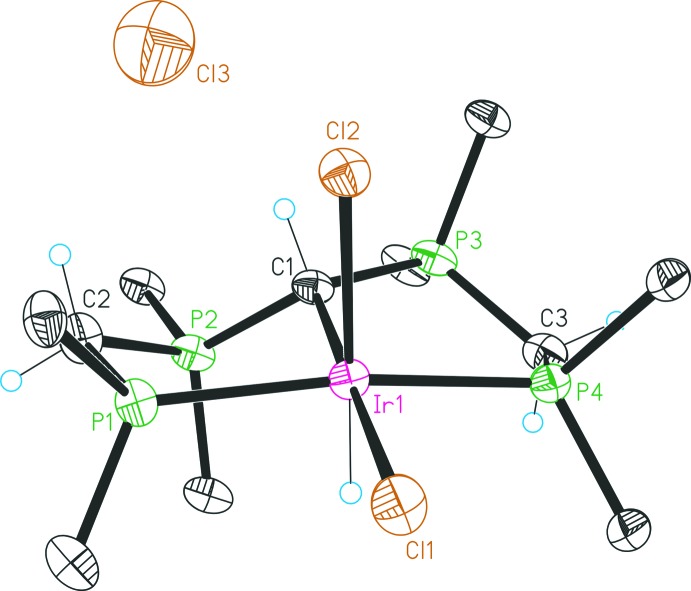
Structure of compound **3**, with atom labelling and 30% probability displacement ellipsoids. For clarity, only the *ipso* carbon atoms of the phenyl groups are shown, and the solvent mol­ecules have been omitted.

The structure of **3** (Fig. 3 ▸) exhibits a [IrCl_2_H(CH(dppm)_2_-κ^3^P,C,P)]^+^ complex cation, accompanied by a chloride counter-ion. Protonation of the CDP carbon C1 results in a distorted tetra­hedral environment. The bond angles P2—C1—P3, P2—C1–Ir1 and P3—C1—Ir1 are reduced by *ca* 5–7°, as compared to the values for compound **2**. As expected, due to protonation, the C1–P2/P3 bond lengths are now characteristic of P—C single bonds (Petz & Frenking, 2010 ▸). The orientation of the proton on C1 relative to the hydrido ligand H1 is *anti-periplanar*. Protonation of the CDP carbon yields a heterogeneous effect on Ir-donor distances: while the Ir1—C1 bond length is longer than in **2** [2.132 (4) Å *cf*. 2.101 (5) Å], the Ir1—Cl1 bond length is shorter [2.405 (1) Å *cf*. 2.441 (2) Å]. The two rings of the PCP pincer system are different as has been emphasized for compound **2**.

**Figure 4:**
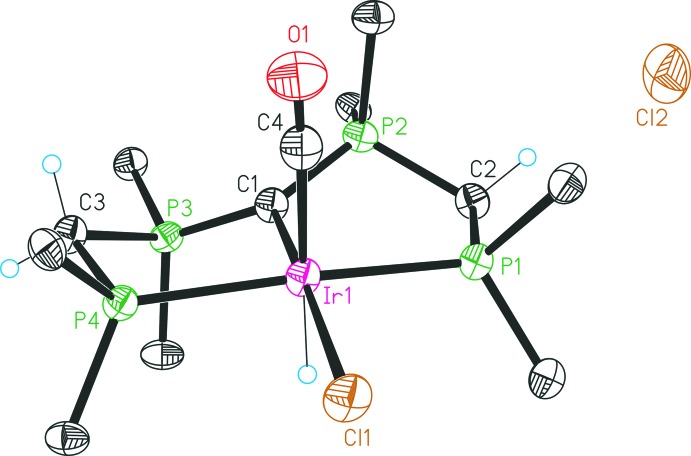
Structure of compound **4**, with atom labelling and 30% probability displacement ellipsoids. For clarity, only the *ipso* carbon atoms of the phenyl groups are shown, and the solvent mol­ecules have been omitted.

The structure of compound **4** consists of a [IrClH(CO)(C(dppm)_2_-κ^3^P,C,P)]^+^ complex cation and a chloride counter-ion (Fig. 4 ▸). In **4**, the Ir atom is coordinated by the PCP pincer in a *meridional* mode, with one chlorido ligand *trans* to the central CDP carbon atom and one hydrido and one carbonyl ligand *trans* to each other. Compared to compound **2**, the CO ligand causes a lengthening of the Ir1—C1 and the Ir—P bonds, while both the Ir1—C1 and the Ir1—H1 bonds are shortened (Table 1 ▸). In contrast to **2** and **3**, the angles formed by the two rings of the pincer system are quite similar. The planarity around atom C1 and the C1—P2/P3 bond lengths confirms a CDP with one Lewis acid attached.

## Supra­molecular features   

**Figure 5:**
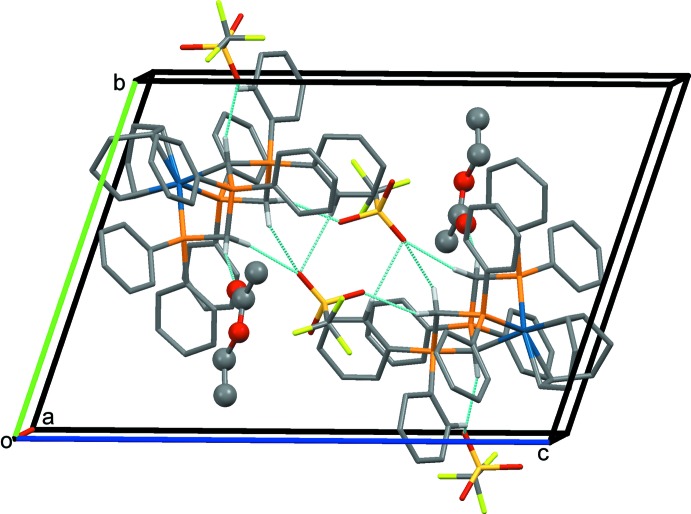
A view along the *a* axis of the crystal packing of compound **1b**. Only the H atoms involved in the most significant inter­molecular inter­actions (Table 2 ▸) have been included. The ethyl acetate solvate mol­ecule is shown in ball-and-stick mode.

In all four crystal structures the CH_2_ groups and the central CH group of the [CH(dppm)_2_]^+^ unit inter­act with solvate mol­ecules and anions. It has been pointed out that such C—H⋯*X* inter­actions are a common feature of complexes containing dppm or related ligands (Jones & Ahrens, 1998 ▸). The most significant hydrogen-bonding inter­actions in the crystals of the four compounds are given in Tables 2 ▸–5 ▸ ▸ ▸, and illustrated in Figs. 5 ▸–8 ▸ ▸ ▸.

In the crystal of **1b** (Fig. 5 ▸), two neighbouring mol­ecules are linked *via* C—H⋯O hydrogen bonds involving two O atoms (O4 and O5) of two inversion-related OTf^−^ anions. Each complex cation is linked to the ethyl acetate solvate mol­ecule by a C3*A*—H3*A*⋯O7 hydrogen bond and to the other OTf^−^ anion by three (trifurcated) C—H⋯O3 hydrogen bonds.

**Figure 6:**
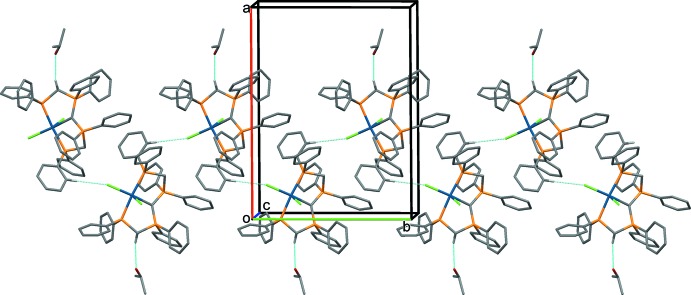
A view along the *c* axis of the crystal packing of compound **2**. Only the H atoms involved in the most significant inter­molecular inter­actions (Table 3 ▸) have been included.

In the crystal of **2** (Fig. 6 ▸), mol­ecules are linked by C—H⋯Cl hydrogen bonds, forming a 2_1_ helix propagating along the *b*-axis direction. The acetone solvate mol­ecule is linked to the complex mol­ecule by a C—H⋯O hydrogen bond.

**Figure 7:**
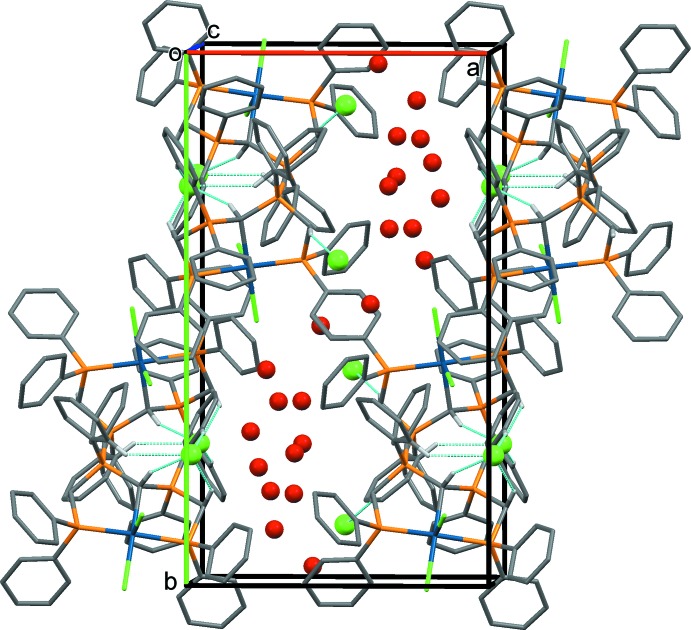
A view along the *c* axis of the crystal packing of compound **3**. Only the H atoms involved in the most significant inter­molecular inter­actions (Table 4 ▸) have been included. The free Cl^−^ anions and the disordered water mol­ecules are shown in ball-and-stick mode.

In the crystal of **3** (Fig. 7 ▸), the free Cl^−^ anion is linked to the complex cation by three C—H⋯Cl hydrogen bonds.

**Figure 8:**
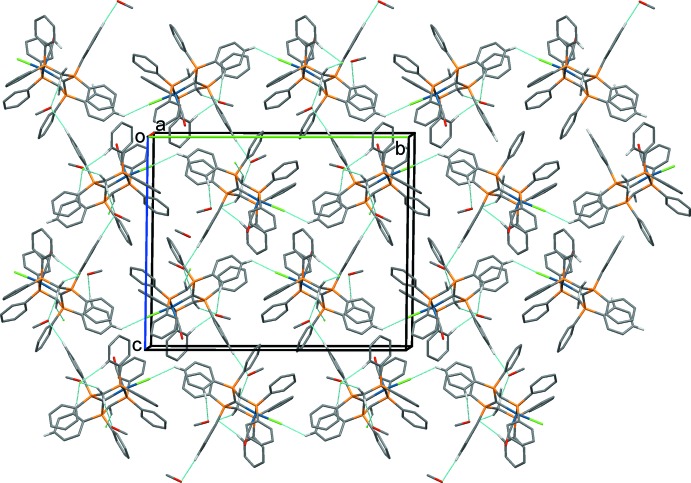
A view along the *a* axis of the crystal packing of compound **4**. Only the H atoms involved in the most significant inter­molecular inter­actions (Table 5 ▸) have been included.

In the crystal of **4** (Fig. 8 ▸), mol­ecules are linked by C—H⋯Cl hydrogen bonds, forming a 2_1_ helix propagating along the *b*-axis direction, similar to the situation in the crystal of **2**. The helices are linked by a methanol solvate mol­ecule (O2), forming layers parallel to the *bc* plane. Other inter­molecular inter­actions involve the Cl^−^ anions and the methanol solvate mol­ecules.

## Synthesis and crystallization   

The syntheses of the title compounds are summarized in the Scheme. All preparations were carried out under an inert atmosphere (N_2_) by the use of standard Schlenk techniques. The ^1^H, ^13^C and ^31^P NMR spectra were recorded on a Bruker DPX 300 NMR spectrometer (300 MHz) and were referenced against ^13^C/^1^H solvent peaks of the solvents or an external 85% H_3_PO_4_ standard, respectively. The phospho­rus atoms in the NMR data are labelled in the same way as in the figures.

**Synthesis of complexes 1a** and **1b:** [IrCl(cod)]_2_ (8.5 mg; 0.0125 mmol) and [CH(dppm)_2_]Cl (20.5 mg; 0.025 mmol) (Reitsamer *et al.*, 2012 ▸) were dissolved in CHCl_3_ (0.6 ml), whereupon **1a** formed instantaneously. Immediately after, a solution of TlOTf (17.7 mg; 0.05 mmol) in MeOH (0.1 ml) was added and the mixture was stirred for 15 min. The TlCl precipitate was removed and the volatiles evaporated *in vacuo*. Single crystals of **1b** were obtained by layering a solution of the residue in CH_2_Cl_2_ with EtOAc.

Spectroscopic data for **1a**: The [*AX*]_2_ pattern was simulated by use of the program *WINDAISY* (Weber *et al.*, 1993 ▸; Hägele *et al.*, 1988 ▸). ^31^P{^1^H} NMR (CHCl_3_, referenced against external 85% H_3_PO_4_, numbering as in the crystal structure): δ = 48.7 (P2/P3, [*AX*]_2_, *J*P2P3 = 32.5 Hz; *J*P2P4 = 18.1 Hz, *J*P1P2 = 87.4 Hz); −12.7 (P1/P4, [*AX*]_2_, *J*P1P4 = 11.1 Hz) ppm. ^13^C NMR (CDCl_3_, referenced against ^13^C solvent peak): δ = −8.0 (C1, *dtt*, *J*C1P2/P3 = 24.5 Hz, *J*C1P1/P4 = 4.6 Hz, *J*C1H1 = 131.8 Hz) ppm.

**Synthesis of compound 2**: [IrCl(cod)]_2_ (8.5 mg; 0.0125 mmol) and [CH(dppm)_2_]Cl (20.5 mg; 0.025 mmol) were dissolved in acetone (0.6 ml). Orange crystals formed upon keeping the solution at 277 K. ^31^P{^1^H} NMR (CHCl_3_): δ = 25.3 (P2/3, *vt*, N = 65.7 Hz); 1.3 (P1/P4, *vt*) ppm. ^13^C{^1^H} NMR (CDCl_3_): δ = −34.7 (C1, *tt*, *J*C1P2/P3 = 90.2 Hz), *J*(C1P1/P4 = 2.0 Hz) ppm. ^1^H NMR (CDCl_3_): δ = −24.4 (H1, *t*, *J*H1P1/P4 = 13.0 Hz) ppm.

**Synthesis of complex 3:** A mixture of [IrCl(cod)]_2_ (8.5 mg; 0.0125 mmol), [CH(dppm)_2_]Cl (20.5 mg; 0.025 mmol), CHCl_3_ (0.6 mL) and hydro­chloric acid (0.1 ml, 4 mol/l) was agitated for 24 h. Colourless crystals were obtained by keeping the mixture at 277 K. ^31^P{^1^H} NMR (CHCl_3_): δ = 45.2 (P2/P3, *vt*, N = 62.7 Hz); −4.9 (P1/P4, *vt*) ppm. ^13^C NMR (CDCl_3_): δ = −4.5 (C1, *dt*, *J*C1P2/P3 = 38.2 Hz, *J*C1H1*A* = 123.7 Hz) ppm. ^1^H NMR (CDCl_3_): δ = −22.1 (H1, *t*, *J*H1P1/P4 = 13 Hz) ppm.

**Synthesis of complex 4**: [IrCl(cod)]_2_ (8.5 mg; 0.0125 mmol) and [CH(dppm)_2_]Cl (20.5 mg; 0.025 mmol) were placed under an atmosphere of CO and dissolved in CH_2_Cl_2_ (0.8 ml). The mixture was agitated for 24 h, followed by volatiles evaporation *in vacuo*. The residue was extracted with MeOH once, the insoluble fraction then dissolved in MeOH/CH_2_Cl_2_. Colourless crystals were formed on slow evaporation of this solution. ^31^P{^1^H} NMR (CHCl_3_): δ = 32.5 (P2/P3, *vt*, N = 61.0 Hz); −3.9 (P1/P4, *vt*) ppm. ^13^C{^1^H} NMR (CD_3_CN): δ = −26.5 (C1, *t*, *J*C1P2/P3) = 107 Hz) ppm. ^1^H NMR (CDCl_3_): δ = −7.6 (H1, *dt*, *J*H1P1/P4 = 13.0 Hz, *J*H1C4 = 54.0 Hz) ppm.

## Refinement   

Crystal data, data collection and structure refinement details are summarized in Table 6 ▸. The hydrogen atoms at C1, C4=C5 and C8=C9 of **1b** were located in a difference-Fourier map and refined with bond restraints (C—H = 0.96 Å for H1 and 0.93 Å for H4, H5, H8 and H9). Both OTf^−^ anions show positional disorder in the occupancy ratio of 0.7:0.3. The solvent mol­ecule CH_2_Cl_2_ also shows positional disorder with the ratio 0.7:0.3; the hydrogen atoms of this disordered mol­ecule were omitted.

In **2**, the metal-bound hydrogen atom was located in a difference-Fourier map and refined with the bond restraint Ir—H = 1.6 Å, since free refinement resulted in an unrealistically long bond distance of 1.88 Å. The solvent acetone mol­ecule is slightly disordered with a solved positional disorder for one methyl group, namely C6:C6*A* (ratio 0.5:0.5). Solvent hydrogen atoms could not be localized and were omitted.

In **3**, positional disorder of the anion Cl3:Cl3*A* was found in an occupancy ratio of 0.667:0.333. Hydrogen atoms H1 and H1*A* were located in a difference-Fourier map and freely refined. The water solvent mol­ecules show higher temperature factors and are slightly disordered, but this disorder was not solved; therefore the oxygen atoms (O5 and O6 with half occupancy) were refined isotropically and their hydrogen atoms were omitted.

In **4**, atom H1 was located in a difference-Fourier map and refined with bond restraint Ir—H = 1.6 Å. Hydrogen atoms of the MeOH and H_2_O solvate mol­ecules were omitted. One chloride anion is positionally disordered with an occupancy ratio of 0.5:0.5 for Cl2 and Cl2*A*. Possibly because of this disorder, two MeOH positions C6—O3 and C7—O4 are only half occupied; also, a water mol­ecule is split over four positions (O5, O5*A*, O5*B* and O5*C*) with an occupancy of 0.25 for each; they were refined isotropically.

The intensity data for compounds **1b**, **2** and **4**, were measured using a Nonius Kappa CCD diffractometer and no absorption corrections were applied. The intensity data for compound **3** was measured using a Bruker D8 Quest PHOTON 100 diffractometer and a multi-scan absorption correction was applied. The crystals used were extremely thin plates in all cases and the values of the residual electron density in the final difference-Fourier maps are satisfactory for complexes of such a heavy atom.

## Supplementary Material

Crystal structure: contains datablock(s) global, 1b, 2, 3, 4. DOI: 10.1107/S2056989018007569/su5440sup1.cif

Structure factors: contains datablock(s) 1b. DOI: 10.1107/S2056989018007569/su54401bsup4.hkl

Structure factors: contains datablock(s) 2. DOI: 10.1107/S2056989018007569/su54402sup5.hkl

Structure factors: contains datablock(s) 3. DOI: 10.1107/S2056989018007569/su54403sup2.hkl

Structure factors: contains datablock(s) 4. DOI: 10.1107/S2056989018007569/su54404sup3.hkl

CCDC references: 1837857, 1837858, 1837859, 1837860

Additional supporting information:  crystallographic information; 3D view; checkCIF report

## Figures and Tables

**Table 1:** Selected bond lengths (Å) and bond angles (°) for compounds **1b**–**4**

	**1b**	**2**	**3**	**4**
Ir1—C1	2.232 (3)	2.101 (5)	2.132 (4)	2.124 (5)
Ir1—C4	2.169 (3)	–	–	–
Ir1—C5	2.172 (3)	–	–	–
Ir1—C8	2.208 (3)	–	–	–
Ir1—C9	2.225 (3)	–	–	–
Ir1—Cl1	–	2.4412 (15)	2.405 (1)	2.4359 (13)
Ir1—P1	2.3889 (7)	2.3019 (15)	2.306 (1)	2.3265 (14)
Ir1—P4	2.3386 (7)	2.2831 (16)	2.283 (1)	2.3235 (13)
Ir1—H1	–	1.638 (19)	1.46 (4)	1.535 (19)
P2—C1	1.821 (3)	1.695 (6)	1.811 (4)	1.681 (5)
P3—C1	1.811 (3)	1.688 (6)	1.803 (4)	1.686 (5)
C4—C5	1.414 (4)	–	–	–
C8—C9	1.402 (4)	–	–	–
P2—C1—P3	114.86 (14)	127.1 (3)	120.60 (19)	130.4 (3)
P2—C1—Ir1	111.4 (1)	112.3 (3)	107.68 (17)	114.9 (3)
P3—C1—Ir1	110.0 (1)	120.4 (3)	114.38 (19)	114.5 (3)
P1—C2—P2	107.1 (1)	105.4 (3)	107.8 (2)	107.9 (3)
P3—C3—P4	105.7 (1)	110.0 (3)	108.5 (2)	106.6 (3)
P1—Ir1—P4	98.08 (2)	173.09 (5)	170.68 (4)	171.47 (5)
C1—Ir1—P1	88.27 (7)	89.23 (16)	88.33 (11)	87.18 (14)
C1—Ir1—P4	86.97 (7)	84.28 (16)	90.24 (11)	87.15 (14)
C2—P1—Ir1	104.80 (9)	106.4 (2)	105.58 (14)	107.45 (17)
C1—P2—C2	106.7 (1)	106.8 (3)	99.93 (18)	106.3 (2)
C1—P3—C3	104.1 (1)	106.2 (3)	107.99 (18)	104.8 (2)
C3—P4—Ir1	107.96 (9)	106.1 (2)	106.55 (14)	106.83 (17)

**Table 2:** Hydrogen-bond geometry (Å, °) for **1b** [Chem scheme1]

*D*—H⋯*A*	*D*—H	H⋯*A*	*D*⋯*A*	*D*—H⋯*A*
C2—H2*B*⋯O4	0.98	2.36	3.245 (4)	151
C2—H2*A*⋯O5^i^	0.98	2.38	3.307 (4)	158
C3—H3*B*⋯O5^i^	0.98	2.38	3.343 (4)	169
C206—H206⋯O5^i^	0.94	2.52	3.204 (4)	130
C3—H3*A*⋯O7	0.98	2.23	3.185 (5)	165
C1—H1⋯O3	0.94 (2)	2.55 (2)	3.419 (11)	155 (2)
C208—H208⋯O3	0.94	2.47	3.231 (16)	139
C308—H308⋯O3	0.94	2.57	3.301 (12)	135

Symmetry code: (i) 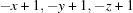.

**Table 3:** Hydrogen-bond geometry (Å, °) for **2** [Chem scheme1]

*D*—H⋯*A*	*D*—H	H⋯*A*	*D*⋯*A*	*D*—H⋯*A*
C112—H112⋯Cl1^i^	0.94	2.73	3.601 (6)	154
C3—H3*B*⋯O1	0.98	2.48	3.435 (10)	163

Symmetry code: (i) 
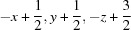
.

**Table 4:** Hydrogen-bond geometry (Å, °) for **3** [Chem scheme1]

*D*—H⋯*A*	*D*—H	H⋯*A*	*D*⋯*A*	*D*—H⋯*A*
C1—H1*A*⋯Cl3	0.99 (4)	2.48 (4)	3.422 (6)	159 (3)
C2—H2*B*⋯Cl3	0.98	2.63	3.450 (6)	142
C202—H202⋯Cl3	0.94	2.67	3.584 (6)	166
C308—H308⋯Cl3	0.94	2.70	3.475 (7)	141
C3—H3*B*⋯Cl3*A*	0.98	2.58	3.489 (12)	155

**Table 5:** Hydrogen-bond geometry (Å, °) for **4** [Chem scheme1]

*D*—H⋯*A*	*D*—H	H⋯*A*	*D*⋯*A*	*D*—H⋯*A*
C304—H304⋯Cl1^i^	0.94	2.76	3.465 (7)	132
C2—H2*B*⋯O2	0.98	2.43	3.407 (8)	173
C210—H210⋯O2^ii^	0.94	2.59	3.384 (10)	143
C2—H2*A*⋯Cl2	0.98	2.71	3.633 (7)	157
C112—H112⋯Cl2	0.94	2.76	3.682 (8)	168
C3—H3*A*⋯Cl2*A*	0.98	2.69	3.569 (8)	150
C302—H302⋯O3	0.94	2.49	3.414 (14)	169

Symmetry codes: (i) 
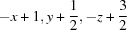
; (ii) 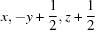.

**Table 6:** Experimental details

	**1b**	**2**	**3**	**4**
Crystal data				
Chemical formula	[Ir(C_51_H_45_P_4_)(C_8_H_12_)](CF_3_SO_3_)_2_·CH_3_CO_2_C_2_H_5_·CH_2_Cl_2_	[Ir(C_51_H_44_P_4_)ClH]Cl·C_3_H_6_O	[Ir(C_51_H_45_P_4_)ClH]Cl·5H_2_O	[Ir(C_51_H_44_P_4_)ClH(CO)]Cl·2CH_4_O·H_2_O
*M*_r_	1553.30	1102.93	1171.38	1154.96
Crystal system, space group	Triclinic, *P* 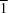	Monoclinic, *P*2_1_/*n*	Monoclinic, *P*2_1_/*c*	Monoclinic, *P*2_1_/*c*
Temperature (K)	233	233	203	233
*a*, *b*, *c* (Å)	13.3105 (2), 14.3109 (3), 19.8482 (3)	18.7964 (4), 13.7444 (2), 18.8487 (4)	12.6532 (8), 21.8847 (12), 19.9228 (12)	12.5929 (2), 23.2803 (4), 19.7488 (4)
α, β, γ (°)	68.949 (1), 74.426 (1), 70.256 (1)	90, 101.586 (2), 90	90, 99.381 (2), 90	90, 107.535 (1), 90
*V* (Å^3^)	3274.1 (1)	4770.25 (16)	5443.1 (6)	5520.66 (17)
*Z*	2	4	4	4
Radiation type	Mo *K*α	Mo *K*α	Mo *K*α	Mo *K*α
μ (mm^−1^)	2.35	3.08	2.76	2.67
Crystal size (mm)	0.21 × 0.10 × 0.06	0.15 × 0.12 × 0.02	0.17 × 0.12 × 0.09	0.21 × 0.10 × 0.07

Data collection				
Diffractometer	Nonius KappaCCD	Nonius KappaCCD	Bruker D8 QUEST PHOTON 100	Nonius KappaCCD
Absorption correction	–	–	Multi-scan (*SADABS*; Bruker, 2015 ▸)	–
*T*_min_, *T*_max_	–	–	0.691, 0.801	–
No. of measured, independent and observed [*I* > 2σ(*I*)] reflections	20473, 11235, 10195	27235, 8407, 6069	104100, 10577, 9522	32186, 9675, 8070
*R*_int_	0.027	0.083	0.031	0.049
(sin θ/λ)_max_ (Å^−1^)	0.591	0.595	0.615	0.594

Refinement				
*R*[*F* ^2^ > 2σ(*F* ^2^)], *wR*(*F* ^2^), *S*	0.025, 0.055, 1.03	0.048, 0.091, 1.04	0.032, 0.095, 1.09	0.041, 0.095, 1.13
No. of reflections	11235	8407	10577	9675
No. of parameters	976	572	574	624
No. of restraints	5	1	0	1
H-atom treatment	H atoms treated by a mixture of independent and constrained refinement	H-atom parameters constrained	H atoms treated by a mixture of independent and constrained refinement	H atoms treated by a mixture of independent and constrained refinement
Δρ_max_, Δρ_min_ (e Å^−3^)	0.58, −0.68	0.92, −0.81	1.27, −0.87	1.85, −1.45

Computer programs: *COLLECT* (Nonius, 1998 ▸), *APEX2* and *SAINT* (Bruker, 2015 ▸), *DENZO* and *SCALEPACK* (Otwinowski & Minor, 1997 ▸), *SHELXS97* and *SHELXL97* (Sheldrick, 2008 ▸), *SHELXT2014/4* (Sheldrick, 2015*a* ▸; Ruf & Noll, 2014 ▸), *SHELXL2014/7* (Sheldrick, 2015*b* ▸), *ORTEP-3 for Windows* (Farrugia, 2012 ▸), *Mercury* (Macrae *et al.*, 2008 ▸) and *publCIF* (Westrip, 2010 ▸).

## supplementary crystallographic information

### (Cycloocta-1,5-diene)(1,1,3,3,5,5,7,7-octaphenyl-1,7-diphospha-3,5-diphosphoniaheptan-4-yl)iridium(I) bis(trifluoromethanesulfonate)–ethyl acetate–dichloromethane (1/1/1) (1b) Crystal data

**Table:**

[Ir(C_51_H_45_P_4_)(C_8_H_12_)](CF_3_SO_3_)_2_·CH_3_CO_2_C_2_H_5_·CH_2_Cl_2_	*Z* = 2
*M**_r_* = 1553.30	*F*(000) = 1568
Triclinic, *P*1	*D*_x_ = 1.576 Mg m^−^^3^
*a* = 13.3105 (2) Å	Mo *K*α radiation, λ = 0.71073 Å
*b* = 14.3109 (3) Å	Cell parameters from 37798 reflections
*c* = 19.8482 (3) Å	θ = 1.0–25.0°
α = 68.949 (1)°	µ = 2.35 mm^−^^1^
β = 74.426 (1)°	*T* = 233 K
γ = 70.256 (1)°	Prism, colorless
*V* = 3274.1 (1) Å^3^	0.21 × 0.10 × 0.06 mm

### (Cycloocta-1,5-diene)(1,1,3,3,5,5,7,7-octaphenyl-1,7-diphospha-3,5-diphosphoniaheptan-4-yl)iridium(I) bis(trifluoromethanesulfonate)–ethyl acetate–dichloromethane (1/1/1) (1b) Data collection

**Table:**

Nonius KappaCCD diffractometer	10195 reflections with *I* > 2σ(*I*)
Radiation source: fine-focus sealed tube	*R*_int_ = 0.027
Graphite monochromator	θ_max_ = 24.8°, θ_min_ = 1.6°
phi– and ω–scans	*h* = −15→15
20473 measured reflections	*k* = −16→16
11235 independent reflections	*l* = −23→23

### (Cycloocta-1,5-diene)(1,1,3,3,5,5,7,7-octaphenyl-1,7-diphospha-3,5-diphosphoniaheptan-4-yl)iridium(I) bis(trifluoromethanesulfonate)–ethyl acetate–dichloromethane (1/1/1) (1b) Refinement

**Table:**

Refinement on *F*^2^	Primary atom site location: structure-invariant direct methods
Least-squares matrix: full	Secondary atom site location: difference Fourier map
*R*[*F*^2^ > 2σ(*F*^2^)] = 0.025	Hydrogen site location: inferred from neighbouring sites
*wR*(*F*^2^) = 0.055	H atoms treated by a mixture of independent and constrained refinement
*S* = 1.03	*w* = 1/[σ^2^(*F*_o_^2^) + (0.0149*P*)^2^ + 2.9778*P*] where *P* = (*F*_o_^2^ + 2*F*_c_^2^)/3
11235 reflections	(Δ/σ)_max_ = 0.003
976 parameters	Δρ_max_ = 0.58 e Å^−^^3^
5 restraints	Δρ_min_ = −0.68 e Å^−^^3^

### (Cycloocta-1,5-diene)(1,1,3,3,5,5,7,7-octaphenyl-1,7-diphospha-3,5-diphosphoniaheptan-4-yl)iridium(I) bis(trifluoromethanesulfonate)–ethyl acetate–dichloromethane (1/1/1) (1b) Special details

**Table:**

Geometry. All esds (except the esd in the dihedral angle between two l.s. planes) are estimated using the full covariance matrix. The cell esds are taken into account individually in the estimation of esds in distances, angles and torsion angles; correlations between esds in cell parameters are only used when they are defined by crystal symmetry. An approximate (isotropic) treatment of cell esds is used for estimating esds involving l.s. planes.
Refinement. Hydrogen atoms at C1 and C4=C5, C8=C9 were found and refined with bond restraints (*d* = 0.96 for H1 and d=0.93 for H4, H5, H8 and H9). Both triflate-anions show positional disorder in ratio 0.7:0.3. The solvent molecule CH2Cl2 shows also a positional disorder of this ratio, hydrogen atoms of this disordered molecule were omitted.

### (Cycloocta-1,5-diene)(1,1,3,3,5,5,7,7-octaphenyl-1,7-diphospha-3,5-diphosphoniaheptan-4-yl)iridium(I) bis(trifluoromethanesulfonate)–ethyl acetate–dichloromethane (1/1/1) (1b) Fractional atomic coordinates and isotropic or equivalent isotropic displacement parameters (Å^2^)

**Table:**

	*x*	*y*	*z*	*U*_iso_*/*U*_eq_	Occ. (<1)
Ir1	0.461480 (9)	0.703450 (8)	0.132966 (5)	0.02536 (4)	
P1	0.58314 (6)	0.65692 (6)	0.21612 (4)	0.02768 (16)	
P2	0.38022 (6)	0.76427 (5)	0.29095 (4)	0.02648 (16)	
P3	0.22546 (6)	0.69359 (6)	0.24242 (4)	0.02697 (16)	
P4	0.40431 (6)	0.55141 (5)	0.17784 (4)	0.02696 (16)	
C1	0.3280 (2)	0.7630 (2)	0.21544 (14)	0.0258 (6)	
H1	0.2936 (18)	0.8334 (13)	0.1955 (12)	0.011 (6)*	
C2	0.4957 (2)	0.6538 (2)	0.30570 (14)	0.0276 (6)	
H2A	0.4729	0.5894	0.3264	0.033*	
H2B	0.5348	0.6571	0.3398	0.033*	
C3	0.3009 (2)	0.5595 (2)	0.26020 (14)	0.0274 (6)	
H3A	0.2533	0.5156	0.2686	0.033*	
H3B	0.3351	0.5369	0.3035	0.033*	
C4	0.3868 (3)	0.8415 (2)	0.05239 (16)	0.0360 (7)	
H4	0.3186 (17)	0.877 (2)	0.0726 (14)	0.034 (8)*	
C5	0.4777 (3)	0.8572 (2)	0.06626 (15)	0.0343 (7)	
H5	0.460 (2)	0.9001 (19)	0.0958 (14)	0.034 (8)*	
C6	0.5813 (3)	0.8596 (2)	0.00956 (16)	0.0406 (8)	
H6A	0.6143	0.9088	0.0133	0.049*	
H6B	0.5626	0.8845	−0.0396	0.049*	
C7	0.6643 (3)	0.7521 (2)	0.01956 (16)	0.0395 (7)	
H7A	0.7061	0.7466	−0.0284	0.047*	
H7B	0.7148	0.7456	0.0500	0.047*	
C8	0.6107 (2)	0.6642 (2)	0.05499 (15)	0.0317 (7)	
H8	0.653 (2)	0.5994 (16)	0.0787 (13)	0.028 (7)*	
C9	0.5245 (2)	0.6586 (2)	0.03001 (15)	0.0322 (7)	
H9	0.518 (2)	0.5915 (15)	0.0382 (15)	0.033 (8)*	
C10	0.4799 (3)	0.7379 (3)	−0.03775 (16)	0.0428 (8)	
H10A	0.4547	0.7029	−0.0617	0.051*	
H10B	0.5383	0.7657	−0.0723	0.051*	
C11	0.3865 (3)	0.8281 (3)	−0.02031 (16)	0.0448 (8)	
H11A	0.3920	0.8924	−0.0596	0.054*	
H11B	0.3177	0.8155	−0.0185	0.054*	
C14	0.0575 (3)	0.3930 (3)	0.33642 (19)	0.0538 (9)	
C15	−0.0499 (3)	0.4683 (3)	0.3472 (2)	0.0653 (11)	
H15A	−0.0409	0.5264	0.3571	0.098*	
H15B	−0.0969	0.4342	0.3883	0.098*	
H15C	−0.0819	0.4934	0.3033	0.098*	
C16	0.1526 (4)	0.2208 (4)	0.3366 (3)	0.1033 (18)	
H16A	0.1824	0.2376	0.2842	0.124*	
H16B	0.2055	0.2203	0.3629	0.124*	
C17	0.1323 (6)	0.1214 (4)	0.3615 (4)	0.149 (3)	
H17A	0.1993	0.0694	0.3527	0.223*	
H17B	0.0801	0.1223	0.3351	0.223*	
H17C	0.1037	0.1048	0.4135	0.223*	
C101	0.6851 (2)	0.5311 (2)	0.23307 (16)	0.0330 (7)	
C102	0.7811 (3)	0.5228 (3)	0.18279 (18)	0.0443 (8)	
H102	0.7913	0.5805	0.1424	0.053*	
C103	0.8621 (3)	0.4293 (3)	0.1921 (2)	0.0570 (10)	
H103	0.9265	0.4237	0.1579	0.068*	
C104	0.8474 (3)	0.3453 (3)	0.2515 (2)	0.0603 (11)	
H104	0.9021	0.2823	0.2579	0.072*	
C105	0.7533 (3)	0.3532 (3)	0.3013 (2)	0.0527 (9)	
H105	0.7441	0.2953	0.3418	0.063*	
C106	0.6716 (3)	0.4455 (2)	0.29282 (17)	0.0389 (7)	
H106	0.6073	0.4501	0.3273	0.047*	
C107	0.6667 (2)	0.7374 (2)	0.21511 (15)	0.0320 (7)	
C108	0.6685 (3)	0.8334 (2)	0.16393 (17)	0.0411 (8)	
H108	0.6261	0.8597	0.1268	0.049*	
C109	0.7319 (3)	0.8909 (3)	0.1668 (2)	0.0525 (9)	
H109	0.7332	0.9552	0.1311	0.063*	
C110	0.7929 (3)	0.8543 (3)	0.2214 (2)	0.0515 (9)	
H110	0.8359	0.8934	0.2234	0.062*	
C111	0.7905 (3)	0.7596 (3)	0.27351 (19)	0.0454 (8)	
H111	0.8314	0.7347	0.3114	0.054*	
C112	0.7289 (2)	0.7014 (2)	0.27038 (17)	0.0385 (7)	
H112	0.7288	0.6367	0.3058	0.046*	
C201	0.2931 (2)	0.7600 (2)	0.37806 (15)	0.0300 (6)	
C202	0.2019 (3)	0.8435 (2)	0.38288 (17)	0.0385 (7)	
H202	0.1852	0.8990	0.3408	0.046*	
C203	0.1363 (3)	0.8439 (3)	0.44981 (19)	0.0483 (9)	
H203	0.0740	0.8992	0.4533	0.058*	
C204	0.1627 (3)	0.7629 (3)	0.51158 (18)	0.0524 (9)	
H204	0.1181	0.7636	0.5571	0.063*	
C205	0.2527 (3)	0.6815 (3)	0.50748 (17)	0.0499 (9)	
H205	0.2698	0.6271	0.5501	0.060*	
C206	0.3186 (2)	0.6793 (2)	0.44076 (16)	0.0387 (7)	
H206	0.3804	0.6234	0.4379	0.046*	
C207	0.4181 (2)	0.8828 (2)	0.26773 (15)	0.0320 (7)	
C208	0.3747 (3)	0.9729 (2)	0.21576 (16)	0.0369 (7)	
H208	0.3309	0.9712	0.1864	0.044*	
C209	0.3955 (3)	1.0651 (3)	0.20704 (19)	0.0503 (9)	
H209	0.3651	1.1261	0.1722	0.060*	
C210	0.4602 (4)	1.0679 (3)	0.2489 (2)	0.0648 (11)	
H210	0.4751	1.1307	0.2421	0.078*	
C211	0.5038 (3)	0.9792 (3)	0.3011 (2)	0.0663 (11)	
H211	0.5484	0.9819	0.3296	0.080*	
C212	0.4823 (3)	0.8863 (3)	0.31180 (19)	0.0476 (9)	
H212	0.5105	0.8262	0.3482	0.057*	
C301	0.1215 (2)	0.7077 (2)	0.32096 (16)	0.0335 (7)	
C302	0.0369 (3)	0.7986 (3)	0.31247 (19)	0.0473 (9)	
H302	0.0372	0.8519	0.2677	0.057*	
C303	−0.0468 (3)	0.8103 (4)	0.3697 (2)	0.0657 (12)	
H303	−0.1038	0.8716	0.3641	0.079*	
C304	−0.0473 (3)	0.7322 (4)	0.4349 (3)	0.0755 (14)	
H304	−0.1045	0.7404	0.4740	0.091*	
C305	0.0349 (4)	0.6427 (4)	0.4434 (2)	0.0693 (12)	
H305	0.0333	0.5895	0.4881	0.083*	
C306	0.1207 (3)	0.6295 (3)	0.38662 (17)	0.0471 (8)	
H306	0.1776	0.5681	0.3928	0.056*	
C307	0.1454 (2)	0.7273 (2)	0.17256 (15)	0.0326 (7)	
C308	0.1260 (3)	0.8232 (3)	0.11981 (17)	0.0440 (8)	
H308	0.1604	0.8731	0.1156	0.053*	
C309	0.0538 (3)	0.8442 (3)	0.0727 (2)	0.0584 (10)	
H309	0.0417	0.9082	0.0357	0.070*	
C310	0.0012 (3)	0.7740 (4)	0.0796 (2)	0.0623 (11)	
H310	−0.0465	0.7895	0.0473	0.075*	
C311	0.0176 (3)	0.6799 (3)	0.1338 (2)	0.0537 (10)	
H311	−0.0202	0.6320	0.1393	0.064*	
C312	0.0897 (2)	0.6567 (3)	0.17995 (17)	0.0406 (8)	
H312	0.1014	0.5924	0.2167	0.049*	
C401	0.4968 (2)	0.4226 (2)	0.20953 (15)	0.0295 (6)	
C402	0.5886 (2)	0.3912 (2)	0.16092 (16)	0.0362 (7)	
H402	0.6045	0.4377	0.1146	0.043*	
C403	0.6568 (3)	0.2914 (3)	0.18040 (19)	0.0461 (8)	
H403	0.7184	0.2707	0.1471	0.055*	
C404	0.6349 (3)	0.2227 (2)	0.24823 (19)	0.0452 (8)	
H404	0.6813	0.1552	0.2611	0.054*	
C405	0.5449 (3)	0.2529 (2)	0.29694 (17)	0.0439 (8)	
H405	0.5303	0.2063	0.3434	0.053*	
C406	0.4753 (3)	0.3523 (2)	0.27784 (16)	0.0375 (7)	
H406	0.4133	0.3720	0.3112	0.045*	
C407	0.3319 (2)	0.5201 (2)	0.12470 (15)	0.0320 (7)	
C408	0.2978 (2)	0.5915 (2)	0.06077 (16)	0.0381 (7)	
H408	0.3170	0.6546	0.0416	0.046*	
C409	0.2356 (3)	0.5705 (3)	0.02483 (17)	0.0476 (9)	
H409	0.2132	0.6192	−0.0186	0.057*	
C410	0.2065 (3)	0.4788 (3)	0.05248 (19)	0.0503 (9)	
H410	0.1642	0.4651	0.0280	0.060*	
C411	0.2393 (3)	0.4071 (3)	0.1159 (2)	0.0496 (9)	
H411	0.2184	0.3447	0.1351	0.060*	
C412	0.3030 (3)	0.4268 (2)	0.15140 (17)	0.0407 (8)	
H412	0.3270	0.3767	0.1939	0.049*	
O7	0.1411 (3)	0.4140 (2)	0.3173 (2)	0.1064 (12)	
O8	0.0513 (2)	0.2987 (2)	0.34964 (17)	0.0749 (8)	
S1	0.1125 (3)	1.0951 (3)	0.13842 (18)	0.0560 (7)	0.70
C12	−0.0306 (4)	1.1228 (4)	0.1465 (3)	0.0754 (13)	0.70
O1	0.1527 (6)	1.0904 (6)	0.0623 (4)	0.0901 (19)	0.70
O2	0.1282 (14)	1.1797 (10)	0.1514 (9)	0.105 (4)	0.70
O3	0.1374 (11)	0.9978 (7)	0.1880 (5)	0.069 (2)	0.70
F1	−0.0869 (5)	1.1246 (5)	0.2118 (3)	0.0854 (16)	0.70
F2	−0.0532 (8)	1.0620 (10)	0.1224 (7)	0.132 (4)	0.70
F3	−0.0644 (9)	1.2209 (11)	0.1072 (7)	0.140 (5)	0.70
S2	0.57325 (12)	0.62722 (10)	0.51933 (7)	0.0420 (3)	0.70
C13	0.6889 (7)	0.6786 (6)	0.4978 (3)	0.0565 (15)	0.70
O4	0.5883 (3)	0.5956 (2)	0.45537 (14)	0.0747 (8)	
O5	0.5977 (2)	0.54564 (19)	0.58397 (12)	0.0607 (7)	
O6	0.4829 (5)	0.7109 (5)	0.5289 (4)	0.0915 (18)	0.70
F4	0.7006 (9)	0.7076 (10)	0.5518 (8)	0.117 (5)	0.70
F5	0.6855 (9)	0.7612 (7)	0.4389 (7)	0.092 (3)	0.70
F6	0.7765 (8)	0.6095 (9)	0.4838 (6)	0.121 (4)	0.70
S1A	0.1059 (11)	1.0991 (13)	0.1635 (9)	0.151 (6)	0.30
C12A	−0.0306 (4)	1.1228 (4)	0.1465 (3)	0.0754 (13)	0.30
O1A	0.143 (3)	1.104 (3)	0.0811 (16)	0.25 (2)	0.30
O2A	0.115 (3)	1.180 (3)	0.176 (2)	0.125 (15)	0.30
O3A	0.134 (3)	0.992 (2)	0.2148 (13)	0.090 (9)	0.30
F1A	−0.056 (2)	1.096 (2)	0.2263 (17)	0.30 (2)	0.30
F2A	−0.0567 (11)	1.0479 (18)	0.1356 (14)	0.093 (8)	0.30
F3A	−0.0797 (14)	1.212 (2)	0.0996 (10)	0.076 (5)	0.30
S2A	0.6515 (4)	0.5980 (3)	0.50435 (17)	0.0555 (8)	0.30
C13A	0.6234 (14)	0.7302 (14)	0.4998 (8)	0.063 (4)	0.30
O6A	0.778 (2)	0.573 (2)	0.4828 (13)	0.092 (9)	0.30
F4A	0.656 (2)	0.7358 (16)	0.5501 (15)	0.080 (6)	0.30
F5A	0.649 (2)	0.7896 (19)	0.4384 (18)	0.099 (8)	0.30
F6A	0.5153 (11)	0.7693 (10)	0.5148 (8)	0.120 (5)	0.30
C18	0.7007 (8)	1.0799 (7)	0.4747 (7)	0.096 (3)	0.70
Cl1	0.8229 (3)	1.0644 (2)	0.40662 (18)	0.1241 (11)	0.70
Cl2	0.6250 (13)	1.0045 (8)	0.4790 (9)	0.158 (5)	0.35
Cl2B	0.628 (3)	1.012 (3)	0.5128 (18)	0.64 (3)	0.35
C18A	0.662 (3)	0.985 (3)	0.447 (3)	0.112 (17)	0.30
Cl1A	0.7639 (17)	1.0444 (13)	0.4296 (9)	0.294 (11)	0.30
Cl2A	0.6556 (10)	0.9510 (10)	0.5190 (10)	0.167 (5)	0.30

### (Cycloocta-1,5-diene)(1,1,3,3,5,5,7,7-octaphenyl-1,7-diphospha-3,5-diphosphoniaheptan-4-yl)iridium(I) bis(trifluoromethanesulfonate)–ethyl acetate–dichloromethane (1/1/1) (1b) Atomic displacement parameters (Å^2^)

**Table:**

	*U*^11^	*U*^22^	*U*^33^	*U*^12^	*U*^13^	*U*^23^
Ir1	0.02827 (7)	0.02462 (6)	0.02257 (6)	−0.00713 (5)	−0.00395 (4)	−0.00646 (4)
P1	0.0271 (4)	0.0282 (4)	0.0268 (4)	−0.0073 (3)	−0.0048 (3)	−0.0071 (3)
P2	0.0282 (4)	0.0277 (4)	0.0248 (4)	−0.0072 (3)	−0.0049 (3)	−0.0089 (3)
P3	0.0252 (4)	0.0288 (4)	0.0282 (4)	−0.0059 (3)	−0.0049 (3)	−0.0109 (3)
P4	0.0281 (4)	0.0266 (4)	0.0266 (4)	−0.0069 (3)	−0.0042 (3)	−0.0091 (3)
C1	0.0283 (15)	0.0221 (14)	0.0266 (14)	−0.0044 (12)	−0.0085 (12)	−0.0061 (12)
C2	0.0281 (15)	0.0294 (15)	0.0242 (14)	−0.0089 (12)	−0.0054 (12)	−0.0046 (12)
C3	0.0290 (15)	0.0258 (15)	0.0294 (15)	−0.0087 (12)	−0.0048 (12)	−0.0094 (12)
C4	0.0359 (17)	0.0325 (17)	0.0296 (16)	−0.0010 (14)	−0.0070 (14)	−0.0040 (13)
C5	0.0477 (19)	0.0225 (15)	0.0263 (15)	−0.0078 (14)	−0.0042 (14)	−0.0029 (12)
C6	0.055 (2)	0.0338 (17)	0.0302 (16)	−0.0198 (15)	−0.0012 (14)	−0.0024 (13)
C7	0.0406 (18)	0.0468 (19)	0.0320 (16)	−0.0220 (15)	0.0082 (14)	−0.0136 (14)
C8	0.0340 (16)	0.0301 (16)	0.0268 (15)	−0.0061 (14)	0.0008 (13)	−0.0102 (13)
C9	0.0385 (17)	0.0334 (17)	0.0229 (14)	−0.0111 (14)	0.0030 (12)	−0.0110 (13)
C10	0.054 (2)	0.048 (2)	0.0288 (16)	−0.0203 (17)	−0.0058 (15)	−0.0099 (14)
C11	0.057 (2)	0.0425 (19)	0.0313 (17)	−0.0099 (17)	−0.0172 (15)	−0.0023 (14)
C14	0.059 (2)	0.052 (2)	0.049 (2)	−0.029 (2)	−0.0048 (18)	−0.0037 (17)
C15	0.068 (3)	0.056 (2)	0.072 (3)	−0.020 (2)	−0.007 (2)	−0.019 (2)
C16	0.078 (3)	0.058 (3)	0.149 (5)	−0.012 (3)	0.008 (3)	−0.027 (3)
C17	0.145 (6)	0.061 (4)	0.210 (8)	−0.017 (4)	0.005 (5)	−0.043 (4)
C101	0.0304 (16)	0.0344 (17)	0.0390 (17)	−0.0049 (13)	−0.0131 (13)	−0.0146 (14)
C102	0.0359 (18)	0.047 (2)	0.050 (2)	−0.0052 (16)	−0.0096 (15)	−0.0186 (16)
C103	0.0337 (19)	0.063 (3)	0.080 (3)	0.0042 (18)	−0.0109 (18)	−0.043 (2)
C104	0.059 (3)	0.042 (2)	0.089 (3)	0.0125 (19)	−0.040 (2)	−0.034 (2)
C105	0.067 (3)	0.0365 (19)	0.061 (2)	−0.0021 (18)	−0.035 (2)	−0.0143 (17)
C106	0.0465 (19)	0.0314 (17)	0.0445 (18)	−0.0091 (15)	−0.0167 (15)	−0.0121 (14)
C107	0.0274 (15)	0.0349 (16)	0.0327 (15)	−0.0081 (13)	−0.0015 (12)	−0.0115 (13)
C108	0.0413 (18)	0.0406 (18)	0.0426 (18)	−0.0137 (15)	−0.0095 (15)	−0.0093 (15)
C109	0.059 (2)	0.043 (2)	0.059 (2)	−0.0250 (18)	−0.0132 (19)	−0.0064 (17)
C110	0.043 (2)	0.055 (2)	0.068 (2)	−0.0266 (17)	−0.0073 (18)	−0.0201 (19)
C111	0.0376 (18)	0.055 (2)	0.054 (2)	−0.0180 (16)	−0.0123 (16)	−0.0195 (17)
C112	0.0360 (17)	0.0399 (18)	0.0403 (17)	−0.0115 (15)	−0.0101 (14)	−0.0086 (14)
C201	0.0320 (16)	0.0365 (16)	0.0279 (15)	−0.0136 (13)	−0.0020 (12)	−0.0151 (13)
C202	0.0393 (18)	0.0393 (18)	0.0407 (18)	−0.0105 (15)	−0.0059 (14)	−0.0171 (14)
C203	0.0413 (19)	0.055 (2)	0.052 (2)	−0.0096 (17)	0.0026 (16)	−0.0307 (18)
C204	0.050 (2)	0.075 (3)	0.0373 (19)	−0.024 (2)	0.0089 (16)	−0.0285 (19)
C205	0.051 (2)	0.066 (2)	0.0297 (17)	−0.0202 (19)	−0.0019 (15)	−0.0103 (16)
C206	0.0384 (17)	0.0453 (19)	0.0313 (16)	−0.0111 (15)	−0.0053 (14)	−0.0105 (14)
C207	0.0328 (16)	0.0313 (16)	0.0350 (16)	−0.0104 (13)	0.0007 (13)	−0.0166 (13)
C208	0.0423 (18)	0.0321 (17)	0.0360 (17)	−0.0099 (14)	−0.0037 (14)	−0.0119 (14)
C209	0.064 (2)	0.0371 (19)	0.050 (2)	−0.0210 (17)	0.0004 (18)	−0.0131 (16)
C210	0.078 (3)	0.051 (2)	0.083 (3)	−0.034 (2)	−0.003 (2)	−0.031 (2)
C211	0.068 (3)	0.073 (3)	0.085 (3)	−0.028 (2)	−0.018 (2)	−0.043 (3)
C212	0.052 (2)	0.050 (2)	0.055 (2)	−0.0148 (17)	−0.0172 (17)	−0.0250 (17)
C301	0.0256 (15)	0.0472 (18)	0.0356 (16)	−0.0130 (14)	−0.0016 (13)	−0.0211 (14)
C302	0.0328 (18)	0.062 (2)	0.056 (2)	−0.0031 (17)	−0.0111 (16)	−0.0344 (18)
C303	0.0309 (19)	0.104 (3)	0.080 (3)	−0.002 (2)	−0.0041 (19)	−0.067 (3)
C304	0.049 (2)	0.133 (4)	0.069 (3)	−0.039 (3)	0.021 (2)	−0.067 (3)
C305	0.073 (3)	0.104 (4)	0.044 (2)	−0.050 (3)	0.016 (2)	−0.031 (2)
C306	0.049 (2)	0.058 (2)	0.0400 (19)	−0.0244 (18)	0.0026 (16)	−0.0193 (17)
C307	0.0263 (15)	0.0393 (17)	0.0343 (16)	−0.0030 (13)	−0.0064 (13)	−0.0178 (14)
C308	0.0423 (19)	0.045 (2)	0.0428 (19)	−0.0051 (16)	−0.0157 (15)	−0.0104 (16)
C309	0.055 (2)	0.061 (2)	0.050 (2)	0.003 (2)	−0.0254 (19)	−0.0108 (19)
C310	0.043 (2)	0.090 (3)	0.062 (2)	0.000 (2)	−0.0249 (19)	−0.037 (2)
C311	0.0348 (19)	0.075 (3)	0.068 (2)	−0.0120 (18)	−0.0126 (17)	−0.041 (2)
C312	0.0311 (17)	0.048 (2)	0.0478 (19)	−0.0099 (15)	−0.0077 (15)	−0.0204 (16)
C401	0.0307 (15)	0.0285 (15)	0.0352 (16)	−0.0073 (13)	−0.0096 (13)	−0.0137 (13)
C402	0.0335 (17)	0.0369 (17)	0.0385 (17)	−0.0068 (14)	−0.0058 (14)	−0.0141 (14)
C403	0.0350 (18)	0.047 (2)	0.056 (2)	0.0012 (16)	−0.0064 (16)	−0.0260 (17)
C404	0.048 (2)	0.0313 (17)	0.058 (2)	−0.0021 (15)	−0.0201 (17)	−0.0141 (16)
C405	0.057 (2)	0.0310 (17)	0.0424 (18)	−0.0111 (16)	−0.0154 (17)	−0.0050 (14)
C406	0.0423 (18)	0.0328 (17)	0.0374 (17)	−0.0084 (14)	−0.0079 (14)	−0.0112 (14)
C407	0.0287 (15)	0.0376 (17)	0.0320 (16)	−0.0058 (13)	−0.0047 (13)	−0.0162 (13)
C408	0.0339 (17)	0.0446 (18)	0.0357 (17)	−0.0102 (14)	−0.0021 (14)	−0.0148 (15)
C409	0.0374 (18)	0.074 (3)	0.0343 (17)	−0.0092 (18)	−0.0093 (15)	−0.0219 (17)
C410	0.0394 (19)	0.076 (3)	0.052 (2)	−0.0160 (19)	−0.0065 (16)	−0.039 (2)
C411	0.048 (2)	0.054 (2)	0.062 (2)	−0.0187 (17)	−0.0065 (18)	−0.0327 (19)
C412	0.0442 (19)	0.0394 (18)	0.0459 (19)	−0.0115 (15)	−0.0095 (15)	−0.0192 (15)
O7	0.069 (2)	0.068 (2)	0.177 (4)	−0.0401 (18)	0.000 (2)	−0.026 (2)
O8	0.0601 (18)	0.0499 (16)	0.110 (2)	−0.0234 (14)	0.0019 (16)	−0.0216 (16)
S1	0.0412 (10)	0.0431 (13)	0.0814 (13)	−0.0096 (9)	−0.0161 (9)	−0.0130 (10)
C12	0.068 (3)	0.072 (3)	0.081 (3)	0.005 (3)	−0.022 (3)	−0.033 (3)
O1	0.067 (3)	0.102 (4)	0.081 (4)	−0.012 (3)	0.021 (3)	−0.038 (4)
O2	0.134 (7)	0.060 (5)	0.137 (8)	−0.048 (5)	−0.023 (5)	−0.025 (5)
O3	0.074 (4)	0.037 (4)	0.094 (7)	0.000 (3)	−0.055 (5)	−0.002 (4)
F1	0.062 (2)	0.102 (4)	0.074 (3)	−0.003 (3)	0.014 (2)	−0.041 (3)
F2	0.118 (7)	0.155 (9)	0.164 (7)	−0.005 (5)	−0.073 (5)	−0.089 (6)
F3	0.126 (8)	0.093 (6)	0.169 (8)	0.040 (5)	−0.079 (5)	−0.028 (5)
S2	0.0382 (7)	0.0489 (8)	0.0380 (7)	−0.0139 (6)	−0.0080 (6)	−0.0089 (6)
C13	0.061 (4)	0.057 (4)	0.050 (4)	−0.028 (4)	−0.010 (3)	−0.002 (3)
O4	0.097 (2)	0.095 (2)	0.0479 (15)	−0.0380 (18)	−0.0246 (15)	−0.0190 (15)
O5	0.093 (2)	0.0556 (15)	0.0385 (13)	−0.0322 (14)	−0.0133 (13)	−0.0069 (12)
O6	0.056 (3)	0.083 (4)	0.121 (5)	0.008 (3)	−0.020 (3)	−0.036 (4)
F4	0.139 (9)	0.195 (10)	0.072 (4)	−0.118 (8)	−0.018 (5)	−0.034 (6)
F5	0.121 (6)	0.085 (7)	0.062 (4)	−0.067 (6)	0.007 (4)	0.006 (5)
F6	0.049 (5)	0.141 (9)	0.162 (6)	−0.022 (6)	−0.003 (4)	−0.044 (5)
S1A	0.116 (8)	0.080 (6)	0.291 (18)	0.021 (5)	−0.121 (10)	−0.074 (10)
C12A	0.068 (3)	0.072 (3)	0.081 (3)	0.005 (3)	−0.022 (3)	−0.033 (3)
O1A	0.28 (3)	0.35 (4)	0.15 (2)	−0.16 (3)	0.15 (2)	−0.18 (2)
O2A	0.111 (18)	0.095 (16)	0.22 (4)	0.003 (13)	−0.10 (2)	−0.085 (19)
O3A	0.089 (11)	0.088 (12)	0.078 (15)	−0.032 (8)	−0.066 (12)	0.037 (10)
F1A	0.31 (4)	0.126 (16)	0.21 (2)	0.028 (18)	0.13 (2)	0.027 (14)
F2A	0.021 (6)	0.088 (10)	0.187 (18)	−0.029 (7)	0.014 (8)	−0.069 (10)
F3A	0.026 (4)	0.092 (12)	0.058 (6)	0.018 (5)	−0.012 (5)	0.010 (6)
S2A	0.068 (3)	0.060 (2)	0.0417 (17)	−0.026 (2)	−0.0116 (17)	−0.0085 (15)
C13A	0.062 (11)	0.070 (11)	0.047 (9)	0.001 (9)	−0.016 (8)	−0.020 (9)
O6A	0.080 (15)	0.088 (14)	0.063 (9)	0.039 (10)	−0.006 (8)	−0.032 (8)
F4A	0.134 (18)	0.086 (8)	0.055 (7)	−0.088 (11)	−0.018 (10)	−0.006 (6)
F5A	0.17 (2)	0.053 (9)	0.058 (8)	−0.019 (10)	−0.023 (11)	−0.006 (7)
F6A	0.078 (9)	0.093 (9)	0.137 (10)	0.021 (7)	0.003 (7)	−0.030 (9)
C18	0.084 (6)	0.076 (5)	0.147 (9)	−0.005 (4)	−0.037 (6)	−0.057 (6)
Cl1	0.178 (3)	0.0668 (14)	0.129 (2)	−0.0061 (17)	−0.036 (2)	−0.0482 (14)
Cl2	0.244 (15)	0.098 (6)	0.186 (10)	−0.107 (8)	−0.096 (10)	−0.005 (6)
Cl2B	0.68 (5)	0.54 (5)	0.42 (3)	0.02 (4)	0.24 (4)	−0.23 (3)
C18A	0.078 (14)	0.083 (19)	0.22 (5)	−0.030 (11)	−0.01 (2)	−0.10 (3)
Cl1A	0.44 (3)	0.223 (13)	0.244 (15)	−0.188 (15)	0.071 (15)	−0.095 (11)
Cl2A	0.166 (8)	0.112 (7)	0.263 (15)	−0.034 (6)	−0.073 (10)	−0.077 (8)

### (Cycloocta-1,5-diene)(1,1,3,3,5,5,7,7-octaphenyl-1,7-diphospha-3,5-diphosphoniaheptan-4-yl)iridium(I) bis(trifluoromethanesulfonate)–ethyl acetate–dichloromethane (1/1/1) (1b) Geometric parameters (Å, º)

**Table:**

Ir1—C4	2.169 (3)	C207—C212	1.400 (4)
Ir1—C5	2.172 (3)	C208—C209	1.381 (5)
Ir1—C8	2.208 (3)	C208—H208	0.9400
Ir1—C9	2.225 (3)	C209—C210	1.366 (5)
Ir1—C1	2.232 (3)	C209—H209	0.9400
Ir1—P4	2.3386 (7)	C210—C211	1.379 (6)
Ir1—P1	2.3889 (7)	C210—H210	0.9400
P1—C101	1.831 (3)	C211—C212	1.385 (5)
P1—C2	1.840 (3)	C211—H211	0.9400
P1—C107	1.844 (3)	C212—H212	0.9400
P2—C2	1.794 (3)	C301—C306	1.379 (4)
P2—C201	1.798 (3)	C301—C302	1.394 (4)
P2—C207	1.799 (3)	C302—C303	1.375 (5)
P2—C1	1.821 (3)	C302—H302	0.9400
P3—C3	1.793 (3)	C303—C304	1.374 (6)
P3—C307	1.804 (3)	C303—H303	0.9400
P3—C301	1.807 (3)	C304—C305	1.364 (6)
P3—C1	1.811 (3)	C304—H304	0.9400
P4—C401	1.833 (3)	C305—C306	1.388 (5)
P4—C3	1.844 (3)	C305—H305	0.9400
P4—C407	1.849 (3)	C306—H306	0.9400
C1—H1	0.938 (16)	C307—C308	1.385 (4)
C2—H2A	0.9800	C307—C312	1.391 (4)
C2—H2B	0.9800	C308—C309	1.404 (5)
C3—H3A	0.9800	C308—H308	0.9400
C3—H3B	0.9800	C309—C310	1.359 (6)
C4—C5	1.414 (4)	C309—H309	0.9400
C4—C11	1.524 (4)	C310—C311	1.380 (6)
C4—H4	0.939 (17)	C310—H310	0.9400
C5—C6	1.529 (4)	C311—C312	1.382 (4)
C5—H5	0.928 (17)	C311—H311	0.9400
C6—C7	1.541 (4)	C312—H312	0.9400
C6—H6A	0.9800	C401—C402	1.390 (4)
C6—H6B	0.9800	C401—C406	1.393 (4)
C7—C8	1.512 (4)	C402—C403	1.389 (4)
C7—H7A	0.9800	C402—H402	0.9400
C7—H7B	0.9800	C403—C404	1.378 (5)
C8—C9	1.402 (4)	C403—H403	0.9400
C8—H8	0.935 (17)	C404—C405	1.373 (5)
C9—C10	1.525 (4)	C404—H404	0.9400
C9—H9	0.946 (17)	C405—C406	1.391 (4)
C10—C11	1.528 (5)	C405—H405	0.9400
C10—H10A	0.9800	C406—H406	0.9400
C10—H10B	0.9800	C407—C408	1.387 (4)
C11—H11A	0.9800	C407—C412	1.392 (4)
C11—H11B	0.9800	C408—C409	1.387 (4)
C14—O7	1.179 (4)	C408—H408	0.9400
C14—O8	1.306 (4)	C409—C410	1.372 (5)
C14—C15	1.487 (5)	C409—H409	0.9400
C15—H15A	0.9700	C410—C411	1.375 (5)
C15—H15B	0.9700	C410—H410	0.9400
C15—H15C	0.9700	C411—C412	1.387 (4)
C16—C17	1.421 (8)	C411—H411	0.9400
C16—O8	1.461 (5)	C412—H412	0.9400
C16—H16A	0.9800	S1—O3	1.377 (10)
C16—H16B	0.9800	S1—O2	1.414 (15)
C17—H17A	0.9700	S1—O1	1.479 (8)
C17—H17B	0.9700	S1—C12	1.785 (6)
C17—H17C	0.9700	C12—F2	1.277 (12)
C101—C106	1.390 (4)	C12—F1	1.315 (8)
C101—C102	1.394 (4)	C12—F3	1.325 (13)
C102—C103	1.392 (5)	S2—O6	1.408 (6)
C102—H102	0.9400	S2—O5	1.416 (3)
C103—C104	1.374 (6)	S2—O4	1.440 (3)
C103—H103	0.9400	S2—C13	1.811 (6)
C104—C105	1.371 (6)	C13—F6	1.287 (12)
C104—H104	0.9400	C13—F5	1.329 (12)
C105—C106	1.388 (4)	C13—F4	1.340 (15)
C105—H105	0.9400	S1A—O2A	1.32 (4)
C106—H106	0.9400	S1A—O3A	1.49 (3)
C107—C108	1.387 (4)	S1A—O1A	1.56 (3)
C107—C112	1.396 (4)	S2A—O6A	1.56 (3)
C108—C109	1.386 (5)	S2A—C13A	1.773 (18)
C108—H108	0.9400	C13A—F4A	1.23 (3)
C109—C110	1.374 (5)	C13A—F5A	1.25 (3)
C109—H109	0.9400	C13A—F6A	1.35 (2)
C110—C111	1.383 (5)	C18—Cl1A	1.182 (16)
C110—H110	0.9400	C18—Cl2B	1.47 (3)
C111—C112	1.378 (4)	C18—Cl2	1.676 (13)
C111—H111	0.9400	C18—Cl1	1.820 (13)
C112—H112	0.9400	C18—C18A	1.90 (3)
C201—C206	1.388 (4)	C18—Cl2A	1.958 (18)
C201—C202	1.397 (4)	Cl1—Cl1A	0.880 (17)
C202—C203	1.381 (4)	Cl2—Cl2B	0.73 (4)
C202—H202	0.9400	Cl2—C18A	0.78 (4)
C203—C204	1.381 (5)	Cl2—Cl2A	0.952 (14)
C203—H203	0.9400	Cl2—Cl1A	2.01 (2)
C204—C205	1.368 (5)	Cl2B—Cl2A	0.81 (4)
C204—H204	0.9400	Cl2B—C18A	1.42 (5)
C205—C206	1.383 (4)	Cl2B—Cl1A	2.15 (3)
C205—H205	0.9400	C18A—Cl2A	1.33 (5)
C206—H206	0.9400	C18A—Cl1A	1.74 (4)
C207—C208	1.386 (4)	Cl1A—Cl2A	2.29 (2)
			
C4—Ir1—C5	38.02 (12)	C207—C208—H208	119.9
C4—Ir1—C8	93.71 (11)	C210—C209—C208	120.3 (3)
C5—Ir1—C8	78.86 (11)	C210—C209—H209	119.9
C4—Ir1—C9	77.48 (11)	C208—C209—H209	119.9
C5—Ir1—C9	86.15 (11)	C209—C210—C211	120.4 (3)
C8—Ir1—C9	36.88 (11)	C209—C210—H210	119.8
C4—Ir1—C1	85.76 (11)	C211—C210—H210	119.8
C5—Ir1—C1	93.77 (11)	C210—C211—C212	120.4 (3)
C8—Ir1—C1	168.27 (11)	C210—C211—H211	119.8
C9—Ir1—C1	152.78 (11)	C212—C211—H211	119.8
C4—Ir1—P4	123.12 (9)	C211—C212—C207	119.2 (3)
C5—Ir1—P4	160.77 (9)	C211—C212—H212	120.4
C8—Ir1—P4	103.01 (8)	C207—C212—H212	120.4
C9—Ir1—P4	84.52 (8)	C306—C301—C302	119.9 (3)
C1—Ir1—P4	86.97 (7)	C306—C301—P3	122.0 (2)
C4—Ir1—P1	137.87 (9)	C302—C301—P3	117.9 (2)
C5—Ir1—P1	101.15 (9)	C303—C302—C301	119.9 (4)
C8—Ir1—P1	84.27 (8)	C303—C302—H302	120.1
C9—Ir1—P1	118.50 (8)	C301—C302—H302	120.1
C1—Ir1—P1	88.27 (7)	C304—C303—C302	120.0 (4)
P4—Ir1—P1	98.08 (2)	C304—C303—H303	120.0
C101—P1—C2	104.16 (13)	C302—C303—H303	120.0
C101—P1—C107	98.08 (13)	C305—C304—C303	120.4 (4)
C2—P1—C107	99.81 (13)	C305—C304—H304	119.8
C101—P1—Ir1	121.11 (9)	C303—C304—H304	119.8
C2—P1—Ir1	104.80 (9)	C304—C305—C306	120.6 (4)
C107—P1—Ir1	125.41 (9)	C304—C305—H305	119.7
C2—P2—C201	107.29 (13)	C306—C305—H305	119.7
C2—P2—C207	110.72 (13)	C301—C306—C305	119.2 (3)
C201—P2—C207	102.84 (13)	C301—C306—H306	120.4
C2—P2—C1	106.75 (12)	C305—C306—H306	120.4
C201—P2—C1	119.19 (13)	C308—C307—C312	119.7 (3)
C207—P2—C1	109.95 (13)	C308—C307—P3	123.6 (2)
C3—P3—C307	109.67 (13)	C312—C307—P3	116.1 (2)
C3—P3—C301	108.36 (14)	C307—C308—C309	118.5 (3)
C307—P3—C301	101.47 (13)	C307—C308—H308	120.7
C3—P3—C1	104.15 (13)	C309—C308—H308	120.7
C307—P3—C1	113.63 (13)	C310—C309—C308	121.3 (3)
C301—P3—C1	119.41 (13)	C310—C309—H309	119.4
C401—P4—C3	102.37 (13)	C308—C309—H309	119.4
C401—P4—C407	98.90 (13)	C309—C310—C311	120.2 (3)
C3—P4—C407	100.46 (13)	C309—C310—H310	119.9
C401—P4—Ir1	122.24 (10)	C311—C310—H310	119.9
C3—P4—Ir1	107.96 (9)	C310—C311—C312	119.6 (4)
C407—P4—Ir1	121.49 (10)	C310—C311—H311	120.2
P3—C1—P2	114.86 (14)	C312—C311—H311	120.2
P3—C1—Ir1	109.99 (13)	C311—C312—C307	120.7 (3)
P2—C1—Ir1	111.40 (13)	C311—C312—H312	119.7
P3—C1—H1	106.6 (15)	C307—C312—H312	119.7
P2—C1—H1	102.8 (14)	C402—C401—C406	118.6 (3)
Ir1—C1—H1	110.9 (14)	C402—C401—P4	118.2 (2)
P2—C2—P1	107.11 (13)	C406—C401—P4	123.0 (2)
P2—C2—H2A	110.3	C403—C402—C401	120.4 (3)
P1—C2—H2A	110.3	C403—C402—H402	119.8
P2—C2—H2B	110.3	C401—C402—H402	119.8
P1—C2—H2B	110.3	C404—C403—C402	120.4 (3)
H2A—C2—H2B	108.5	C404—C403—H403	119.8
P3—C3—P4	105.75 (14)	C402—C403—H403	119.8
P3—C3—H3A	110.6	C405—C404—C403	119.8 (3)
P4—C3—H3A	110.6	C405—C404—H404	120.1
P3—C3—H3B	110.6	C403—C404—H404	120.1
P4—C3—H3B	110.6	C404—C405—C406	120.3 (3)
H3A—C3—H3B	108.7	C404—C405—H405	119.8
C5—C4—C11	121.6 (3)	C406—C405—H405	119.8
C5—C4—Ir1	71.10 (16)	C405—C406—C401	120.4 (3)
C11—C4—Ir1	116.4 (2)	C405—C406—H406	119.8
C5—C4—H4	116.5 (18)	C401—C406—H406	119.8
C11—C4—H4	112.7 (17)	C408—C407—C412	118.4 (3)
Ir1—C4—H4	112.0 (17)	C408—C407—P4	121.2 (2)
C4—C5—C6	121.6 (3)	C412—C407—P4	120.2 (2)
C4—C5—Ir1	70.87 (17)	C409—C408—C407	120.6 (3)
C6—C5—Ir1	115.47 (19)	C409—C408—H408	119.7
C4—C5—H5	113.5 (19)	C407—C408—H408	119.7
C6—C5—H5	116.7 (19)	C410—C409—C408	120.2 (3)
Ir1—C5—H5	109.7 (18)	C410—C409—H409	119.9
C5—C6—C7	112.7 (2)	C408—C409—H409	119.9
C5—C6—H6A	109.1	C409—C410—C411	120.2 (3)
C7—C6—H6A	109.1	C409—C410—H410	119.9
C5—C6—H6B	109.1	C411—C410—H410	119.9
C7—C6—H6B	109.1	C410—C411—C412	119.9 (3)
H6A—C6—H6B	107.8	C410—C411—H411	120.1
C8—C7—C6	112.2 (2)	C412—C411—H411	120.1
C8—C7—H7A	109.2	C411—C412—C407	120.7 (3)
C6—C7—H7A	109.2	C411—C412—H412	119.7
C8—C7—H7B	109.2	C407—C412—H412	119.7
C6—C7—H7B	109.2	C14—O8—C16	116.9 (3)
H7A—C7—H7B	107.9	O3—S1—O2	119.2 (8)
C9—C8—C7	123.2 (3)	O3—S1—O1	111.2 (6)
C9—C8—Ir1	72.21 (16)	O2—S1—O1	114.1 (8)
C7—C8—Ir1	112.51 (19)	O3—S1—C12	102.1 (6)
C9—C8—H8	113.8 (17)	O2—S1—C12	104.7 (8)
C7—C8—H8	117.3 (18)	O1—S1—C12	103.0 (4)
Ir1—C8—H8	107.5 (17)	F2—C12—F1	111.5 (7)
C8—C9—C10	122.9 (3)	F2—C12—F3	110.8 (10)
C8—C9—Ir1	70.91 (16)	F1—C12—F3	101.6 (7)
C10—C9—Ir1	115.7 (2)	F2—C12—S1	110.1 (6)
C8—C9—H9	117.0 (17)	F1—C12—S1	115.8 (4)
C10—C9—H9	112.8 (17)	F3—C12—S1	106.6 (7)
Ir1—C9—H9	109.7 (17)	O6—S2—O5	115.7 (3)
C9—C10—C11	113.1 (2)	O6—S2—O4	116.9 (3)
C9—C10—H10A	109.0	O5—S2—O4	115.62 (18)
C11—C10—H10A	109.0	O6—S2—C13	104.7 (4)
C9—C10—H10B	109.0	O5—S2—C13	99.4 (2)
C11—C10—H10B	109.0	O4—S2—C13	100.7 (3)
H10A—C10—H10B	107.8	F6—C13—F5	106.3 (9)
C4—C11—C10	112.1 (2)	F6—C13—F4	107.3 (9)
C4—C11—H11A	109.2	F5—C13—F4	106.2 (9)
C10—C11—H11A	109.2	F6—C13—S2	109.9 (6)
C4—C11—H11B	109.2	F5—C13—S2	112.8 (7)
C10—C11—H11B	109.2	F4—C13—S2	113.9 (7)
H11A—C11—H11B	107.9	O2A—S1A—O3A	122 (2)
O7—C14—O8	122.0 (4)	O2A—S1A—O1A	112 (3)
O7—C14—C15	124.8 (4)	O3A—S1A—O1A	114 (2)
O8—C14—C15	113.2 (3)	O6A—S2A—C13A	98.6 (12)
C14—C15—H15A	109.5	F4A—C13A—F5A	115 (2)
C14—C15—H15B	109.5	F4A—C13A—F6A	103.4 (19)
H15A—C15—H15B	109.5	F5A—C13A—F6A	103.0 (19)
C14—C15—H15C	109.5	F4A—C13A—S2A	109.7 (15)
H15A—C15—H15C	109.5	F5A—C13A—S2A	115.7 (17)
H15B—C15—H15C	109.5	F6A—C13A—S2A	108.7 (13)
C17—C16—O8	109.1 (5)	Cl1A—C18—Cl2B	107.9 (14)
C17—C16—H16A	109.9	Cl1A—C18—Cl2	87.6 (11)
O8—C16—H16A	109.9	Cl2B—C18—Cl2	25.8 (14)
C17—C16—H16B	109.9	Cl1A—C18—Cl1	23.8 (9)
O8—C16—H16B	109.9	Cl2B—C18—Cl1	131.0 (12)
H16A—C16—H16B	108.3	Cl2—C18—Cl1	111.4 (8)
C16—C17—H17A	109.5	Cl1A—C18—C18A	63.8 (14)
C16—C17—H17B	109.5	Cl2B—C18—C18A	47.8 (18)
H17A—C17—H17B	109.5	Cl2—C18—C18A	24.0 (14)
C16—C17—H17C	109.5	Cl1—C18—C18A	87.5 (12)
H17A—C17—H17C	109.5	Cl1A—C18—Cl2A	90.0 (9)
H17B—C17—H17C	109.5	Cl2B—C18—Cl2A	21.8 (12)
C106—C101—C102	119.2 (3)	Cl2—C18—Cl2A	29.1 (5)
C106—C101—P1	123.6 (2)	Cl1—C18—Cl2A	111.4 (6)
C102—C101—P1	117.1 (2)	C18A—C18—Cl2A	40.3 (16)
C103—C102—C101	120.3 (3)	Cl1A—Cl1—C18	32.9 (12)
C103—C102—H102	119.9	Cl2B—Cl2—C18A	141 (6)
C101—C102—H102	119.9	Cl2B—Cl2—Cl2A	55 (3)
C104—C103—C102	119.8 (3)	C18A—Cl2—Cl2A	100 (4)
C104—C103—H103	120.1	Cl2B—Cl2—C18	61 (3)
C102—C103—H103	120.1	C18A—Cl2—C18	94 (4)
C105—C104—C103	120.2 (3)	Cl2A—Cl2—C18	92.1 (12)
C105—C104—H104	119.9	Cl2B—Cl2—Cl1A	91 (3)
C103—C104—H104	119.9	C18A—Cl2—Cl1A	59 (3)
C104—C105—C106	120.8 (3)	Cl2A—Cl2—Cl1A	94.3 (15)
C104—C105—H105	119.6	C18—Cl2—Cl1A	36.0 (5)
C106—C105—H105	119.6	Cl2—Cl2B—Cl2A	76 (3)
C105—C106—C101	119.7 (3)	Cl2—Cl2B—C18A	20 (3)
C105—C106—H106	120.2	Cl2A—Cl2B—C18A	67 (3)
C101—C106—H106	120.2	Cl2—Cl2B—C18	93 (3)
C108—C107—C112	118.1 (3)	Cl2A—Cl2B—C18	116 (3)
C108—C107—P1	123.7 (2)	C18A—Cl2B—C18	82 (2)
C112—C107—P1	118.1 (2)	Cl2—Cl2B—Cl1A	69 (2)
C109—C108—C107	121.0 (3)	Cl2A—Cl2B—Cl1A	89 (2)
C109—C108—H108	119.5	C18A—Cl2B—Cl1A	53.7 (19)
C107—C108—H108	119.5	C18—Cl2B—Cl1A	31.5 (7)
C110—C109—C108	120.3 (3)	Cl2—C18A—Cl2A	45 (3)
C110—C109—H109	119.9	Cl2—C18A—Cl2B	19 (3)
C108—C109—H109	119.9	Cl2A—C18A—Cl2B	33.9 (19)
C109—C110—C111	119.4 (3)	Cl2—C18A—Cl1A	99 (3)
C109—C110—H110	120.3	Cl2A—C18A—Cl1A	96 (2)
C111—C110—H110	120.3	Cl2B—C18A—Cl1A	85 (2)
C112—C111—C110	120.6 (3)	Cl2—C18A—C18	62 (3)
C112—C111—H111	119.7	Cl2A—C18A—C18	72.2 (16)
C110—C111—H111	119.7	Cl2B—C18A—C18	50.0 (16)
C111—C112—C107	120.6 (3)	Cl1A—C18A—C18	37.6 (9)
C111—C112—H112	119.7	Cl1—Cl1A—C18	123.3 (19)
C107—C112—H112	119.7	Cl1—Cl1A—C18A	157 (2)
C206—C201—C202	119.8 (3)	C18—Cl1A—C18A	78.6 (13)
C206—C201—P2	121.4 (2)	Cl1—Cl1A—Cl2	177 (2)
C202—C201—P2	118.7 (2)	C18—Cl1A—Cl2	56.4 (9)
C203—C202—C201	119.7 (3)	C18A—Cl1A—Cl2	22.4 (10)
C203—C202—H202	120.2	Cl1—Cl1A—Cl2B	161 (2)
C201—C202—H202	120.2	C18—Cl1A—Cl2B	40.6 (11)
C202—C203—C204	119.7 (3)	C18A—Cl1A—Cl2B	41.2 (15)
C202—C203—H203	120.1	Cl2—Cl1A—Cl2B	19.9 (11)
C204—C203—H203	120.1	Cl1—Cl1A—Cl2A	158 (2)
C205—C204—C203	121.0 (3)	C18—Cl1A—Cl2A	58.8 (8)
C205—C204—H204	119.5	C18A—Cl1A—Cl2A	35.3 (16)
C203—C204—H204	119.5	Cl2—Cl1A—Cl2A	24.5 (4)
C204—C205—C206	120.1 (3)	Cl2B—Cl1A—Cl2A	20.6 (9)
C204—C205—H205	120.0	Cl2B—Cl2A—Cl2	48 (3)
C206—C205—H205	120.0	Cl2B—Cl2A—C18A	79 (4)
C205—C206—C201	119.8 (3)	Cl2—Cl2A—C18A	35.0 (18)
C205—C206—H206	120.1	Cl2B—Cl2A—C18	43 (3)
C201—C206—H206	120.1	Cl2—Cl2A—C18	58.8 (10)
C208—C207—C212	119.5 (3)	C18A—Cl2A—C18	67.5 (18)
C208—C207—P2	122.7 (2)	Cl2B—Cl2A—Cl1A	70 (3)
C212—C207—P2	117.1 (2)	Cl2—Cl2A—Cl1A	61.2 (15)
C209—C208—C207	120.2 (3)	C18A—Cl2A—Cl1A	49.1 (18)
C209—C208—H208	119.9	C18—Cl2A—Cl1A	31.1 (5)

### (Cycloocta-1,5-diene)(1,1,3,3,5,5,7,7-octaphenyl-1,7-diphospha-3,5-diphosphoniaheptan-4-yl)iridium(I) bis(trifluoromethanesulfonate)–ethyl acetate–dichloromethane (1/1/1) (1b) Hydrogen-bond geometry (Å, º)

**Table:**

*D*—H···*A*	*D*—H	H···*A*	*D*···*A*	*D*—H···*A*
C2—H2*B*···O4	0.98	2.36	3.245 (4)	151
C2—H2*A*···O5^i^	0.98	2.38	3.307 (4)	158
C3—H3*B*···O5^i^	0.98	2.38	3.343 (4)	169
C206—H206···O5^i^	0.94	2.52	3.204 (4)	130
C3—H3*A*···O7	0.98	2.23	3.185 (5)	165
C1—H1···O3	0.94 (2)	2.55 (2)	3.419 (11)	155 (2)
C208—H208···O3	0.94	2.47	3.231 (16)	139
C308—H308···O3	0.94	2.57	3.301 (12)	135

Symmetry code: (i) −*x*+1, −*y*+1, −*z*+1.

### Dichloridohydrido(1,1,3,3,5,5,7,7-octaphenyl-1,5λ5,7-triphospha-3-phosphoniahept-4-en-4-yl)iridium(III) acetone monosolvate (2) Crystal data

**Table:**

[Ir(C_51_H_44_P_4_)ClH]Cl·C_3_H_6_O	*F*(000) = 2216
*M**_r_* = 1102.93	*D*_x_ = 1.536 Mg m^−^^3^
Monoclinic, *P*2_1_/*n*	Mo *K*α radiation, λ = 0.71073 Å
*a* = 18.7964 (4) Å	Cell parameters from 78297 reflections
*b* = 13.7444 (2) Å	θ = 1.0–25.3°
*c* = 18.8487 (4) Å	µ = 3.08 mm^−^^1^
β = 101.586 (2)°	*T* = 233 K
*V* = 4770.25 (16) Å^3^	Prism, orange
*Z* = 4	0.15 × 0.12 × 0.02 mm

### Dichloridohydrido(1,1,3,3,5,5,7,7-octaphenyl-1,5λ5,7-triphospha-3-phosphoniahept-4-en-4-yl)iridium(III) acetone monosolvate (2) Data collection

**Table:**

Nonius KappaCCD diffractometer	6069 reflections with *I* > 2σ(*I*)
Radiation source: fine-focus sealed tube	*R*_int_ = 0.083
Graphite monochromator	θ_max_ = 25.0°, θ_min_ = 1.7°
phi– and ω scans	*h* = −22→20
27235 measured reflections	*k* = −16→16
8407 independent reflections	*l* = −22→22

### Dichloridohydrido(1,1,3,3,5,5,7,7-octaphenyl-1,5λ5,7-triphospha-3-phosphoniahept-4-en-4-yl)iridium(III) acetone monosolvate (2) Refinement

**Table:**

Refinement on *F*^2^	Primary atom site location: structure-invariant direct methods
Least-squares matrix: full	Secondary atom site location: difference Fourier map
*R*[*F*^2^ > 2σ(*F*^2^)] = 0.048	Hydrogen site location: inferred from neighbouring sites
*wR*(*F*^2^) = 0.091	H-atom parameters constrained
*S* = 1.04	*w* = 1/[σ^2^(*F*_o_^2^) + (0.0337*P*)^2^ + 5.179*P*] where *P* = (*F*_o_^2^ + 2*F*_c_^2^)/3
8407 reflections	(Δ/σ)_max_ = 0.003
572 parameters	Δρ_max_ = 0.92 e Å^−^^3^
1 restraint	Δρ_min_ = −0.80 e Å^−^^3^

### Dichloridohydrido(1,1,3,3,5,5,7,7-octaphenyl-1,5λ5,7-triphospha-3-phosphoniahept-4-en-4-yl)iridium(III) acetone monosolvate (2) Special details

**Table:**

Geometry. All esds (except the esd in the dihedral angle between two l.s. planes) are estimated using the full covariance matrix. The cell esds are taken into account individually in the estimation of esds in distances, angles and torsion angles; correlations between esds in cell parameters are only used when they are defined by crystal symmetry. An approximate (isotropic) treatment of cell esds is used for estimating esds involving l.s. planes.
Refinement. Small crystal with low diffraction at higher 2 theta angles. Hydrogen atom at Ir was localized and refined isotropically with bond restraint (*d* = 1.6 Å), because of a too long bond distance of 1.88 Å by free refinement. The solvent molecule aceton is slightly disordered with one solved positional disorder for a methyl group C6:C6A at ratio 1:1. Hydrogen atoms at solvent could not be localized and were omitted.

### Dichloridohydrido(1,1,3,3,5,5,7,7-octaphenyl-1,5λ5,7-triphospha-3-phosphoniahept-4-en-4-yl)iridium(III) acetone monosolvate (2) Fractional atomic coordinates and isotropic or equivalent isotropic displacement parameters (Å^2^)

**Table:**

	*x*	*y*	*z*	*U*_iso_*/*U*_eq_	Occ. (<1)
Ir1	0.091292 (12)	1.211417 (16)	0.715827 (11)	0.03564 (9)	
H1	0.104 (3)	1.166 (4)	0.7978 (15)	0.060 (17)*	
P1	0.20108 (8)	1.29145 (11)	0.73550 (7)	0.0364 (3)	
P2	0.10554 (9)	1.40730 (11)	0.80556 (8)	0.0414 (4)	
P3	−0.04557 (9)	1.35866 (11)	0.72257 (8)	0.0411 (4)	
P4	−0.02175 (9)	1.14531 (11)	0.70562 (8)	0.0416 (4)	
Cl1	0.14205 (9)	1.05981 (10)	0.68023 (8)	0.0483 (4)	
Cl2	0.05445 (9)	1.27503 (11)	0.58858 (8)	0.0498 (4)	
C1	0.0437 (3)	1.3354 (4)	0.7520 (3)	0.0399 (14)	
C2	0.1830 (3)	1.4166 (4)	0.7621 (3)	0.0422 (15)	
H2A	0.2252	1.4430	0.7958	0.051*	
H2B	0.1719	1.4590	0.7195	0.051*	
C3	−0.0859 (3)	1.2459 (4)	0.6821 (3)	0.0453 (15)	
H3A	−0.0986	1.2530	0.6293	0.054*	
H3B	−0.1305	1.2319	0.6997	0.054*	
C101	0.2483 (3)	1.3090 (4)	0.6612 (3)	0.0386 (14)	
C102	0.2438 (4)	1.2373 (4)	0.6093 (3)	0.0508 (17)	
H102	0.2165	1.1808	0.6127	0.061*	
C103	0.2795 (4)	1.2484 (6)	0.5519 (4)	0.0620 (19)	
H103	0.2777	1.1981	0.5179	0.074*	
C104	0.3167 (4)	1.3310 (6)	0.5446 (3)	0.0595 (19)	
H104	0.3389	1.3386	0.5044	0.071*	
C105	0.3223 (4)	1.4029 (5)	0.5949 (4)	0.0576 (18)	
H105	0.3489	1.4596	0.5901	0.069*	
C106	0.2884 (4)	1.3919 (4)	0.6535 (3)	0.0502 (17)	
H106	0.2927	1.4414	0.6885	0.060*	
C107	0.2719 (3)	1.2471 (4)	0.8099 (3)	0.0390 (14)	
C108	0.2642 (4)	1.1569 (5)	0.8398 (3)	0.0560 (18)	
H108	0.2241	1.1177	0.8202	0.067*	
C109	0.3155 (4)	1.1235 (5)	0.8989 (4)	0.068 (2)	
H109	0.3097	1.0622	0.9192	0.082*	
C110	0.3746 (4)	1.1806 (6)	0.9274 (4)	0.064 (2)	
H110	0.4084	1.1593	0.9681	0.076*	
C111	0.3840 (4)	1.2682 (5)	0.8964 (4)	0.0597 (19)	
H111	0.4253	1.3059	0.9149	0.072*	
C112	0.3331 (3)	1.3019 (4)	0.8379 (3)	0.0494 (16)	
H112	0.3401	1.3624	0.8170	0.059*	
C201	0.0773 (3)	1.5290 (4)	0.8238 (4)	0.0484 (16)	
C202	0.0844 (4)	1.6065 (5)	0.7788 (4)	0.066 (2)	
H202	0.1059	1.5968	0.7384	0.080*	
C203	0.0603 (5)	1.6974 (5)	0.7929 (5)	0.088 (3)	
H203	0.0655	1.7498	0.7624	0.105*	
C204	0.0284 (5)	1.7116 (6)	0.8517 (6)	0.097 (3)	
H204	0.0124	1.7742	0.8612	0.117*	
C205	0.0194 (5)	1.6355 (7)	0.8970 (5)	0.088 (3)	
H205	−0.0038	1.6451	0.9362	0.105*	
C206	0.0455 (4)	1.5443 (5)	0.8831 (4)	0.071 (2)	
H206	0.0415	1.4922	0.9144	0.085*	
C207	0.1427 (3)	1.3566 (5)	0.8949 (3)	0.0465 (16)	
C208	0.1057 (4)	1.2834 (6)	0.9204 (4)	0.069 (2)	
H208	0.0621	1.2606	0.8918	0.083*	
C209	0.1316 (7)	1.2419 (6)	0.9881 (5)	0.100 (3)	
H209	0.1063	1.1910	1.0055	0.121*	
C210	0.1956 (7)	1.2778 (9)	1.0290 (4)	0.103 (3)	
H210	0.2131	1.2521	1.0754	0.123*	
C211	0.2336 (5)	1.3493 (8)	1.0037 (4)	0.090 (3)	
H211	0.2782	1.3703	1.0315	0.108*	
C212	0.2068 (4)	1.3912 (6)	0.9369 (3)	0.068 (2)	
H212	0.2319	1.4427	0.9202	0.082*	
C301	−0.0779 (3)	1.4458 (4)	0.6504 (3)	0.0442 (15)	
C302	−0.0310 (4)	1.5011 (5)	0.6200 (4)	0.0568 (18)	
H302	0.0193	1.4960	0.6376	0.068*	
C303	−0.0574 (4)	1.5642 (5)	0.5636 (4)	0.0626 (19)	
H303	−0.0250	1.6025	0.5434	0.075*	
C304	−0.1301 (4)	1.5710 (5)	0.5374 (4)	0.0627 (19)	
H304	−0.1481	1.6146	0.4996	0.075*	
C305	−0.1768 (4)	1.5148 (6)	0.5658 (4)	0.068 (2)	
H305	−0.2270	1.5187	0.5469	0.081*	
C306	−0.1513 (4)	1.4528 (5)	0.6217 (4)	0.0568 (18)	
H306	−0.1842	1.4143	0.6409	0.068*	
C307	−0.0915 (3)	1.3948 (5)	0.7946 (3)	0.0517 (17)	
C308	−0.0883 (4)	1.3334 (5)	0.8531 (4)	0.071 (2)	
H308	−0.0660	1.2722	0.8530	0.086*	
C309	−0.1174 (6)	1.3603 (7)	0.9116 (5)	0.096 (3)	
H309	−0.1154	1.3174	0.9508	0.116*	
C310	−0.1487 (6)	1.4480 (9)	0.9128 (5)	0.104 (3)	
H310	−0.1679	1.4657	0.9533	0.125*	
C311	−0.1533 (6)	1.5123 (7)	0.8568 (5)	0.102 (3)	
H311	−0.1755	1.5734	0.8579	0.123*	
C312	−0.1237 (5)	1.4838 (6)	0.7978 (4)	0.079 (2)	
H312	−0.1260	1.5271	0.7588	0.095*	
C401	−0.0525 (4)	1.0532 (5)	0.6373 (4)	0.0513 (17)	
C402	−0.0726 (5)	1.0752 (6)	0.5658 (4)	0.090 (3)	
H402	−0.0738	1.1407	0.5512	0.107*	
C403	−0.0912 (6)	1.0041 (8)	0.5143 (5)	0.104 (3)	
H403	−0.1039	1.0221	0.4653	0.124*	
C404	−0.0919 (5)	0.9113 (8)	0.5315 (6)	0.097 (3)	
H404	−0.1101	0.8642	0.4964	0.116*	
C405	−0.0649 (5)	0.8847 (6)	0.6030 (6)	0.094 (3)	
H405	−0.0603	0.8186	0.6160	0.112*	
C406	−0.0447 (5)	0.9565 (6)	0.6550 (4)	0.087 (3)	
H406	−0.0255	0.9385	0.7031	0.105*	
C407	−0.0473 (4)	1.0910 (4)	0.7860 (3)	0.0489 (16)	
C408	0.0043 (4)	1.0520 (6)	0.8402 (4)	0.078 (2)	
H408	0.0536	1.0555	0.8374	0.093*	
C409	−0.0153 (6)	1.0078 (8)	0.8987 (5)	0.106 (3)	
H409	0.0207	0.9795	0.9346	0.127*	
C410	−0.0847 (7)	1.0042 (7)	0.9054 (5)	0.105 (3)	
H410	−0.0971	0.9751	0.9464	0.126*	
C411	−0.1366 (6)	1.0424 (8)	0.8535 (7)	0.118 (4)	
H411	−0.1853	1.0418	0.8588	0.142*	
C412	−0.1187 (5)	1.0824 (7)	0.7922 (5)	0.087 (3)	
H412	−0.1557	1.1040	0.7543	0.105*	
O1	−0.2574 (4)	1.2344 (8)	0.7189 (4)	0.159 (4)	
C4	−0.2949 (6)	1.2620 (9)	0.7592 (6)	0.104 (3)	
C5	−0.2930 (10)	1.2258 (12)	0.8318 (8)	0.199 (7)	
C6	−0.3726 (15)	1.290 (3)	0.7273 (13)	0.158 (12)	0.50
C6A	−0.3108 (18)	1.366 (2)	0.7492 (19)	0.176 (13)	0.50

### Dichloridohydrido(1,1,3,3,5,5,7,7-octaphenyl-1,5λ5,7-triphospha-3-phosphoniahept-4-en-4-yl)iridium(III) acetone monosolvate (2) Atomic displacement parameters (Å^2^)

**Table:**

	*U*^11^	*U*^22^	*U*^33^	*U*^12^	*U*^13^	*U*^23^
Ir1	0.04171 (15)	0.03396 (13)	0.02948 (13)	0.00138 (12)	0.00290 (9)	−0.00100 (11)
P1	0.0445 (9)	0.0337 (8)	0.0292 (7)	0.0008 (8)	0.0032 (7)	−0.0009 (7)
P2	0.0481 (10)	0.0396 (9)	0.0345 (9)	0.0033 (8)	0.0038 (7)	−0.0046 (7)
P3	0.0464 (9)	0.0440 (9)	0.0325 (8)	0.0046 (8)	0.0074 (7)	0.0003 (7)
P4	0.0473 (10)	0.0427 (9)	0.0334 (8)	−0.0019 (8)	0.0048 (7)	−0.0021 (7)
Cl1	0.0570 (10)	0.0393 (8)	0.0454 (9)	0.0049 (7)	0.0027 (7)	−0.0038 (7)
Cl2	0.0592 (10)	0.0549 (10)	0.0340 (8)	0.0071 (8)	0.0061 (7)	0.0051 (7)
C1	0.046 (4)	0.039 (3)	0.034 (3)	0.001 (3)	0.008 (3)	−0.006 (3)
C2	0.050 (4)	0.035 (3)	0.041 (3)	−0.008 (3)	0.006 (3)	0.001 (3)
C3	0.046 (4)	0.049 (3)	0.041 (4)	−0.006 (3)	0.009 (3)	0.000 (3)
C101	0.042 (4)	0.035 (3)	0.037 (3)	0.001 (3)	0.002 (3)	−0.001 (3)
C102	0.054 (4)	0.049 (4)	0.050 (4)	−0.005 (3)	0.013 (3)	−0.009 (3)
C103	0.065 (5)	0.077 (5)	0.049 (4)	−0.006 (4)	0.021 (4)	−0.016 (4)
C104	0.056 (4)	0.084 (5)	0.042 (4)	−0.001 (4)	0.017 (3)	0.007 (4)
C105	0.054 (4)	0.058 (4)	0.065 (5)	−0.011 (4)	0.021 (4)	0.004 (4)
C106	0.058 (4)	0.048 (4)	0.045 (4)	0.000 (3)	0.008 (3)	−0.004 (3)
C107	0.041 (4)	0.038 (3)	0.035 (3)	0.005 (3)	0.001 (3)	0.000 (3)
C108	0.065 (5)	0.052 (4)	0.047 (4)	0.000 (4)	0.003 (3)	−0.001 (3)
C109	0.072 (5)	0.063 (4)	0.060 (5)	0.004 (4)	−0.010 (4)	0.018 (4)
C110	0.060 (5)	0.075 (5)	0.049 (4)	0.014 (4)	−0.007 (4)	0.000 (4)
C111	0.045 (4)	0.070 (5)	0.058 (4)	0.002 (4)	−0.005 (3)	−0.007 (4)
C112	0.047 (4)	0.051 (4)	0.047 (4)	−0.001 (3)	0.002 (3)	0.006 (3)
C201	0.043 (4)	0.039 (4)	0.060 (4)	0.002 (3)	0.005 (3)	−0.012 (3)
C202	0.077 (5)	0.044 (4)	0.080 (5)	0.009 (4)	0.020 (4)	−0.009 (4)
C203	0.105 (7)	0.054 (5)	0.110 (7)	0.006 (5)	0.035 (6)	−0.011 (5)
C204	0.095 (7)	0.055 (5)	0.149 (9)	0.003 (5)	0.043 (7)	−0.036 (6)
C205	0.096 (7)	0.079 (6)	0.093 (6)	0.016 (5)	0.029 (5)	−0.028 (5)
C206	0.086 (6)	0.058 (5)	0.069 (5)	0.008 (4)	0.019 (4)	−0.019 (4)
C207	0.057 (4)	0.055 (4)	0.027 (3)	0.008 (4)	0.009 (3)	−0.007 (3)
C208	0.094 (6)	0.074 (5)	0.037 (4)	0.007 (5)	0.006 (4)	0.003 (4)
C209	0.162 (10)	0.084 (6)	0.055 (5)	0.014 (6)	0.020 (6)	0.020 (5)
C210	0.124 (9)	0.140 (9)	0.039 (5)	0.050 (8)	0.007 (5)	0.001 (6)
C211	0.082 (6)	0.144 (9)	0.036 (5)	0.035 (6)	−0.007 (4)	−0.008 (5)
C212	0.069 (5)	0.102 (6)	0.031 (4)	0.011 (4)	0.002 (4)	−0.018 (4)
C301	0.046 (4)	0.050 (4)	0.035 (3)	0.008 (3)	0.005 (3)	0.005 (3)
C302	0.054 (4)	0.056 (4)	0.058 (4)	0.000 (4)	0.005 (4)	0.010 (4)
C303	0.063 (5)	0.066 (5)	0.057 (4)	−0.009 (4)	0.008 (4)	0.026 (4)
C304	0.067 (5)	0.073 (5)	0.045 (4)	0.014 (4)	0.004 (4)	0.016 (4)
C305	0.054 (5)	0.098 (6)	0.049 (4)	0.014 (4)	0.004 (4)	0.016 (4)
C306	0.045 (4)	0.074 (5)	0.052 (4)	0.007 (4)	0.012 (3)	0.015 (4)
C307	0.051 (4)	0.061 (4)	0.044 (4)	0.011 (4)	0.010 (3)	−0.002 (3)
C308	0.096 (6)	0.065 (5)	0.064 (5)	0.014 (4)	0.042 (5)	0.005 (4)
C309	0.146 (9)	0.088 (6)	0.075 (6)	0.019 (6)	0.069 (6)	0.015 (5)
C310	0.123 (8)	0.130 (9)	0.074 (6)	0.023 (7)	0.052 (6)	−0.008 (6)
C311	0.136 (9)	0.107 (7)	0.076 (6)	0.056 (6)	0.051 (6)	−0.012 (6)
C312	0.101 (6)	0.081 (6)	0.061 (5)	0.030 (5)	0.030 (5)	0.009 (4)
C401	0.059 (4)	0.048 (4)	0.050 (4)	−0.008 (3)	0.018 (3)	−0.006 (3)
C402	0.135 (8)	0.076 (5)	0.053 (5)	−0.021 (5)	0.008 (5)	−0.020 (4)
C403	0.147 (10)	0.100 (7)	0.061 (6)	−0.012 (7)	0.014 (6)	−0.040 (5)
C404	0.088 (7)	0.112 (8)	0.088 (7)	−0.027 (6)	0.011 (5)	−0.061 (6)
C405	0.118 (8)	0.058 (5)	0.111 (8)	−0.021 (5)	0.039 (6)	−0.026 (5)
C406	0.125 (8)	0.066 (5)	0.069 (5)	−0.010 (5)	0.013 (5)	−0.018 (4)
C407	0.053 (4)	0.044 (4)	0.051 (4)	−0.012 (3)	0.013 (3)	−0.001 (3)
C408	0.074 (5)	0.097 (6)	0.064 (5)	−0.005 (5)	0.017 (4)	0.029 (5)
C409	0.098 (7)	0.149 (9)	0.068 (6)	−0.028 (7)	0.010 (5)	0.047 (6)
C410	0.154 (11)	0.100 (7)	0.072 (6)	−0.015 (7)	0.045 (7)	0.034 (5)
C411	0.118 (9)	0.123 (8)	0.137 (9)	0.020 (7)	0.081 (8)	0.055 (8)
C412	0.081 (6)	0.102 (7)	0.084 (6)	−0.001 (5)	0.028 (5)	0.035 (5)
O1	0.099 (6)	0.283 (11)	0.094 (5)	0.037 (6)	0.016 (4)	−0.028 (6)
C4	0.089 (8)	0.143 (10)	0.082 (7)	0.004 (7)	0.021 (6)	−0.016 (7)
C5	0.28 (2)	0.230 (16)	0.111 (11)	0.078 (14)	0.086 (12)	0.035 (10)
C6	0.109 (18)	0.25 (4)	0.113 (18)	0.09 (2)	0.018 (15)	0.00 (2)
C6A	0.15 (3)	0.14 (2)	0.26 (4)	0.04 (2)	0.09 (3)	0.08 (2)

### Dichloridohydrido(1,1,3,3,5,5,7,7-octaphenyl-1,5λ5,7-triphospha-3-phosphoniahept-4-en-4-yl)iridium(III) acetone monosolvate (2) Geometric parameters (Å, º)

**Table:**

Ir1—C1	2.101 (5)	C208—C209	1.394 (11)
Ir1—P4	2.2831 (16)	C208—H208	0.9400
Ir1—P1	2.3019 (15)	C209—C210	1.382 (15)
Ir1—Cl1	2.4412 (15)	C209—H209	0.9400
Ir1—Cl2	2.5157 (14)	C210—C211	1.358 (13)
Ir1—H1	1.638 (19)	C210—H210	0.9400
P1—C101	1.819 (6)	C211—C212	1.383 (10)
P1—C107	1.834 (6)	C211—H211	0.9400
P1—C2	1.842 (6)	C212—H212	0.9400
P2—C1	1.695 (6)	C301—C302	1.372 (9)
P2—C201	1.809 (6)	C301—C306	1.381 (8)
P2—C2	1.813 (6)	C302—C303	1.385 (9)
P2—C207	1.827 (6)	C302—H302	0.9400
P3—C1	1.688 (6)	C303—C304	1.361 (9)
P3—C307	1.817 (6)	C303—H303	0.9400
P3—C301	1.823 (6)	C304—C305	1.358 (10)
P3—C3	1.824 (6)	C304—H304	0.9400
P4—C401	1.816 (6)	C305—C306	1.365 (9)
P4—C3	1.829 (6)	C305—H305	0.9400
P4—C407	1.837 (6)	C306—H306	0.9400
C2—H2A	0.9800	C307—C312	1.372 (9)
C2—H2B	0.9800	C307—C308	1.382 (9)
C3—H3A	0.9800	C308—C309	1.377 (10)
C3—H3B	0.9800	C308—H308	0.9400
C101—C102	1.378 (8)	C309—C310	1.343 (12)
C101—C106	1.390 (8)	C309—H309	0.9400
C102—C103	1.391 (9)	C310—C311	1.367 (12)
C102—H102	0.9400	C310—H310	0.9400
C103—C104	1.355 (10)	C311—C312	1.396 (10)
C103—H103	0.9400	C311—H311	0.9400
C104—C105	1.359 (9)	C312—H312	0.9400
C104—H104	0.9400	C401—C402	1.358 (9)
C105—C106	1.390 (9)	C401—C406	1.371 (10)
C105—H105	0.9400	C402—C403	1.373 (11)
C106—H106	0.9400	C402—H402	0.9400
C107—C108	1.381 (8)	C403—C404	1.317 (13)
C107—C112	1.387 (8)	C403—H403	0.9400
C108—C109	1.396 (9)	C404—C405	1.390 (13)
C108—H108	0.9400	C404—H404	0.9400
C109—C110	1.378 (10)	C405—C406	1.389 (11)
C109—H109	0.9400	C405—H405	0.9400
C110—C111	1.364 (10)	C406—H406	0.9400
C110—H110	0.9400	C407—C408	1.369 (9)
C111—C112	1.387 (9)	C407—C412	1.376 (10)
C111—H111	0.9400	C408—C409	1.371 (11)
C112—H112	0.9400	C408—H408	0.9400
C201—C202	1.385 (9)	C409—C410	1.337 (13)
C201—C206	1.385 (9)	C409—H409	0.9400
C202—C203	1.373 (10)	C410—C411	1.342 (14)
C202—H202	0.9400	C410—H410	0.9400
C203—C204	1.377 (12)	C411—C412	1.380 (11)
C203—H203	0.9400	C411—H411	0.9400
C204—C205	1.382 (12)	C412—H412	0.9400
C204—H204	0.9400	O1—C4	1.197 (11)
C205—C206	1.390 (10)	C4—C5	1.450 (15)
C205—H205	0.9400	C4—C6A	1.47 (3)
C206—H206	0.9400	C4—C6	1.51 (2)
C207—C208	1.363 (9)	C6—C6A	1.56 (4)
C207—C212	1.387 (9)		
			
C1—Ir1—P4	84.28 (16)	C204—C205—H205	120.8
C1—Ir1—P1	89.23 (16)	C206—C205—H205	120.8
P4—Ir1—P1	173.09 (5)	C201—C206—C205	121.0 (7)
C1—Ir1—Cl1	175.48 (16)	C201—C206—H206	119.5
P4—Ir1—Cl1	92.62 (6)	C205—C206—H206	119.5
P1—Ir1—Cl1	93.72 (5)	C208—C207—C212	119.8 (6)
C1—Ir1—Cl2	88.52 (15)	C208—C207—P2	118.6 (5)
P4—Ir1—Cl2	89.12 (5)	C212—C207—P2	121.6 (5)
P1—Ir1—Cl2	93.08 (5)	C207—C208—C209	121.1 (8)
Cl1—Ir1—Cl2	94.73 (5)	C207—C208—H208	119.4
C1—Ir1—H1	90 (2)	C209—C208—H208	119.4
P4—Ir1—H1	83 (2)	C210—C209—C208	117.9 (9)
P1—Ir1—H1	94 (2)	C210—C209—H209	121.0
Cl1—Ir1—H1	87 (2)	C208—C209—H209	121.0
Cl2—Ir1—H1	173 (2)	C211—C210—C209	121.6 (8)
C101—P1—C107	104.0 (3)	C211—C210—H210	119.2
C101—P1—C2	103.4 (3)	C209—C210—H210	119.2
C107—P1—C2	104.3 (3)	C210—C211—C212	120.0 (9)
C101—P1—Ir1	119.99 (19)	C210—C211—H211	120.0
C107—P1—Ir1	117.0 (2)	C212—C211—H211	120.0
C2—P1—Ir1	106.4 (2)	C211—C212—C207	119.6 (8)
C1—P2—C201	117.4 (3)	C211—C212—H212	120.2
C1—P2—C2	106.8 (3)	C207—C212—H212	120.2
C201—P2—C2	108.3 (3)	C302—C301—C306	118.2 (6)
C1—P2—C207	114.6 (3)	C302—C301—P3	122.0 (5)
C201—P2—C207	104.5 (3)	C306—C301—P3	119.6 (5)
C2—P2—C207	104.5 (3)	C301—C302—C303	120.5 (6)
C1—P3—C307	113.3 (3)	C301—C302—H302	119.8
C1—P3—C301	122.0 (3)	C303—C302—H302	119.8
C307—P3—C301	104.0 (3)	C304—C303—C302	120.0 (6)
C1—P3—C3	106.2 (3)	C304—C303—H303	120.0
C307—P3—C3	109.0 (3)	C302—C303—H303	120.0
C301—P3—C3	101.4 (3)	C305—C304—C303	120.0 (6)
C401—P4—C3	104.7 (3)	C305—C304—H304	120.0
C401—P4—C407	101.7 (3)	C303—C304—H304	120.0
C3—P4—C407	103.8 (3)	C304—C305—C306	120.3 (6)
C401—P4—Ir1	119.7 (2)	C304—C305—H305	119.8
C3—P4—Ir1	106.1 (2)	C306—C305—H305	119.8
C407—P4—Ir1	119.0 (2)	C305—C306—C301	120.9 (6)
P3—C1—P2	127.1 (3)	C305—C306—H306	119.5
P3—C1—Ir1	120.4 (3)	C301—C306—H306	119.5
P2—C1—Ir1	112.3 (3)	C312—C307—C308	117.3 (6)
P2—C2—P1	105.4 (3)	C312—C307—P3	123.8 (5)
P2—C2—H2A	110.7	C308—C307—P3	118.6 (5)
P1—C2—H2A	110.7	C309—C308—C307	121.0 (7)
P2—C2—H2B	110.7	C309—C308—H308	119.5
P1—C2—H2B	110.7	C307—C308—H308	119.5
H2A—C2—H2B	108.8	C310—C309—C308	120.0 (8)
P3—C3—P4	110.0 (3)	C310—C309—H309	120.0
P3—C3—H3A	109.7	C308—C309—H309	120.0
P4—C3—H3A	109.7	C309—C310—C311	121.8 (8)
P3—C3—H3B	109.7	C309—C310—H310	119.1
P4—C3—H3B	109.7	C311—C310—H310	119.1
H3A—C3—H3B	108.2	C310—C311—C312	117.4 (8)
C102—C101—C106	118.0 (6)	C310—C311—H311	121.3
C102—C101—P1	118.7 (4)	C312—C311—H311	121.3
C106—C101—P1	123.2 (4)	C307—C312—C311	122.4 (7)
C101—C102—C103	120.1 (6)	C307—C312—H312	118.8
C101—C102—H102	119.9	C311—C312—H312	118.8
C103—C102—H102	119.9	C402—C401—C406	116.9 (7)
C104—C103—C102	120.8 (6)	C402—C401—P4	122.3 (5)
C104—C103—H103	119.6	C406—C401—P4	120.0 (6)
C102—C103—H103	119.6	C401—C402—C403	121.6 (8)
C103—C104—C105	120.4 (6)	C401—C402—H402	119.2
C103—C104—H104	119.8	C403—C402—H402	119.2
C105—C104—H104	119.8	C404—C403—C402	121.9 (9)
C104—C105—C106	119.5 (6)	C404—C403—H403	119.0
C104—C105—H105	120.3	C402—C403—H403	119.0
C106—C105—H105	120.3	C403—C404—C405	118.3 (8)
C101—C106—C105	121.1 (6)	C403—C404—H404	120.8
C101—C106—H106	119.4	C405—C404—H404	120.8
C105—C106—H106	119.4	C406—C405—C404	119.5 (8)
C108—C107—C112	118.7 (5)	C406—C405—H405	120.3
C108—C107—P1	119.4 (5)	C404—C405—H405	120.3
C112—C107—P1	122.0 (4)	C401—C406—C405	121.1 (8)
C107—C108—C109	120.5 (6)	C401—C406—H406	119.5
C107—C108—H108	119.7	C405—C406—H406	119.5
C109—C108—H108	119.7	C408—C407—C412	117.4 (7)
C110—C109—C108	119.8 (7)	C408—C407—P4	120.8 (6)
C110—C109—H109	120.1	C412—C407—P4	121.7 (6)
C108—C109—H109	120.1	C407—C408—C409	120.6 (8)
C111—C110—C109	119.9 (6)	C407—C408—H408	119.7
C111—C110—H110	120.0	C409—C408—H408	119.7
C109—C110—H110	120.0	C410—C409—C408	121.2 (9)
C110—C111—C112	120.5 (6)	C410—C409—H409	119.4
C110—C111—H111	119.7	C408—C409—H409	119.4
C112—C111—H111	119.7	C409—C410—C411	119.7 (9)
C111—C112—C107	120.5 (6)	C409—C410—H410	120.1
C111—C112—H112	119.8	C411—C410—H410	120.1
C107—C112—H112	119.8	C410—C411—C412	120.2 (9)
C202—C201—C206	119.2 (6)	C410—C411—H411	119.9
C202—C201—P2	121.8 (5)	C412—C411—H411	119.9
C206—C201—P2	119.0 (5)	C407—C412—C411	120.8 (8)
C203—C202—C201	120.3 (7)	C407—C412—H412	119.6
C203—C202—H202	119.9	C411—C412—H412	119.6
C201—C202—H202	119.9	O1—C4—C5	125.7 (12)
C202—C203—C204	120.0 (8)	O1—C4—C6A	111.0 (16)
C202—C203—H203	120.0	C5—C4—C6A	115.0 (17)
C204—C203—H203	120.0	O1—C4—C6	118.2 (13)
C203—C204—C205	121.1 (8)	C5—C4—C6	107.6 (15)
C203—C204—H204	119.4	C6A—C4—C6	62.8 (16)
C205—C204—H204	119.4	C4—C6—C6A	57.2 (14)
C204—C205—C206	118.4 (8)	C4—C6A—C6	60.0 (15)

### Dichloridohydrido(1,1,3,3,5,5,7,7-octaphenyl-1,5λ5,7-triphospha-3-phosphoniahept-4-en-4-yl)iridium(III) acetone monosolvate (2) Hydrogen-bond geometry (Å, º)

**Table:**

*D*—H···*A*	*D*—H	H···*A*	*D*···*A*	*D*—H···*A*
C112—H112···Cl1^i^	0.94	2.73	3.601 (6)	154
C3—H3*B*···O1	0.98	2.48	3.435 (10)	163

Symmetry code: (i) −*x*+1/2, *y*+1/2, −*z*+3/2.

### Dichloridohydrido(1,1,3,3,5,5,7,7-octaphenyl-1,7-diphospha-3,5-diphosphoniaheptan-4-yl)iridium(III) chloride pentahydrate (3) Crystal data

**Table:**

[Ir(C_51_H_45_P_4_)ClH]Cl·5H_2_O	*F*(000) = 2360
*M**_r_* = 1171.38	*D*_x_ = 1.429 Mg m^−^^3^
Monoclinic, *P*2_1_/*c*	Mo *K*α radiation, λ = 0.71073 Å
*a* = 12.6532 (8) Å	Cell parameters from 9971 reflections
*b* = 21.8847 (12) Å	θ = 2.7–25.9°
*c* = 19.9228 (12) Å	µ = 2.76 mm^−^^1^
β = 99.381 (2)°	*T* = 203 K
*V* = 5443.1 (6) Å^3^	Prism, colourless
*Z* = 4	0.17 × 0.12 × 0.09 mm

### Dichloridohydrido(1,1,3,3,5,5,7,7-octaphenyl-1,7-diphospha-3,5-diphosphoniaheptan-4-yl)iridium(III) chloride pentahydrate (3) Data collection

**Table:**

Bruker D8 QUEST PHOTON 100 diffractometer	10577 independent reflections
Radiation source: Incoatec Microfocus	9522 reflections with *I* > 2σ(*I*)
Multi layered optics monochromator	*R*_int_ = 0.031
Detector resolution: 10.4 pixels mm^-1^	θ_max_ = 25.9°, θ_min_ = 2.1°
φ and ω scans	*h* = −15→14
Absorption correction: multi-scan (SADABS; Bruker, 2015)	*k* = −26→26
*T*_min_ = 0.691, *T*_max_ = 0.801	*l* = −24→24
104100 measured reflections	

### Dichloridohydrido(1,1,3,3,5,5,7,7-octaphenyl-1,7-diphospha-3,5-diphosphoniaheptan-4-yl)iridium(III) chloride pentahydrate (3) Refinement

**Table:**

Refinement on *F*^2^	Hydrogen site location: mixed
Least-squares matrix: full	H atoms treated by a mixture of independent and constrained refinement
*R*[*F*^2^ > 2σ(*F*^2^)] = 0.032	*w* = 1/[σ^2^(*F*_o_^2^) + (0.0524*P*)^2^ + 9.2728*P*] where *P* = (*F*_o_^2^ + 2*F*_c_^2^)/3
*wR*(*F*^2^) = 0.095	(Δ/σ)_max_ = 0.003
*S* = 1.09	Δρ_max_ = 1.27 e Å^−^^3^
10577 reflections	Δρ_min_ = −0.86 e Å^−^^3^
574 parameters	Extinction correction: (SHELXL-2014/7; Sheldrick, 2015b), Fc^*^=kFc[1+0.001xFc^2^λ^3^/sin(2θ)]^-1/4^
0 restraints	Extinction coefficient: 0.00056 (9)

### Dichloridohydrido(1,1,3,3,5,5,7,7-octaphenyl-1,7-diphospha-3,5-diphosphoniaheptan-4-yl)iridium(III) chloride pentahydrate (3) Special details

**Table:**

Geometry. All esds (except the esd in the dihedral angle between two l.s. planes) are estimated using the full covariance matrix. The cell esds are taken into account individually in the estimation of esds in distances, angles and torsion angles; correlations between esds in cell parameters are only used when they are defined by crystal symmetry. An approximate (isotropic) treatment of cell esds is used for estimating esds involving l.s. planes.
Refinement. Positional Disorder of the Anion Cl3:Cl3A in ratio 2:1. Hydrogen atoms at C1 (H1A) and Ir1 (H1) were found and refined regularly with isotropic displacement parameters. The water solvent molecules show higher temperature factors and would be slightly disordered, but this disorder was not solved, and therefore the oxygen atoms (O5 and O6 with half occupancy) were refined isotropically and their hydrogen atoms were omitted.

### Dichloridohydrido(1,1,3,3,5,5,7,7-octaphenyl-1,7-diphospha-3,5-diphosphoniaheptan-4-yl)iridium(III) chloride pentahydrate (3) Fractional atomic coordinates and isotropic or equivalent isotropic displacement parameters (Å^2^)

**Table:**

	*x*	*y*	*z*	*U*_iso_*/*U*_eq_	Occ. (<1)
Ir1	0.18691 (2)	0.09326 (2)	0.82207 (2)	0.03005 (7)	
H1	0.207 (4)	0.069 (2)	0.757 (2)	0.049 (12)*	
P1	0.00638 (8)	0.07642 (5)	0.78743 (5)	0.0349 (2)	
P2	0.06530 (9)	0.17799 (4)	0.70390 (5)	0.0357 (2)	
P3	0.28773 (9)	0.22466 (4)	0.77953 (5)	0.0362 (2)	
P4	0.36829 (8)	0.10629 (5)	0.83855 (5)	0.0345 (2)	
Cl1	0.21275 (9)	−0.00814 (5)	0.86833 (6)	0.0517 (3)	
Cl2	0.15051 (8)	0.13129 (5)	0.93346 (5)	0.0419 (2)	
Cl3	−0.0093 (4)	0.2435 (2)	0.8764 (2)	0.1427 (13)	0.67
Cl3A	0.4940 (10)	0.1089 (7)	0.6444 (5)	0.195 (5)	0.33
C1	0.1651 (3)	0.18280 (16)	0.77996 (17)	0.0318 (7)	
H1A	0.129 (3)	0.2086 (19)	0.810 (2)	0.037 (10)*	
C2	−0.0442 (3)	0.14516 (19)	0.7392 (2)	0.0433 (9)	
H2A	−0.1028	0.1342	0.7027	0.052*	
H2B	−0.0713	0.1746	0.7693	0.052*	
C3	0.3948 (3)	0.17048 (19)	0.7832 (2)	0.0413 (9)	
H3A	0.4631	0.1900	0.8016	0.050*	
H3B	0.3992	0.1554	0.7374	0.050*	
C101	−0.0786 (3)	0.0675 (2)	0.8515 (2)	0.0417 (9)	
C102	−0.0637 (4)	0.0159 (2)	0.8920 (2)	0.0538 (12)	
H102	−0.0135	−0.0138	0.8841	0.065*	
C103	−0.1229 (4)	0.0076 (3)	0.9446 (2)	0.0699 (17)	
H103	−0.1132	−0.0279	0.9713	0.084*	
C104	−0.1940 (4)	0.0504 (3)	0.9574 (2)	0.0702 (17)	
H104	−0.2345	0.0443	0.9924	0.084*	
C105	−0.2071 (5)	0.1026 (3)	0.9193 (3)	0.0684 (15)	
H105	−0.2549	0.1328	0.9295	0.082*	
C106	−0.1512 (4)	0.1117 (3)	0.8658 (3)	0.0552 (11)	
H106	−0.1622	0.1474	0.8394	0.066*	
C107	−0.0332 (4)	0.0131 (2)	0.7293 (2)	0.0460 (10)	
C108	−0.1415 (5)	0.0046 (3)	0.7037 (3)	0.0773 (18)	
H108	−0.1930	0.0317	0.7155	0.093*	
C109	−0.1725 (6)	−0.0445 (4)	0.6602 (3)	0.097 (3)	
H109	−0.2454	−0.0508	0.6429	0.116*	
C110	−0.0981 (8)	−0.0831 (3)	0.6429 (3)	0.092 (3)	
H110	−0.1199	−0.1160	0.6136	0.110*	
C111	0.0086 (7)	−0.0751 (3)	0.6674 (3)	0.083 (2)	
H111	0.0595	−0.1025	0.6552	0.100*	
C112	0.0410 (5)	−0.0264 (2)	0.7103 (3)	0.0614 (13)	
H112	0.1143	−0.0203	0.7265	0.074*	
C201	0.0157 (4)	0.24892 (18)	0.66472 (19)	0.0401 (9)	
C202	−0.0364 (5)	0.2893 (2)	0.7017 (2)	0.0569 (13)	
H202	−0.0418	0.2808	0.7473	0.068*	
C203	−0.0808 (5)	0.3425 (2)	0.6709 (3)	0.0658 (15)	
H203	−0.1165	0.3701	0.6955	0.079*	
C204	−0.0723 (5)	0.3545 (2)	0.6043 (2)	0.0604 (13)	
H204	−0.1045	0.3898	0.5832	0.072*	
C205	−0.0173 (4)	0.3157 (2)	0.5681 (2)	0.0533 (12)	
H205	−0.0095	0.3254	0.5232	0.064*	
C206	0.0265 (4)	0.26226 (19)	0.5978 (2)	0.0454 (10)	
H206	0.0633	0.2353	0.5730	0.055*	
C207	0.0977 (4)	0.12959 (18)	0.63756 (19)	0.0421 (9)	
C208	0.2004 (4)	0.1117 (2)	0.6323 (2)	0.0487 (10)	
H208	0.2580	0.1248	0.6650	0.058*	
C209	0.2196 (5)	0.0745 (2)	0.5792 (3)	0.0637 (14)	
H209	0.2897	0.0617	0.5767	0.076*	
C210	0.1370 (6)	0.0567 (3)	0.5309 (3)	0.0749 (18)	
H210	0.1505	0.0317	0.4948	0.090*	
C211	0.0332 (6)	0.0749 (3)	0.5342 (3)	0.0751 (18)	
H211	−0.0235	0.0626	0.5003	0.090*	
C212	0.0134 (5)	0.1113 (2)	0.5876 (2)	0.0597 (13)	
H212	−0.0570	0.1236	0.5902	0.072*	
C301	0.2843 (4)	0.27525 (19)	0.7085 (2)	0.0479 (11)	
C302	0.2267 (4)	0.3299 (2)	0.7114 (2)	0.0550 (12)	
H302	0.1945	0.3385	0.7496	0.066*	
C303	0.2174 (5)	0.3711 (2)	0.6578 (3)	0.0686 (16)	
H303	0.1779	0.4074	0.6591	0.082*	
C304	0.2668 (5)	0.3584 (3)	0.6030 (3)	0.0749 (18)	
H304	0.2616	0.3867	0.5671	0.090*	
C305	0.3234 (6)	0.3053 (3)	0.5992 (2)	0.0759 (19)	
H305	0.3560	0.2976	0.5609	0.091*	
C306	0.3330 (5)	0.2625 (3)	0.6525 (2)	0.0625 (14)	
H306	0.3715	0.2261	0.6503	0.075*	
C307	0.3189 (4)	0.27214 (17)	0.85395 (19)	0.0407 (9)	
C308	0.2649 (4)	0.2706 (2)	0.9078 (2)	0.0485 (10)	
H308	0.2063	0.2441	0.9073	0.058*	
C309	0.2969 (5)	0.3086 (2)	0.9639 (2)	0.0621 (13)	
H309	0.2598	0.3075	1.0011	0.075*	
C310	0.3818 (5)	0.3472 (2)	0.9649 (3)	0.0694 (16)	
H310	0.4024	0.3729	1.0027	0.083*	
C311	0.4371 (5)	0.3488 (3)	0.9111 (3)	0.0769 (17)	
H311	0.4958	0.3753	0.9123	0.092*	
C312	0.4066 (5)	0.3115 (2)	0.8551 (3)	0.0641 (14)	
H312	0.4444	0.3126	0.8182	0.077*	
C401	0.4481 (3)	0.0466 (2)	0.8070 (2)	0.0440 (9)	
C402	0.4020 (5)	0.0026 (4)	0.7623 (4)	0.107 (3)	
H402	0.3271	0.0009	0.7497	0.129*	
C403	0.4670 (6)	−0.0394 (4)	0.7359 (5)	0.140 (4)	
H403	0.4355	−0.0688	0.7045	0.168*	
C404	0.5742 (5)	−0.0386 (3)	0.7545 (3)	0.086 (2)	
H404	0.6171	−0.0665	0.7352	0.103*	
C405	0.6192 (4)	0.0022 (3)	0.8006 (3)	0.0633 (13)	
H405	0.6938	0.0017	0.8151	0.076*	
C406	0.5570 (4)	0.0447 (2)	0.8268 (2)	0.0506 (11)	
H406	0.5898	0.0731	0.8590	0.061*	
C407	0.4430 (3)	0.1214 (2)	0.9230 (2)	0.0412 (9)	
C408	0.4308 (4)	0.0783 (2)	0.9726 (2)	0.0531 (11)	
H408	0.3806	0.0465	0.9631	0.064*	
C409	0.4939 (5)	0.0830 (3)	1.0365 (3)	0.0684 (15)	
H409	0.4875	0.0532	1.0696	0.082*	
C410	0.5643 (5)	0.1295 (3)	1.0519 (3)	0.0698 (15)	
H410	0.6059	0.1319	1.0955	0.084*	
C411	0.5752 (4)	0.1733 (3)	1.0040 (3)	0.0660 (14)	
H411	0.6235	0.2058	1.0148	0.079*	
C412	0.5140 (4)	0.1690 (2)	0.9391 (2)	0.0538 (11)	
H412	0.5213	0.1988	0.9062	0.065*	
O1	0.6533 (8)	0.2455 (5)	0.7966 (5)	0.189 (3)*	
O2	0.7918 (10)	0.2878 (6)	0.9223 (6)	0.244 (5)*	
O3	0.7393 (15)	0.4042 (7)	0.9328 (9)	0.318 (8)*	
O4	0.6081 (13)	0.4713 (7)	1.0118 (8)	0.308 (7)*	
O5	0.6616 (17)	0.1673 (10)	0.6823 (11)	0.209 (8)*	0.5
O6	0.7444 (17)	0.1702 (10)	0.5728 (10)	0.206 (8)*	0.5

### Dichloridohydrido(1,1,3,3,5,5,7,7-octaphenyl-1,7-diphospha-3,5-diphosphoniaheptan-4-yl)iridium(III) chloride pentahydrate (3) Atomic displacement parameters (Å^2^)

**Table:**

	*U*^11^	*U*^22^	*U*^33^	*U*^12^	*U*^13^	*U*^23^
Ir1	0.03410 (10)	0.02785 (10)	0.02848 (9)	−0.00002 (5)	0.00597 (6)	0.00535 (5)
P1	0.0370 (5)	0.0372 (5)	0.0302 (5)	−0.0035 (4)	0.0040 (4)	0.0090 (4)
P2	0.0520 (6)	0.0282 (5)	0.0256 (4)	−0.0013 (4)	0.0024 (4)	0.0044 (4)
P3	0.0544 (6)	0.0296 (5)	0.0247 (4)	−0.0082 (4)	0.0073 (4)	0.0010 (4)
P4	0.0346 (5)	0.0351 (5)	0.0342 (5)	0.0006 (4)	0.0068 (4)	−0.0025 (4)
Cl1	0.0568 (6)	0.0352 (5)	0.0591 (6)	−0.0004 (4)	−0.0029 (5)	0.0179 (5)
Cl2	0.0489 (6)	0.0501 (6)	0.0272 (4)	−0.0017 (4)	0.0077 (4)	0.0048 (4)
Cl3	0.140 (3)	0.158 (4)	0.133 (3)	0.002 (3)	0.029 (2)	0.016 (3)
Cl3A	0.191 (10)	0.309 (15)	0.086 (5)	−0.005 (10)	0.019 (6)	0.012 (7)
C1	0.043 (2)	0.0296 (18)	0.0219 (16)	0.0008 (15)	0.0040 (14)	0.0009 (13)
C2	0.045 (2)	0.044 (2)	0.039 (2)	−0.0006 (18)	0.0007 (17)	0.0144 (17)
C3	0.046 (2)	0.044 (2)	0.037 (2)	−0.0097 (18)	0.0144 (17)	−0.0045 (17)
C101	0.034 (2)	0.055 (3)	0.036 (2)	−0.0068 (18)	0.0052 (16)	0.0074 (18)
C102	0.046 (2)	0.068 (3)	0.048 (2)	−0.007 (2)	0.008 (2)	0.021 (2)
C103	0.056 (3)	0.111 (5)	0.039 (2)	−0.032 (3)	−0.001 (2)	0.028 (3)
C104	0.049 (3)	0.125 (5)	0.038 (2)	−0.035 (3)	0.011 (2)	−0.009 (3)
C105	0.055 (3)	0.090 (4)	0.065 (3)	−0.020 (3)	0.024 (3)	−0.021 (3)
C106	0.045 (3)	0.063 (3)	0.060 (3)	−0.007 (2)	0.014 (2)	−0.002 (2)
C107	0.059 (3)	0.048 (2)	0.0298 (19)	−0.019 (2)	0.0020 (18)	0.0092 (17)
C108	0.069 (4)	0.116 (5)	0.048 (3)	−0.041 (3)	0.015 (3)	−0.017 (3)
C109	0.100 (5)	0.143 (7)	0.050 (3)	−0.072 (5)	0.018 (3)	−0.033 (4)
C110	0.156 (8)	0.081 (4)	0.036 (3)	−0.058 (5)	0.007 (4)	−0.007 (3)
C111	0.135 (6)	0.051 (3)	0.055 (3)	−0.003 (3)	−0.015 (4)	−0.007 (3)
C112	0.080 (4)	0.043 (3)	0.055 (3)	0.002 (2)	−0.008 (3)	−0.004 (2)
C201	0.059 (3)	0.0304 (19)	0.0277 (18)	0.0008 (17)	−0.0033 (17)	0.0051 (15)
C202	0.094 (4)	0.043 (2)	0.034 (2)	0.013 (2)	0.008 (2)	0.0030 (18)
C203	0.104 (4)	0.038 (2)	0.054 (3)	0.017 (3)	0.009 (3)	0.001 (2)
C204	0.092 (4)	0.037 (2)	0.045 (3)	0.008 (2)	−0.011 (2)	0.0072 (19)
C205	0.081 (3)	0.043 (2)	0.032 (2)	−0.002 (2)	−0.002 (2)	0.0135 (18)
C206	0.066 (3)	0.037 (2)	0.031 (2)	−0.0002 (19)	0.0006 (19)	0.0042 (16)
C207	0.065 (3)	0.0310 (19)	0.0299 (19)	−0.0052 (18)	0.0053 (18)	0.0006 (15)
C208	0.072 (3)	0.044 (2)	0.032 (2)	−0.005 (2)	0.013 (2)	−0.0006 (18)
C209	0.098 (4)	0.052 (3)	0.047 (3)	−0.003 (3)	0.030 (3)	−0.004 (2)
C210	0.130 (6)	0.054 (3)	0.043 (3)	−0.007 (3)	0.020 (3)	−0.011 (2)
C211	0.119 (6)	0.056 (3)	0.044 (3)	−0.016 (3)	−0.006 (3)	−0.015 (2)
C212	0.083 (4)	0.048 (3)	0.043 (3)	−0.007 (2)	−0.005 (2)	−0.006 (2)
C301	0.072 (3)	0.041 (2)	0.0296 (19)	−0.024 (2)	0.0041 (19)	0.0030 (17)
C302	0.075 (3)	0.042 (2)	0.044 (2)	−0.017 (2)	−0.004 (2)	0.0108 (19)
C303	0.093 (4)	0.050 (3)	0.052 (3)	−0.027 (3)	−0.019 (3)	0.019 (2)
C304	0.106 (5)	0.069 (4)	0.042 (3)	−0.045 (3)	−0.008 (3)	0.016 (2)
C305	0.111 (5)	0.082 (4)	0.036 (2)	−0.049 (4)	0.017 (3)	−0.001 (3)
C306	0.092 (4)	0.063 (3)	0.035 (2)	−0.032 (3)	0.019 (2)	−0.001 (2)
C307	0.057 (3)	0.0311 (19)	0.0310 (19)	−0.0018 (17)	−0.0016 (17)	−0.0004 (15)
C308	0.071 (3)	0.040 (2)	0.034 (2)	−0.002 (2)	0.008 (2)	−0.0039 (17)
C309	0.096 (4)	0.053 (3)	0.037 (2)	0.000 (3)	0.009 (2)	−0.009 (2)
C310	0.102 (4)	0.056 (3)	0.043 (3)	−0.008 (3)	−0.007 (3)	−0.018 (2)
C311	0.097 (4)	0.063 (3)	0.066 (4)	−0.032 (3)	0.000 (3)	−0.019 (3)
C312	0.081 (4)	0.062 (3)	0.050 (3)	−0.026 (3)	0.012 (3)	−0.011 (2)
C401	0.041 (2)	0.047 (2)	0.044 (2)	0.0066 (18)	0.0057 (18)	−0.0071 (18)
C402	0.057 (4)	0.120 (6)	0.132 (6)	0.031 (4)	−0.023 (4)	−0.085 (5)
C403	0.081 (5)	0.151 (8)	0.172 (8)	0.038 (5)	−0.030 (5)	−0.123 (7)
C404	0.064 (4)	0.093 (5)	0.095 (4)	0.035 (3)	0.001 (3)	−0.040 (4)
C405	0.046 (3)	0.070 (3)	0.072 (3)	0.017 (2)	0.006 (2)	−0.013 (3)
C406	0.041 (2)	0.051 (3)	0.059 (3)	0.0038 (19)	0.006 (2)	−0.010 (2)
C407	0.039 (2)	0.046 (2)	0.039 (2)	0.0063 (17)	0.0071 (17)	−0.0048 (17)
C408	0.063 (3)	0.057 (3)	0.040 (2)	0.002 (2)	0.009 (2)	0.000 (2)
C409	0.084 (4)	0.077 (4)	0.043 (3)	0.019 (3)	0.006 (3)	0.006 (2)
C410	0.066 (3)	0.089 (4)	0.047 (3)	0.016 (3)	−0.010 (2)	−0.013 (3)
C411	0.058 (3)	0.080 (4)	0.056 (3)	−0.003 (3)	−0.005 (2)	−0.016 (3)
C412	0.052 (3)	0.059 (3)	0.048 (3)	−0.005 (2)	0.003 (2)	−0.008 (2)

### Dichloridohydrido(1,1,3,3,5,5,7,7-octaphenyl-1,7-diphospha-3,5-diphosphoniaheptan-4-yl)iridium(III) chloride pentahydrate (3) Geometric parameters (Å, º)

**Table:**

Ir1—C1	2.132 (4)	C206—H206	0.9400
Ir1—P4	2.2827 (10)	C207—C208	1.377 (7)
Ir1—P1	2.3056 (10)	C207—C212	1.394 (6)
Ir1—Cl1	2.4047 (10)	C208—C209	1.386 (7)
Ir1—Cl2	2.4824 (10)	C208—H208	0.9400
Ir1—H1	1.46 (5)	C209—C210	1.357 (9)
P1—C101	1.809 (4)	C209—H209	0.9400
P1—C107	1.823 (4)	C210—C211	1.385 (10)
P1—C2	1.843 (4)	C210—H210	0.9400
P2—C207	1.793 (4)	C211—C212	1.385 (8)
P2—C2	1.802 (4)	C211—H211	0.9400
P2—C201	1.803 (4)	C212—H212	0.9400
P2—C1	1.811 (4)	C301—C306	1.388 (7)
P3—C301	1.791 (4)	C301—C302	1.406 (7)
P3—C3	1.793 (4)	C302—C303	1.389 (6)
P3—C307	1.801 (4)	C302—H302	0.9400
P3—C1	1.803 (4)	C303—C304	1.373 (9)
P4—C407	1.821 (4)	C303—H303	0.9400
P4—C401	1.824 (4)	C304—C305	1.373 (9)
P4—C3	1.850 (4)	C304—H304	0.9400
C1—H1A	0.98 (4)	C305—C306	1.406 (7)
C2—H2A	0.9800	C305—H305	0.9400
C2—H2B	0.9800	C306—H306	0.9400
C3—H3A	0.9800	C307—C308	1.364 (6)
C3—H3B	0.9800	C307—C312	1.403 (7)
C101—C102	1.383 (6)	C308—C309	1.398 (6)
C101—C106	1.395 (7)	C308—H308	0.9400
C102—C103	1.396 (7)	C309—C310	1.364 (8)
C102—H102	0.9400	C309—H309	0.9400
C103—C104	1.351 (9)	C310—C311	1.373 (9)
C103—H103	0.9400	C310—H310	0.9400
C104—C105	1.365 (9)	C311—C312	1.387 (7)
C104—H104	0.9400	C311—H311	0.9400
C105—C106	1.386 (7)	C312—H312	0.9400
C105—H105	0.9400	C401—C406	1.370 (6)
C106—H106	0.9400	C401—C402	1.376 (7)
C107—C112	1.374 (7)	C402—C403	1.393 (8)
C107—C108	1.394 (7)	C402—H402	0.9400
C108—C109	1.397 (9)	C403—C404	1.346 (9)
C108—H108	0.9400	C403—H403	0.9400
C109—C110	1.350 (11)	C404—C405	1.339 (8)
C109—H109	0.9400	C404—H404	0.9400
C110—C111	1.370 (11)	C405—C406	1.377 (6)
C110—H110	0.9400	C405—H405	0.9400
C111—C112	1.386 (7)	C406—H406	0.9400
C111—H111	0.9400	C407—C412	1.379 (6)
C112—H112	0.9400	C407—C408	1.394 (6)
C201—C202	1.385 (6)	C408—C409	1.390 (7)
C201—C206	1.394 (5)	C408—H408	0.9400
C202—C203	1.391 (6)	C409—C410	1.357 (9)
C202—H202	0.9400	C409—H409	0.9400
C203—C204	1.374 (7)	C410—C411	1.374 (8)
C203—H203	0.9400	C410—H410	0.9400
C204—C205	1.375 (7)	C411—C412	1.396 (7)
C204—H204	0.9400	C411—H411	0.9400
C205—C206	1.385 (6)	C412—H412	0.9400
C205—H205	0.9400		
			
C1—Ir1—P4	90.24 (11)	C202—C203—H203	120.1
C1—Ir1—P1	88.33 (11)	C203—C204—C205	120.9 (4)
P4—Ir1—P1	170.68 (4)	C203—C204—H204	119.5
C1—Ir1—Cl1	179.22 (11)	C205—C204—H204	119.5
P4—Ir1—Cl1	89.39 (4)	C204—C205—C206	120.0 (4)
P1—Ir1—Cl1	91.92 (4)	C204—C205—H205	120.0
C1—Ir1—Cl2	90.73 (10)	C206—C205—H205	120.0
P4—Ir1—Cl2	99.10 (3)	C205—C206—C201	119.4 (4)
P1—Ir1—Cl2	90.12 (4)	C205—C206—H206	120.3
Cl1—Ir1—Cl2	90.01 (4)	C201—C206—H206	120.3
C1—Ir1—H1	90.5 (18)	C208—C207—C212	119.1 (4)
P4—Ir1—H1	81.7 (18)	C208—C207—P2	123.8 (3)
P1—Ir1—H1	89.1 (18)	C212—C207—P2	117.1 (4)
Cl1—Ir1—H1	88.8 (18)	C207—C208—C209	120.6 (5)
Cl2—Ir1—H1	178.5 (18)	C207—C208—H208	119.7
C101—P1—C107	103.5 (2)	C209—C208—H208	119.7
C101—P1—C2	105.1 (2)	C210—C209—C208	120.0 (6)
C107—P1—C2	104.6 (2)	C210—C209—H209	120.0
C101—P1—Ir1	118.69 (13)	C208—C209—H209	120.0
C107—P1—Ir1	117.89 (16)	C209—C210—C211	120.6 (5)
C2—P1—Ir1	105.58 (14)	C209—C210—H210	119.7
C207—P2—C2	109.6 (2)	C211—C210—H210	119.7
C207—P2—C201	107.14 (18)	C210—C211—C212	119.6 (5)
C2—P2—C201	106.1 (2)	C210—C211—H211	120.2
C207—P2—C1	116.04 (19)	C212—C211—H211	120.2
C2—P2—C1	99.93 (18)	C211—C212—C207	120.0 (6)
C201—P2—C1	117.26 (18)	C211—C212—H212	120.0
C301—P3—C3	111.2 (2)	C207—C212—H212	120.0
C301—P3—C307	105.52 (19)	C306—C301—C302	120.3 (4)
C3—P3—C307	106.6 (2)	C306—C301—P3	123.6 (4)
C301—P3—C1	114.05 (19)	C302—C301—P3	116.1 (3)
C3—P3—C1	107.99 (18)	C303—C302—C301	120.1 (5)
C307—P3—C1	111.25 (19)	C303—C302—H302	120.0
C407—P4—C401	102.41 (19)	C301—C302—H302	120.0
C407—P4—C3	107.2 (2)	C304—C303—C302	119.1 (6)
C401—P4—C3	100.0 (2)	C304—C303—H303	120.4
C407—P4—Ir1	120.94 (14)	C302—C303—H303	120.4
C401—P4—Ir1	117.46 (15)	C305—C304—C303	121.6 (5)
C3—P4—Ir1	106.55 (14)	C305—C304—H304	119.2
P3—C1—P2	120.60 (19)	C303—C304—H304	119.2
P3—C1—Ir1	114.38 (19)	C304—C305—C306	120.3 (5)
P2—C1—Ir1	107.68 (17)	C304—C305—H305	119.9
P3—C1—H1A	101 (2)	C306—C305—H305	119.9
P2—C1—H1A	102 (2)	C301—C306—C305	118.6 (6)
Ir1—C1—H1A	110 (2)	C301—C306—H306	120.7
P2—C2—P1	107.8 (2)	C305—C306—H306	120.7
P2—C2—H2A	110.2	C308—C307—C312	120.0 (4)
P1—C2—H2A	110.2	C308—C307—P3	124.3 (3)
P2—C2—H2B	110.2	C312—C307—P3	115.7 (3)
P1—C2—H2B	110.2	C307—C308—C309	119.8 (5)
H2A—C2—H2B	108.5	C307—C308—H308	120.1
P3—C3—P4	108.5 (2)	C309—C308—H308	120.1
P3—C3—H3A	110.0	C310—C309—C308	120.4 (5)
P4—C3—H3A	110.0	C310—C309—H309	119.8
P3—C3—H3B	110.0	C308—C309—H309	119.8
P4—C3—H3B	110.0	C309—C310—C311	120.3 (5)
H3A—C3—H3B	108.4	C309—C310—H310	119.9
C102—C101—C106	118.6 (4)	C311—C310—H310	119.9
C102—C101—P1	117.4 (4)	C310—C311—C312	120.2 (5)
C106—C101—P1	123.9 (4)	C310—C311—H311	119.9
C101—C102—C103	120.3 (5)	C312—C311—H311	119.9
C101—C102—H102	119.8	C311—C312—C307	119.3 (5)
C103—C102—H102	119.8	C311—C312—H312	120.3
C104—C103—C102	120.4 (5)	C307—C312—H312	120.3
C104—C103—H103	119.8	C406—C401—C402	117.8 (4)
C102—C103—H103	119.8	C406—C401—P4	120.6 (3)
C103—C104—C105	119.9 (5)	C402—C401—P4	121.6 (4)
C103—C104—H104	120.0	C401—C402—C403	119.5 (6)
C105—C104—H104	120.0	C401—C402—H402	120.3
C104—C105—C106	121.1 (6)	C403—C402—H402	120.3
C104—C105—H105	119.5	C404—C403—C402	121.2 (6)
C106—C105—H105	119.5	C404—C403—H403	119.4
C105—C106—C101	119.6 (5)	C402—C403—H403	119.4
C105—C106—H106	120.2	C405—C404—C403	119.7 (5)
C101—C106—H106	120.2	C405—C404—H404	120.2
C112—C107—C108	119.4 (5)	C403—C404—H404	120.2
C112—C107—P1	121.6 (4)	C404—C405—C406	120.3 (5)
C108—C107—P1	119.0 (4)	C404—C405—H405	119.8
C107—C108—C109	119.3 (7)	C406—C405—H405	119.8
C107—C108—H108	120.3	C401—C406—C405	121.4 (4)
C109—C108—H108	120.3	C401—C406—H406	119.3
C110—C109—C108	120.2 (7)	C405—C406—H406	119.3
C110—C109—H109	119.9	C412—C407—C408	119.3 (4)
C108—C109—H109	119.9	C412—C407—P4	125.0 (4)
C109—C110—C111	121.1 (6)	C408—C407—P4	115.6 (3)
C109—C110—H110	119.4	C409—C408—C407	119.1 (5)
C111—C110—H110	119.4	C409—C408—H408	120.5
C110—C111—C112	119.5 (7)	C407—C408—H408	120.5
C110—C111—H111	120.2	C410—C409—C408	121.4 (5)
C112—C111—H111	120.2	C410—C409—H409	119.3
C107—C112—C111	120.5 (6)	C408—C409—H409	119.3
C107—C112—H112	119.8	C409—C410—C411	120.1 (5)
C111—C112—H112	119.8	C409—C410—H410	119.9
C202—C201—C206	120.3 (4)	C411—C410—H410	119.9
C202—C201—P2	118.8 (3)	C410—C411—C412	119.5 (5)
C206—C201—P2	120.9 (3)	C410—C411—H411	120.2
C201—C202—C203	119.5 (4)	C412—C411—H411	120.2
C201—C202—H202	120.3	C407—C412—C411	120.5 (5)
C203—C202—H202	120.3	C407—C412—H412	119.7
C204—C203—C202	119.8 (5)	C411—C412—H412	119.7
C204—C203—H203	120.1		
			
C301—P3—C1—P2	15.8 (3)	C1—P2—C207—C208	−19.9 (4)
C3—P3—C1—P2	−108.4 (2)	C2—P2—C207—C212	50.2 (4)
C307—P3—C1—P2	135.0 (2)	C201—P2—C207—C212	−64.4 (4)
C301—P3—C1—Ir1	146.8 (2)	C1—P2—C207—C212	162.4 (3)
C3—P3—C1—Ir1	22.6 (2)	C212—C207—C208—C209	−1.8 (7)
C307—P3—C1—Ir1	−94.0 (2)	P2—C207—C208—C209	−179.5 (4)
C207—P2—C1—P3	72.9 (3)	C207—C208—C209—C210	1.6 (7)
C2—P2—C1—P3	−169.4 (2)	C208—C209—C210—C211	−0.4 (8)
C201—P2—C1—P3	−55.5 (3)	C209—C210—C211—C212	−0.5 (9)
C207—P2—C1—Ir1	−60.9 (2)	C210—C211—C212—C207	0.3 (8)
C2—P2—C1—Ir1	56.8 (2)	C208—C207—C212—C211	0.9 (7)
C201—P2—C1—Ir1	170.75 (19)	P2—C207—C212—C211	178.7 (4)
C207—P2—C2—P1	70.3 (3)	C3—P3—C301—C306	19.4 (5)
C201—P2—C2—P1	−174.3 (2)	C307—P3—C301—C306	134.6 (4)
C1—P2—C2—P1	−52.0 (2)	C1—P3—C301—C306	−103.0 (4)
C101—P1—C2—P2	153.4 (2)	C3—P3—C301—C302	−161.6 (3)
C107—P1—C2—P2	−97.9 (2)	C307—P3—C301—C302	−46.4 (4)
Ir1—P1—C2—P2	27.2 (2)	C1—P3—C301—C302	76.0 (4)
C301—P3—C3—P4	−162.2 (2)	C306—C301—C302—C303	0.6 (7)
C307—P3—C3—P4	83.3 (2)	P3—C301—C302—C303	−178.4 (4)
C1—P3—C3—P4	−36.3 (2)	C301—C302—C303—C304	−1.2 (7)
C407—P4—C3—P3	−96.3 (2)	C302—C303—C304—C305	1.2 (8)
C401—P4—C3—P3	157.3 (2)	C303—C304—C305—C306	−0.5 (8)
Ir1—P4—C3—P3	34.5 (2)	C302—C301—C306—C305	0.0 (7)
C107—P1—C101—C102	65.4 (4)	P3—C301—C306—C305	179.0 (4)
C2—P1—C101—C102	174.9 (4)	C304—C305—C306—C301	−0.1 (8)
Ir1—P1—C101—C102	−67.4 (4)	C301—P3—C307—C308	132.2 (4)
C107—P1—C101—C106	−120.1 (4)	C3—P3—C307—C308	−109.4 (4)
C2—P1—C101—C106	−10.6 (4)	C1—P3—C307—C308	8.1 (4)
Ir1—P1—C101—C106	107.1 (4)	C301—P3—C307—C312	−49.3 (4)
C106—C101—C102—C103	2.0 (7)	C3—P3—C307—C312	69.0 (4)
P1—C101—C102—C103	176.8 (4)	C1—P3—C307—C312	−173.5 (4)
C101—C102—C103—C104	−1.2 (8)	C312—C307—C308—C309	0.3 (7)
C102—C103—C104—C105	−1.0 (8)	P3—C307—C308—C309	178.7 (4)
C103—C104—C105—C106	2.3 (8)	C307—C308—C309—C310	0.1 (8)
C104—C105—C106—C101	−1.4 (8)	C308—C309—C310—C311	−0.5 (9)
C102—C101—C106—C105	−0.8 (7)	C309—C310—C311—C312	0.5 (10)
P1—C101—C106—C105	−175.2 (4)	C310—C311—C312—C307	0.0 (9)
C101—P1—C107—C112	−128.5 (4)	C308—C307—C312—C311	−0.4 (8)
C2—P1—C107—C112	121.7 (4)	P3—C307—C312—C311	−178.9 (5)
Ir1—P1—C107—C112	4.8 (4)	C407—P4—C401—C406	−31.6 (4)
C101—P1—C107—C108	51.3 (4)	C3—P4—C401—C406	78.7 (4)
C2—P1—C107—C108	−58.5 (4)	Ir1—P4—C401—C406	−166.6 (3)
Ir1—P1—C107—C108	−175.4 (3)	C407—P4—C401—C402	149.2 (6)
C112—C107—C108—C109	1.2 (8)	C3—P4—C401—C402	−100.5 (6)
P1—C107—C108—C109	−178.7 (5)	Ir1—P4—C401—C402	14.2 (6)
C107—C108—C109—C110	−0.4 (10)	C406—C401—C402—C403	−3.8 (12)
C108—C109—C110—C111	0.0 (10)	P4—C401—C402—C403	175.4 (8)
C109—C110—C111—C112	−0.4 (10)	C401—C402—C403—C404	1.5 (17)
C108—C107—C112—C111	−1.6 (8)	C402—C403—C404—C405	1.8 (16)
P1—C107—C112—C111	178.3 (4)	C403—C404—C405—C406	−2.7 (12)
C110—C111—C112—C107	1.2 (9)	C402—C401—C406—C405	3.0 (9)
C207—P2—C201—C202	166.2 (4)	P4—C401—C406—C405	−176.2 (4)
C2—P2—C201—C202	49.3 (4)	C404—C405—C406—C401	0.3 (9)
C1—P2—C201—C202	−61.2 (4)	C401—P4—C407—C412	98.8 (4)
C207—P2—C201—C206	−11.8 (4)	C3—P4—C407—C412	−6.0 (4)
C2—P2—C201—C206	−128.8 (4)	Ir1—P4—C407—C412	−128.2 (4)
C1—P2—C201—C206	120.7 (4)	C401—P4—C407—C408	−76.8 (4)
C206—C201—C202—C203	1.9 (8)	C3—P4—C407—C408	178.5 (3)
P2—C201—C202—C203	−176.2 (4)	Ir1—P4—C407—C408	56.3 (4)
C201—C202—C203—C204	−0.1 (9)	C412—C407—C408—C409	−2.6 (7)
C202—C203—C204—C205	−2.2 (9)	P4—C407—C408—C409	173.1 (4)
C203—C204—C205—C206	2.8 (8)	C407—C408—C409—C410	2.1 (8)
C204—C205—C206—C201	−1.0 (7)	C408—C409—C410—C411	−0.4 (9)
C202—C201—C206—C205	−1.3 (7)	C409—C410—C411—C412	−0.7 (9)
P2—C201—C206—C205	176.7 (4)	C408—C407—C412—C411	1.6 (7)
C2—P2—C207—C208	−132.1 (4)	P4—C407—C412—C411	−173.7 (4)
C201—P2—C207—C208	113.3 (4)	C410—C411—C412—C407	0.1 (8)

### Dichloridohydrido(1,1,3,3,5,5,7,7-octaphenyl-1,7-diphospha-3,5-diphosphoniaheptan-4-yl)iridium(III) chloride pentahydrate (3) Hydrogen-bond geometry (Å, º)

**Table:**

*D*—H···*A*	*D*—H	H···*A*	*D*···*A*	*D*—H···*A*
C1—H1*A*···Cl3	0.99 (4)	2.48 (4)	3.422 (6)	159 (3)
C2—H2*B*···Cl3	0.98	2.63	3.450 (6)	142
C202—H202···Cl3	0.94	2.67	3.584 (6)	166
C308—H308···Cl3	0.94	2.70	3.475 (7)	141
C3—H3*B*···Cl3*A*	0.98	2.58	3.489 (12)	155

### Carbonylchloridohydrido(1,1,3,3,5,5,7,7-octaphenyl-1,5λ5,7-triphospha-3-phosphoniahept-4-en-4-yl)iridium(III) chloride–methanol–water (1/2/1) (4) Crystal data

**Table:**

[Ir(C_51_H_44_P_4_)ClH(CO)]Cl·2CH_4_O·H_2_O	*F*(000) = 2328
*M**_r_* = 1154.96	*D*_x_ = 1.390 Mg m^−^^3^
Monoclinic, *P*2_1_/*c*	Mo *K*α radiation, λ = 0.71073 Å
*a* = 12.5929 (2) Å	Cell parameters from 183501 reflections
*b* = 23.2803 (4) Å	θ = 1.0–25.0°
*c* = 19.7488 (4) Å	µ = 2.67 mm^−^^1^
β = 107.535 (1)°	*T* = 233 K
*V* = 5520.66 (17) Å^3^	Prism, colourless
*Z* = 4	0.21 × 0.10 × 0.07 mm

### Carbonylchloridohydrido(1,1,3,3,5,5,7,7-octaphenyl-1,5λ5,7-triphospha-3-phosphoniahept-4-en-4-yl)iridium(III) chloride–methanol–water (1/2/1) (4) Data collection

**Table:**

Nonius KappaCCD diffractometer	*R*_int_ = 0.049
Detector resolution: 9.4 pixels mm^-1^	θ_max_ = 25.0°, θ_min_ = 1.4°
phi– and ω–scans	*h* = −14→14
32186 measured reflections	*k* = −27→27
9675 independent reflections	*l* = −23→21
8070 reflections with *I* > 2σ(*I*)	

### Carbonylchloridohydrido(1,1,3,3,5,5,7,7-octaphenyl-1,5λ5,7-triphospha-3-phosphoniahept-4-en-4-yl)iridium(III) chloride–methanol–water (1/2/1) (4) Refinement

**Table:**

Refinement on *F*^2^	Primary atom site location: structure-invariant direct methods
Least-squares matrix: full	Secondary atom site location: difference Fourier map
*R*[*F*^2^ > 2σ(*F*^2^)] = 0.041	Hydrogen site location: mixed
*wR*(*F*^2^) = 0.095	H atoms treated by a mixture of independent and constrained refinement
*S* = 1.13	*w* = 1/[σ^2^(*F*_o_^2^) + (0.0255*P*)^2^ + 16.7009*P*] where *P* = (*F*_o_^2^ + 2*F*_c_^2^)/3
9675 reflections	(Δ/σ)_max_ = 0.001
624 parameters	Δρ_max_ = 1.85 e Å^−^^3^
1 restraint	Δρ_min_ = −1.45 e Å^−^^3^

### Carbonylchloridohydrido(1,1,3,3,5,5,7,7-octaphenyl-1,5λ5,7-triphospha-3-phosphoniahept-4-en-4-yl)iridium(III) chloride–methanol–water (1/2/1) (4) Special details

**Table:**

Geometry. All esds (except the esd in the dihedral angle between two l.s. planes) are estimated using the full covariance matrix. The cell esds are taken into account individually in the estimation of esds in distances, angles and torsion angles; correlations between esds in cell parameters are only used when they are defined by crystal symmetry. An approximate (isotropic) treatment of cell esds is used for estimating esds involving l.s. planes.
Refinement. Hydrogen H1 at Ir1 was found and refined with bond restraint (*d* = 1.6 Å). Hydrogen atoms at solvent MeOH and H2O were omitted. One Cl-anion is positionally disordered in ratio 1:1 for Cl2 and Cl2A. Maybe because of this disorder two MeOH positions C6-O3 and C7-O4 are only half occupied, also a water molecule, which is split in four positions with occupancy of 0.25 for each position. O5, O5A, O5B and O5C were refined with isotropic displacement par..

### Carbonylchloridohydrido(1,1,3,3,5,5,7,7-octaphenyl-1,5λ5,7-triphospha-3-phosphoniahept-4-en-4-yl)iridium(III) chloride–methanol–water (1/2/1) (4) Fractional atomic coordinates and isotropic or equivalent isotropic displacement parameters (Å^2^)

**Table:**

	*x*	*y*	*z*	*U*_iso_*/*U*_eq_	Occ. (<1)
H1	0.644 (4)	0.060 (2)	0.7395 (17)	0.050 (15)*	
Ir1	0.64126 (2)	0.08694 (2)	0.80985 (2)	0.03393 (8)	
P1	0.83264 (11)	0.09659 (6)	0.83213 (7)	0.0343 (3)	
P2	0.73619 (12)	0.20938 (6)	0.77950 (7)	0.0347 (3)	
P3	0.50434 (11)	0.17312 (6)	0.68744 (7)	0.0332 (3)	
P4	0.44822 (11)	0.08043 (6)	0.77016 (7)	0.0330 (3)	
Cl1	0.66243 (13)	−0.01003 (6)	0.85840 (8)	0.0504 (4)	
O1	0.6356 (4)	0.1273 (2)	0.9575 (2)	0.0665 (13)	
C1	0.6243 (4)	0.1661 (2)	0.7541 (3)	0.0339 (12)	
C2	0.8568 (4)	0.1636 (2)	0.7904 (3)	0.0374 (12)	
H2A	0.9239	0.1827	0.8204	0.045*	
H2B	0.8673	0.1556	0.7441	0.045*	
C3	0.3970 (4)	0.1475 (2)	0.7242 (3)	0.0374 (12)	
H3A	0.3272	0.1409	0.6863	0.045*	
H3B	0.3833	0.1757	0.7574	0.045*	
C4	0.6406 (5)	0.1167 (2)	0.9034 (3)	0.0446 (14)	
C101	0.9032 (4)	0.0419 (2)	0.7962 (3)	0.0395 (13)	
C102	0.8500 (6)	−0.0057 (3)	0.7608 (4)	0.0617 (18)	
H102	0.7732	−0.0102	0.7535	0.074*	
C103	0.9064 (6)	−0.0467 (3)	0.7359 (4)	0.079 (2)	
H103	0.8683	−0.0792	0.7124	0.094*	
C104	1.0176 (7)	−0.0407 (3)	0.7449 (4)	0.073 (2)	
H104	1.0553	−0.0687	0.7266	0.087*	
C105	1.0733 (6)	0.0048 (4)	0.7796 (4)	0.069 (2)	
H105	1.1503	0.0081	0.7868	0.083*	
C106	1.0170 (5)	0.0475 (3)	0.8051 (3)	0.0537 (16)	
H106	1.0558	0.0800	0.8281	0.064*	
C107	0.9162 (5)	0.1007 (2)	0.9252 (3)	0.0419 (13)	
C108	0.8914 (6)	0.0631 (3)	0.9724 (3)	0.0579 (17)	
H108	0.8307	0.0378	0.9566	0.069*	
C109	0.9569 (7)	0.0625 (4)	1.0440 (4)	0.073 (2)	
H109	0.9402	0.0366	1.0758	0.088*	
C110	1.0440 (6)	0.0993 (4)	1.0675 (4)	0.073 (2)	
H110	1.0864	0.0995	1.1156	0.087*	
C111	1.0698 (6)	0.1362 (4)	1.0202 (4)	0.076 (2)	
H111	1.1317	0.1607	1.0360	0.091*	
C112	1.0056 (5)	0.1375 (3)	0.9499 (3)	0.0570 (17)	
H112	1.0229	0.1637	0.9186	0.068*	
C201	0.7471 (4)	0.2682 (2)	0.7220 (3)	0.0384 (13)	
C202	0.7142 (5)	0.3229 (2)	0.7355 (3)	0.0507 (15)	
H202	0.6848	0.3288	0.7734	0.061*	
C203	0.7248 (6)	0.3685 (3)	0.6932 (4)	0.0620 (18)	
H203	0.7033	0.4055	0.7030	0.074*	
C204	0.7662 (6)	0.3605 (3)	0.6375 (4)	0.0643 (19)	
H204	0.7720	0.3916	0.6085	0.077*	
C205	0.7992 (6)	0.3064 (3)	0.6242 (4)	0.067 (2)	
H205	0.8286	0.3007	0.5862	0.081*	
C206	0.7897 (6)	0.2606 (3)	0.6658 (3)	0.0525 (16)	
H206	0.8122	0.2238	0.6560	0.063*	
C207	0.7507 (5)	0.2427 (2)	0.8647 (3)	0.0440 (14)	
C208	0.6563 (6)	0.2515 (3)	0.8843 (3)	0.0571 (17)	
H208	0.5875	0.2377	0.8552	0.069*	
C209	0.6609 (8)	0.2808 (3)	0.9469 (4)	0.075 (2)	
H209	0.5959	0.2866	0.9601	0.090*	
C210	0.7607 (9)	0.3006 (3)	0.9885 (4)	0.082 (3)	
H210	0.7642	0.3208	1.0304	0.099*	
C211	0.8553 (8)	0.2918 (3)	0.9707 (4)	0.085 (3)	
H211	0.9239	0.3046	1.0011	0.102*	
C212	0.8513 (6)	0.2636 (3)	0.9068 (4)	0.0638 (19)	
H212	0.9162	0.2590	0.8932	0.077*	
C301	0.4691 (4)	0.2451 (2)	0.6542 (3)	0.0391 (13)	
C302	0.4270 (5)	0.2844 (2)	0.6909 (3)	0.0516 (15)	
H302	0.4126	0.2734	0.7331	0.062*	
C303	0.4053 (6)	0.3402 (3)	0.6663 (4)	0.0657 (19)	
H303	0.3771	0.3671	0.6919	0.079*	
C304	0.4257 (6)	0.3561 (3)	0.6037 (4)	0.067 (2)	
H304	0.4112	0.3939	0.5870	0.080*	
C305	0.4661 (7)	0.3176 (3)	0.5667 (4)	0.071 (2)	
H305	0.4797	0.3288	0.5243	0.085*	
C306	0.4878 (5)	0.2615 (3)	0.5911 (3)	0.0536 (16)	
H306	0.5150	0.2347	0.5648	0.064*	
C307	0.4905 (5)	0.1297 (2)	0.6088 (3)	0.0433 (14)	
C308	0.3859 (6)	0.1179 (3)	0.5619 (3)	0.0643 (19)	
H308	0.3210	0.1304	0.5716	0.077*	
C309	0.3795 (9)	0.0871 (4)	0.5001 (4)	0.098 (3)	
H309	0.3092	0.0778	0.4687	0.117*	
C310	0.4718 (10)	0.0702 (4)	0.4845 (5)	0.097 (3)	
H310	0.4654	0.0507	0.4418	0.117*	
C311	0.5755 (9)	0.0815 (3)	0.5311 (5)	0.084 (3)	
H311	0.6398	0.0693	0.5204	0.101*	
C312	0.5854 (6)	0.1112 (3)	0.5943 (3)	0.0552 (16)	
H312	0.6560	0.1185	0.6266	0.066*	
C401	0.3817 (4)	0.0722 (2)	0.8399 (3)	0.0374 (13)	
C402	0.3996 (5)	0.0217 (3)	0.8779 (3)	0.0559 (17)	
H402	0.4423	−0.0077	0.8665	0.067*	
C403	0.3541 (6)	0.0146 (3)	0.9331 (4)	0.0645 (19)	
H403	0.3669	−0.0198	0.9592	0.077*	
C404	0.2915 (6)	0.0563 (3)	0.9503 (3)	0.0617 (19)	
H404	0.2604	0.0508	0.9875	0.074*	
C405	0.2744 (6)	0.1063 (3)	0.9129 (4)	0.0613 (18)	
H405	0.2323	0.1355	0.9252	0.074*	
C406	0.3175 (5)	0.1148 (3)	0.8575 (3)	0.0474 (14)	
H406	0.3036	0.1494	0.8316	0.057*	
C407	0.3831 (5)	0.0233 (2)	0.7083 (3)	0.0391 (13)	
C408	0.2687 (5)	0.0258 (3)	0.6742 (3)	0.0552 (16)	
H408	0.2268	0.0574	0.6815	0.066*	
C409	0.2176 (6)	−0.0183 (3)	0.6299 (4)	0.075 (2)	
H409	0.1407	−0.0165	0.6064	0.090*	
C410	0.2773 (8)	−0.0644 (3)	0.6196 (4)	0.074 (2)	
H410	0.2417	−0.0942	0.5890	0.089*	
C411	0.3882 (7)	−0.0673 (3)	0.6536 (4)	0.068 (2)	
H411	0.4287	−0.0997	0.6472	0.082*	
C412	0.4429 (5)	−0.0233 (2)	0.6973 (3)	0.0500 (15)	
H412	0.5202	−0.0252	0.7193	0.060*	
Cl2	1.1164 (3)	0.2433 (2)	0.8501 (3)	0.1024 (15)	0.5
Cl2A	0.1116 (4)	0.1659 (3)	0.6239 (3)	0.138 (2)	0.5
O2	0.8990 (6)	0.1235 (3)	0.6346 (3)	0.122 (2)	
C5	0.9032 (14)	0.0693 (6)	0.6038 (7)	0.180 (7)	
O3	0.3556 (9)	0.2629 (5)	0.8426 (7)	0.087 (3)	0.5
C6	0.3639 (17)	0.3141 (13)	0.8598 (15)	0.152 (11)	0.5
O4	0.1179 (11)	0.1628 (8)	0.4713 (7)	0.131 (5)	0.5
C7	0.0628 (18)	0.1103 (11)	0.4395 (13)	0.133 (9)	0.5
O5	0.1070 (15)	0.1876 (9)	0.6998 (10)	0.068 (5)*	0.25
O5A	0.1762 (16)	0.2452 (9)	0.7503 (10)	0.072 (5)*	0.25
O5C	0.136 (2)	0.2978 (13)	0.7172 (16)	0.127 (9)*	0.25
O5D	0.112 (3)	0.2330 (19)	0.672 (2)	0.175 (14)*	0.25

### Carbonylchloridohydrido(1,1,3,3,5,5,7,7-octaphenyl-1,5λ5,7-triphospha-3-phosphoniahept-4-en-4-yl)iridium(III) chloride–methanol–water (1/2/1) (4) Atomic displacement parameters (Å^2^)

**Table:**

	*U*^11^	*U*^22^	*U*^33^	*U*^12^	*U*^13^	*U*^23^
Ir1	0.03495 (12)	0.02924 (12)	0.03939 (13)	0.00201 (9)	0.01392 (9)	0.00953 (9)
P1	0.0336 (7)	0.0349 (8)	0.0358 (7)	0.0023 (6)	0.0126 (6)	0.0050 (6)
P2	0.0411 (8)	0.0297 (7)	0.0338 (7)	−0.0030 (6)	0.0120 (6)	0.0032 (6)
P3	0.0385 (7)	0.0265 (7)	0.0358 (8)	0.0028 (6)	0.0130 (6)	0.0047 (6)
P4	0.0351 (7)	0.0275 (7)	0.0387 (8)	0.0003 (6)	0.0145 (6)	0.0041 (6)
Cl1	0.0576 (9)	0.0330 (7)	0.0618 (9)	0.0037 (6)	0.0201 (7)	0.0177 (7)
O1	0.086 (3)	0.079 (3)	0.041 (3)	0.000 (3)	0.029 (2)	−0.004 (2)
C1	0.036 (3)	0.028 (3)	0.038 (3)	0.000 (2)	0.012 (2)	0.008 (2)
C2	0.037 (3)	0.037 (3)	0.038 (3)	−0.005 (2)	0.013 (2)	0.004 (2)
C3	0.036 (3)	0.037 (3)	0.040 (3)	0.004 (2)	0.014 (2)	0.008 (2)
C4	0.045 (3)	0.036 (3)	0.053 (4)	−0.001 (3)	0.014 (3)	0.011 (3)
C101	0.041 (3)	0.042 (3)	0.036 (3)	0.005 (3)	0.013 (2)	0.009 (3)
C102	0.059 (4)	0.055 (4)	0.081 (5)	−0.004 (3)	0.036 (4)	−0.015 (4)
C103	0.071 (5)	0.068 (5)	0.103 (6)	0.003 (4)	0.035 (5)	−0.030 (4)
C104	0.075 (5)	0.076 (5)	0.072 (5)	0.026 (4)	0.031 (4)	−0.009 (4)
C105	0.050 (4)	0.099 (6)	0.059 (4)	0.020 (4)	0.019 (3)	−0.006 (4)
C106	0.046 (4)	0.067 (4)	0.048 (4)	0.006 (3)	0.015 (3)	−0.004 (3)
C107	0.043 (3)	0.047 (3)	0.037 (3)	0.010 (3)	0.013 (3)	0.003 (3)
C108	0.061 (4)	0.069 (4)	0.042 (4)	0.004 (3)	0.013 (3)	0.016 (3)
C109	0.088 (6)	0.089 (6)	0.046 (4)	0.020 (5)	0.026 (4)	0.023 (4)
C110	0.071 (5)	0.102 (6)	0.040 (4)	0.013 (5)	0.008 (4)	0.000 (4)
C111	0.066 (5)	0.095 (6)	0.055 (5)	−0.002 (4)	−0.001 (4)	−0.012 (4)
C112	0.055 (4)	0.066 (4)	0.047 (4)	−0.003 (3)	0.010 (3)	0.004 (3)
C201	0.046 (3)	0.030 (3)	0.039 (3)	−0.011 (2)	0.012 (2)	0.002 (2)
C202	0.065 (4)	0.038 (3)	0.050 (4)	−0.007 (3)	0.018 (3)	0.003 (3)
C203	0.088 (5)	0.026 (3)	0.071 (5)	−0.009 (3)	0.023 (4)	0.005 (3)
C204	0.090 (5)	0.044 (4)	0.061 (4)	−0.020 (4)	0.025 (4)	0.016 (3)
C205	0.099 (6)	0.059 (5)	0.052 (4)	−0.010 (4)	0.036 (4)	0.014 (3)
C206	0.081 (5)	0.035 (3)	0.049 (4)	−0.007 (3)	0.031 (3)	0.003 (3)
C207	0.059 (4)	0.035 (3)	0.036 (3)	0.005 (3)	0.011 (3)	0.002 (2)
C208	0.081 (5)	0.049 (4)	0.047 (4)	0.001 (3)	0.028 (3)	0.001 (3)
C209	0.128 (7)	0.058 (5)	0.057 (5)	0.002 (5)	0.054 (5)	−0.004 (4)
C210	0.143 (9)	0.053 (5)	0.049 (5)	0.022 (5)	0.026 (5)	0.001 (4)
C211	0.110 (7)	0.059 (5)	0.057 (5)	0.012 (5)	−0.018 (5)	−0.014 (4)
C212	0.069 (5)	0.050 (4)	0.059 (4)	0.001 (3)	−0.001 (3)	−0.007 (3)
C301	0.042 (3)	0.028 (3)	0.046 (3)	0.005 (2)	0.011 (3)	0.007 (2)
C302	0.069 (4)	0.038 (3)	0.052 (4)	0.009 (3)	0.025 (3)	0.004 (3)
C303	0.081 (5)	0.039 (4)	0.084 (5)	0.015 (3)	0.034 (4)	0.004 (3)
C304	0.095 (5)	0.032 (3)	0.074 (5)	0.014 (3)	0.027 (4)	0.018 (3)
C305	0.111 (6)	0.048 (4)	0.062 (5)	0.016 (4)	0.040 (4)	0.025 (3)
C306	0.078 (4)	0.038 (3)	0.052 (4)	0.013 (3)	0.032 (3)	0.015 (3)
C307	0.066 (4)	0.027 (3)	0.039 (3)	−0.003 (3)	0.019 (3)	0.001 (2)
C308	0.069 (5)	0.077 (5)	0.045 (4)	−0.010 (4)	0.015 (3)	−0.009 (3)
C309	0.132 (8)	0.107 (7)	0.051 (5)	−0.053 (6)	0.023 (5)	−0.022 (5)
C310	0.153 (10)	0.090 (6)	0.062 (5)	−0.036 (6)	0.053 (6)	−0.029 (5)
C311	0.131 (8)	0.065 (5)	0.079 (6)	−0.002 (5)	0.068 (6)	−0.013 (4)
C312	0.080 (5)	0.043 (3)	0.055 (4)	−0.001 (3)	0.038 (4)	−0.004 (3)
C401	0.034 (3)	0.039 (3)	0.040 (3)	−0.009 (2)	0.013 (2)	0.002 (2)
C402	0.051 (4)	0.062 (4)	0.062 (4)	0.000 (3)	0.028 (3)	0.019 (3)
C403	0.063 (4)	0.071 (5)	0.060 (4)	−0.009 (4)	0.020 (4)	0.025 (4)
C404	0.061 (4)	0.086 (5)	0.043 (4)	−0.025 (4)	0.022 (3)	−0.002 (4)
C405	0.059 (4)	0.078 (5)	0.057 (4)	−0.011 (4)	0.034 (3)	−0.013 (4)
C406	0.054 (4)	0.045 (3)	0.051 (4)	−0.010 (3)	0.027 (3)	−0.007 (3)
C407	0.053 (4)	0.029 (3)	0.039 (3)	−0.006 (2)	0.021 (3)	−0.001 (2)
C408	0.047 (4)	0.048 (4)	0.068 (4)	−0.005 (3)	0.014 (3)	−0.010 (3)
C409	0.061 (5)	0.087 (6)	0.068 (5)	−0.030 (4)	0.007 (4)	−0.017 (4)
C410	0.105 (7)	0.058 (5)	0.064 (5)	−0.023 (5)	0.032 (5)	−0.030 (4)
C411	0.098 (6)	0.049 (4)	0.066 (5)	−0.007 (4)	0.036 (4)	−0.014 (3)
C412	0.061 (4)	0.042 (4)	0.054 (4)	−0.006 (3)	0.028 (3)	−0.004 (3)
Cl2	0.064 (2)	0.095 (3)	0.151 (4)	−0.021 (2)	0.036 (3)	0.008 (3)
Cl2A	0.078 (3)	0.158 (5)	0.147 (5)	0.031 (3)	−0.014 (3)	0.005 (4)
O2	0.148 (6)	0.140 (6)	0.089 (5)	0.062 (5)	0.050 (4)	0.033 (4)
C5	0.250 (17)	0.158 (12)	0.113 (10)	0.110 (12)	0.028 (10)	0.008 (9)
O3	0.092 (8)	0.066 (7)	0.126 (10)	−0.019 (6)	0.068 (7)	−0.019 (7)
C6	0.077 (13)	0.20 (3)	0.20 (3)	−0.054 (16)	0.077 (15)	−0.10 (2)
O4	0.093 (9)	0.193 (16)	0.101 (10)	0.006 (10)	0.021 (8)	−0.004 (10)
C7	0.103 (15)	0.15 (2)	0.139 (19)	−0.001 (14)	0.028 (14)	−0.081 (17)

### Carbonylchloridohydrido(1,1,3,3,5,5,7,7-octaphenyl-1,5λ5,7-triphospha-3-phosphoniahept-4-en-4-yl)iridium(III) chloride–methanol–water (1/2/1) (4) Geometric parameters (Å, º)

**Table:**

Ir1—C4	1.975 (7)	C207—C212	1.377 (8)
Ir1—C1	2.124 (5)	C208—C209	1.397 (9)
Ir1—P4	2.3235 (13)	C208—H208	0.9400
Ir1—P1	2.3265 (14)	C209—C210	1.359 (11)
Ir1—Cl1	2.4359 (13)	C209—H209	0.9400
Ir1—H1	1.535 (19)	C210—C211	1.356 (12)
P1—C101	1.815 (6)	C210—H210	0.9400
P1—C107	1.824 (6)	C211—C212	1.411 (10)
P1—C2	1.833 (5)	C211—H211	0.9400
P2—C1	1.681 (5)	C212—H212	0.9400
P2—C201	1.809 (5)	C301—C302	1.368 (8)
P2—C207	1.812 (6)	C301—C306	1.391 (8)
P2—C2	1.814 (5)	C302—C303	1.385 (8)
P3—C1	1.686 (5)	C302—H302	0.9400
P3—C301	1.806 (5)	C303—C304	1.387 (10)
P3—C307	1.817 (6)	C303—H303	0.9400
P3—C3	1.818 (5)	C304—C305	1.348 (9)
P4—C3	1.822 (5)	C304—H304	0.9400
P4—C401	1.825 (5)	C305—C306	1.391 (8)
P4—C407	1.825 (5)	C305—H305	0.9400
O1—C4	1.117 (7)	C306—H306	0.9400
C2—H2A	0.9800	C307—C312	1.379 (8)
C2—H2B	0.9800	C307—C308	1.390 (8)
C3—H3A	0.9800	C308—C309	1.398 (10)
C3—H3B	0.9800	C308—H308	0.9400
C101—C102	1.373 (8)	C309—C310	1.347 (13)
C101—C106	1.397 (8)	C309—H309	0.9400
C102—C103	1.365 (9)	C310—C311	1.378 (12)
C102—H102	0.9400	C310—H310	0.9400
C103—C104	1.365 (10)	C311—C312	1.398 (9)
C103—H103	0.9400	C311—H311	0.9400
C104—C105	1.340 (10)	C312—H312	0.9400
C104—H104	0.9400	C401—C402	1.376 (8)
C105—C106	1.400 (9)	C401—C406	1.389 (8)
C105—H105	0.9400	C402—C403	1.386 (9)
C106—H106	0.9400	C402—H402	0.9400
C107—C108	1.382 (8)	C403—C404	1.357 (10)
C107—C112	1.383 (8)	C403—H403	0.9400
C108—C109	1.406 (9)	C404—C405	1.359 (10)
C108—H108	0.9400	C404—H404	0.9400
C109—C110	1.358 (11)	C405—C406	1.375 (8)
C109—H109	0.9400	C405—H405	0.9400
C110—C111	1.377 (11)	C406—H406	0.9400
C110—H110	0.9400	C407—C412	1.373 (8)
C111—C112	1.381 (9)	C407—C408	1.396 (8)
C111—H111	0.9400	C408—C409	1.377 (9)
C112—H112	0.9400	C408—H408	0.9400
C201—C206	1.383 (8)	C409—C410	1.361 (10)
C201—C202	1.389 (8)	C409—H409	0.9400
C202—C203	1.383 (8)	C410—C411	1.357 (11)
C202—H202	0.9400	C410—H410	0.9400
C203—C204	1.365 (9)	C411—C412	1.382 (9)
C203—H203	0.9400	C411—H411	0.9400
C204—C205	1.377 (10)	C412—H412	0.9400
C204—H204	0.9400	Cl2A—O5	1.60 (2)
C205—C206	1.373 (8)	O2—C5	1.409 (14)
C205—H205	0.9400	O3—C6	1.24 (3)
C206—H206	0.9400	O4—C7	1.45 (2)
C207—C208	1.372 (9)		
			
C4—Ir1—C1	98.8 (2)	C203—C204—C205	119.3 (6)
C4—Ir1—P4	92.27 (17)	C203—C204—H204	120.3
C1—Ir1—P4	87.15 (14)	C205—C204—H204	120.3
C4—Ir1—P1	94.89 (17)	C206—C205—C204	120.7 (6)
C1—Ir1—P1	87.18 (14)	C206—C205—H205	119.7
P4—Ir1—P1	171.47 (5)	C204—C205—H205	119.7
C4—Ir1—Cl1	89.30 (16)	C205—C206—C201	120.4 (6)
C1—Ir1—Cl1	171.91 (15)	C205—C206—H206	119.8
P4—Ir1—Cl1	92.96 (5)	C201—C206—H206	119.8
P1—Ir1—Cl1	91.76 (5)	C208—C207—C212	119.7 (6)
C4—Ir1—H1	176 (2)	C208—C207—P2	118.2 (5)
C1—Ir1—H1	85 (2)	C212—C207—P2	121.9 (5)
P4—Ir1—H1	88.6 (19)	C207—C208—C209	120.9 (7)
P1—Ir1—H1	84.5 (19)	C207—C208—H208	119.5
Cl1—Ir1—H1	87 (2)	C209—C208—H208	119.5
C101—P1—C107	103.2 (2)	C210—C209—C208	119.0 (8)
C101—P1—C2	104.8 (2)	C210—C209—H209	120.5
C107—P1—C2	106.5 (3)	C208—C209—H209	120.5
C101—P1—Ir1	117.35 (19)	C211—C210—C209	121.1 (8)
C107—P1—Ir1	116.47 (19)	C211—C210—H210	119.5
C2—P1—Ir1	107.45 (17)	C209—C210—H210	119.5
C1—P2—C201	118.8 (2)	C210—C211—C212	120.4 (8)
C1—P2—C207	112.0 (3)	C210—C211—H211	119.8
C201—P2—C207	104.7 (3)	C212—C211—H211	119.8
C1—P2—C2	106.3 (2)	C207—C212—C211	118.9 (8)
C201—P2—C2	107.5 (3)	C207—C212—H212	120.6
C207—P2—C2	107.1 (3)	C211—C212—H212	120.6
C1—P3—C301	116.0 (3)	C302—C301—C306	119.3 (5)
C1—P3—C307	116.5 (3)	C302—C301—P3	121.3 (4)
C301—P3—C307	104.8 (3)	C306—C301—P3	119.4 (4)
C1—P3—C3	104.8 (2)	C301—C302—C303	120.5 (6)
C301—P3—C3	108.6 (2)	C301—C302—H302	119.7
C307—P3—C3	105.5 (3)	C303—C302—H302	119.7
C3—P4—C401	106.9 (2)	C302—C303—C304	119.5 (6)
C3—P4—C407	105.7 (3)	C302—C303—H303	120.2
C401—P4—C407	102.2 (2)	C304—C303—H303	120.2
C3—P4—Ir1	106.83 (17)	C305—C304—C303	120.5 (6)
C401—P4—Ir1	115.08 (17)	C305—C304—H304	119.7
C407—P4—Ir1	119.14 (19)	C303—C304—H304	119.7
P2—C1—P3	130.4 (3)	C304—C305—C306	120.2 (6)
P2—C1—Ir1	114.9 (3)	C304—C305—H305	119.9
P3—C1—Ir1	114.5 (3)	C306—C305—H305	119.9
P2—C2—P1	107.9 (3)	C301—C306—C305	120.0 (6)
P2—C2—H2A	110.1	C301—C306—H306	120.0
P1—C2—H2A	110.1	C305—C306—H306	120.0
P2—C2—H2B	110.1	C312—C307—C308	120.5 (6)
P1—C2—H2B	110.1	C312—C307—P3	119.0 (5)
H2A—C2—H2B	108.4	C308—C307—P3	120.4 (5)
P3—C3—P4	106.6 (3)	C307—C308—C309	118.5 (8)
P3—C3—H3A	110.4	C307—C308—H308	120.8
P4—C3—H3A	110.4	C309—C308—H308	120.8
P3—C3—H3B	110.4	C310—C309—C308	121.5 (9)
P4—C3—H3B	110.4	C310—C309—H309	119.2
H3A—C3—H3B	108.6	C308—C309—H309	119.2
O1—C4—Ir1	171.7 (5)	C309—C310—C311	120.1 (8)
C102—C101—C106	117.7 (6)	C309—C310—H310	120.0
C102—C101—P1	123.0 (4)	C311—C310—H310	120.0
C106—C101—P1	119.3 (5)	C310—C311—C312	120.1 (8)
C103—C102—C101	121.3 (6)	C310—C311—H311	119.9
C103—C102—H102	119.3	C312—C311—H311	119.9
C101—C102—H102	119.3	C307—C312—C311	119.3 (7)
C104—C103—C102	120.5 (7)	C307—C312—H312	120.4
C104—C103—H103	119.8	C311—C312—H312	120.4
C102—C103—H103	119.8	C402—C401—C406	119.1 (5)
C105—C104—C103	120.4 (7)	C402—C401—P4	117.7 (4)
C105—C104—H104	119.8	C406—C401—P4	123.2 (4)
C103—C104—H104	119.8	C401—C402—C403	119.6 (6)
C104—C105—C106	120.1 (7)	C401—C402—H402	120.2
C104—C105—H105	119.9	C403—C402—H402	120.2
C106—C105—H105	119.9	C404—C403—C402	121.3 (6)
C101—C106—C105	120.0 (6)	C404—C403—H403	119.3
C101—C106—H106	120.0	C402—C403—H403	119.3
C105—C106—H106	120.0	C403—C404—C405	119.0 (6)
C108—C107—C112	118.7 (6)	C403—C404—H404	120.5
C108—C107—P1	117.9 (5)	C405—C404—H404	120.5
C112—C107—P1	123.4 (5)	C404—C405—C406	121.4 (7)
C107—C108—C109	120.1 (7)	C404—C405—H405	119.3
C107—C108—H108	119.9	C406—C405—H405	119.3
C109—C108—H108	119.9	C405—C406—C401	119.6 (6)
C110—C109—C108	120.4 (7)	C405—C406—H406	120.2
C110—C109—H109	119.8	C401—C406—H406	120.2
C108—C109—H109	119.8	C412—C407—C408	119.5 (5)
C109—C110—C111	119.5 (7)	C412—C407—P4	121.6 (4)
C109—C110—H110	120.2	C408—C407—P4	118.8 (4)
C111—C110—H110	120.2	C409—C408—C407	119.4 (6)
C110—C111—C112	120.6 (7)	C409—C408—H408	120.3
C110—C111—H111	119.7	C407—C408—H408	120.3
C112—C111—H111	119.7	C410—C409—C408	120.7 (7)
C111—C112—C107	120.6 (7)	C410—C409—H409	119.6
C111—C112—H112	119.7	C408—C409—H409	119.6
C107—C112—H112	119.7	C411—C410—C409	119.8 (7)
C206—C201—C202	118.8 (5)	C411—C410—H410	120.1
C206—C201—P2	121.9 (4)	C409—C410—H410	120.1
C202—C201—P2	119.3 (4)	C410—C411—C412	121.1 (7)
C203—C202—C201	120.0 (6)	C410—C411—H411	119.4
C203—C202—H202	120.0	C412—C411—H411	119.4
C201—C202—H202	120.0	C407—C412—C411	119.4 (6)
C204—C203—C202	120.8 (6)	C407—C412—H412	120.3
C204—C203—H203	119.6	C411—C412—H412	120.3
C202—C203—H203	119.6		
			
C201—P2—C1—P3	−11.0 (5)	C2—P2—C207—C208	−143.9 (5)
C207—P2—C1—P3	111.3 (4)	C1—P2—C207—C212	157.9 (5)
C2—P2—C1—P3	−132.1 (4)	C201—P2—C207—C212	−72.2 (5)
C201—P2—C1—Ir1	163.9 (3)	C2—P2—C207—C212	41.7 (6)
C207—P2—C1—Ir1	−73.8 (3)	C212—C207—C208—C209	−0.8 (9)
C2—P2—C1—Ir1	42.9 (3)	P2—C207—C208—C209	−175.3 (5)
C301—P3—C1—P2	−20.9 (5)	C207—C208—C209—C210	0.1 (10)
C307—P3—C1—P2	103.2 (4)	C208—C209—C210—C211	−0.9 (11)
C3—P3—C1—P2	−140.6 (4)	C209—C210—C211—C212	2.4 (12)
C301—P3—C1—Ir1	164.2 (3)	C208—C207—C212—C211	2.3 (9)
C307—P3—C1—Ir1	−71.7 (3)	P2—C207—C212—C211	176.6 (5)
C3—P3—C1—Ir1	44.4 (3)	C210—C211—C212—C207	−3.1 (11)
C1—P2—C2—P1	−40.0 (3)	C1—P3—C301—C302	−76.7 (5)
C201—P2—C2—P1	−168.1 (3)	C307—P3—C301—C302	153.4 (5)
C207—P2—C2—P1	79.9 (3)	C3—P3—C301—C302	41.0 (6)
C101—P1—C2—P2	147.7 (3)	C1—P3—C301—C306	101.6 (5)
C107—P1—C2—P2	−103.3 (3)	C307—P3—C301—C306	−28.3 (5)
Ir1—P1—C2—P2	22.2 (3)	C3—P3—C301—C306	−140.7 (5)
C1—P3—C3—P4	−45.9 (3)	C306—C301—C302—C303	−1.4 (9)
C301—P3—C3—P4	−170.5 (3)	P3—C301—C302—C303	176.9 (5)
C307—P3—C3—P4	77.6 (3)	C301—C302—C303—C304	0.6 (11)
C401—P4—C3—P3	153.3 (3)	C302—C303—C304—C305	0.1 (12)
C407—P4—C3—P3	−98.3 (3)	C303—C304—C305—C306	0.0 (12)
Ir1—P4—C3—P3	29.5 (3)	C302—C301—C306—C305	1.5 (10)
C107—P1—C101—C102	125.7 (5)	P3—C301—C306—C305	−176.8 (5)
C2—P1—C101—C102	−122.9 (5)	C304—C305—C306—C301	−0.8 (11)
Ir1—P1—C101—C102	−3.8 (6)	C1—P3—C307—C312	−24.2 (6)
C107—P1—C101—C106	−53.8 (5)	C301—P3—C307—C312	105.4 (5)
C2—P1—C101—C106	57.5 (5)	C3—P3—C307—C312	−140.0 (5)
Ir1—P1—C101—C106	176.6 (4)	C1—P3—C307—C308	159.1 (5)
C106—C101—C102—C103	1.0 (10)	C301—P3—C307—C308	−71.2 (5)
P1—C101—C102—C103	−178.5 (6)	C3—P3—C307—C308	43.3 (5)
C101—C102—C103—C104	−1.0 (12)	C312—C307—C308—C309	−0.1 (10)
C102—C103—C104—C105	1.4 (13)	P3—C307—C308—C309	176.5 (6)
C103—C104—C105—C106	−1.9 (12)	C307—C308—C309—C310	−1.9 (13)
C102—C101—C106—C105	−1.5 (9)	C308—C309—C310—C311	2.3 (14)
P1—C101—C106—C105	178.1 (5)	C309—C310—C311—C312	−0.8 (13)
C104—C105—C106—C101	2.0 (11)	C308—C307—C312—C311	1.5 (9)
C101—P1—C107—C108	−86.5 (5)	P3—C307—C312—C311	−175.2 (5)
C2—P1—C107—C108	163.4 (5)	C310—C311—C312—C307	−1.0 (11)
Ir1—P1—C107—C108	43.5 (5)	C3—P4—C401—C402	174.1 (5)
C101—P1—C107—C112	90.4 (5)	C407—P4—C401—C402	63.2 (5)
C2—P1—C107—C112	−19.7 (6)	Ir1—P4—C401—C402	−67.4 (5)
Ir1—P1—C107—C112	−139.6 (5)	C3—P4—C401—C406	−8.0 (5)
C112—C107—C108—C109	−0.2 (10)	C407—P4—C401—C406	−118.8 (5)
P1—C107—C108—C109	176.8 (5)	Ir1—P4—C401—C406	110.5 (4)
C107—C108—C109—C110	0.7 (11)	C406—C401—C402—C403	−0.5 (9)
C108—C109—C110—C111	−1.7 (12)	P4—C401—C402—C403	177.5 (5)
C109—C110—C111—C112	2.2 (12)	C401—C402—C403—C404	0.5 (10)
C110—C111—C112—C107	−1.8 (11)	C402—C403—C404—C405	−0.8 (10)
C108—C107—C112—C111	0.8 (10)	C403—C404—C405—C406	1.2 (10)
P1—C107—C112—C111	−176.1 (5)	C404—C405—C406—C401	−1.3 (9)
C1—P2—C201—C206	−84.0 (5)	C402—C401—C406—C405	0.9 (9)
C207—P2—C201—C206	150.1 (5)	P4—C401—C406—C405	−177.0 (5)
C2—P2—C201—C206	36.5 (5)	C3—P4—C407—C412	135.3 (5)
C1—P2—C201—C202	97.5 (5)	C401—P4—C407—C412	−112.9 (5)
C207—P2—C201—C202	−28.3 (5)	Ir1—P4—C407—C412	15.2 (5)
C2—P2—C201—C202	−142.0 (5)	C3—P4—C407—C408	−47.8 (5)
C206—C201—C202—C203	−0.3 (9)	C401—P4—C407—C408	64.0 (5)
P2—C201—C202—C203	178.3 (5)	Ir1—P4—C407—C408	−167.8 (4)
C201—C202—C203—C204	0.8 (10)	C412—C407—C408—C409	−0.2 (9)
C202—C203—C204—C205	−1.1 (11)	P4—C407—C408—C409	−177.2 (5)
C203—C204—C205—C206	0.8 (11)	C407—C408—C409—C410	0.6 (11)
C204—C205—C206—C201	−0.3 (11)	C408—C409—C410—C411	0.3 (12)
C202—C201—C206—C205	0.0 (9)	C409—C410—C411—C412	−1.7 (12)
P2—C201—C206—C205	−178.5 (5)	C408—C407—C412—C411	−1.2 (9)
C1—P2—C207—C208	−27.8 (5)	P4—C407—C412—C411	175.7 (5)
C201—P2—C207—C208	102.2 (5)	C410—C411—C412—C407	2.1 (10)

### Carbonylchloridohydrido(1,1,3,3,5,5,7,7-octaphenyl-1,5λ5,7-triphospha-3-phosphoniahept-4-en-4-yl)iridium(III) chloride–methanol–water (1/2/1) (4) Hydrogen-bond geometry (Å, º)

**Table:**

*D*—H···*A*	*D*—H	H···*A*	*D*···*A*	*D*—H···*A*
C304—H304···Cl1^i^	0.94	2.76	3.465 (7)	132
C2—H2*B*···O2	0.98	2.43	3.407 (8)	173
C210—H210···O2^ii^	0.94	2.59	3.384 (10)	143
C2—H2*A*···Cl2	0.98	2.71	3.633 (7)	157
C112—H112···Cl2	0.94	2.76	3.682 (8)	168
C3—H3*A*···Cl2*A*	0.98	2.69	3.569 (8)	150
C302—H302···O3	0.94	2.49	3.414 (14)	169

Symmetry codes: (i) −*x*+1, *y*+1/2, −*z*+3/2; (ii) *x*, −*y*+1/2, *z*+1/2.
